# Engineered polymerase-mediated efficient synthesis of site-specifically functionalized RNA and 2’-modified RNA oligonucleotides via genetic alphabet expansion

**DOI:** 10.1038/s41467-026-76347-0

**Published:** 2026-08-13

**Authors:** Jing Wu, Junyan Mei, Zhipeng Chen, Xinyu Shao, Yaxin Wang, Ziqian Xiao, Yuhui Du, Tingjian Chen

**Affiliations:** MOE International Joint Research Laboratory on Synthetic Biology and Medicines, School of Biology and Biological Engineering, South China University of Technology, Guangzhou, PR China

**Keywords:** Biochemistry, Molecular biology

## Abstract

2’-Modified RNA oligonucleotides have found broad use in biotechnology and biomedicine, requiring their efficient synthesis and controllable labelling. While engineered polymerases enable their synthesis, controllable labelling methods remain underdeveloped. Unnatural base pairs (UBPs) have been developed to expand the genetic alphabet, leading to not only the creation of semi-synthetic organisms, but also technologies for site-specific labelling of DNA and RNA. Herein we demonstrate that SFM4-6, an engineered polymerase optimized for 2’-modified RNA oligonucleotide synthesis, can efficiently synthesize unnatural base pair dNaM (2-methoxy-3-(2’-deoxy-β-D-erythro-pentofuranosyl)-naphthalene)-dTPT3 ((2’-deoxy-β-D-erythro-pentofuranosyl)-thieno[3,4]pyridine-2-thione) and its analogues, enabling site-specific incorporation of functionalized unnatural base-containing nucleotides into RNA and 2’-modified RNA oligonucleotides. By combining this with a strategy involving controlled pause and restart of primer extension, we establish methods for producing single or dual-labelled RNA and 2’-modified RNA oligonucleotides. These methods successfully produce various functional labelled RNA and 2’-modified RNA oligonucleotides, highlighting their broad applicability in nucleic acid research and biotechnology.

## Abstract (web summary)

Wu, Mei, Chen and colleagues demonstrate that an engineered XNA polymerase can effectively synthesize the unnatural base pair dNaM-dTPT3 and its analogues, enabling site-specific incorporation of functionalized unnatural base-containing nucleotides into RNA and 2′-modified RNA oligonucleotides.

## Introduction

In order to expand the functional diversity of nucleic acids, chemical modifications or substitutions have been introduced into their structural moieties, leading to the production of an emerging group of genetic polymers, xenobiotic nucleic acids (XNAs)^1–4^. For example, the hydrogen or hydroxyl group at the C2’ position of DNA and RNA molecules has been replaced through chemical synthesis by various atoms or groups, including fluorine (2’-F) and a methoxy group (2’-OMe). These modifications were found to significantly enhance the resistance of nucleic acids to nuclease degradation in biological environments and solutions, as well as their duplex stability^5–8^. Due to their diversified properties and functions, nucleic acids containing modified or unnatural sugars have found broad use in the development of therapeutic or functional nucleic acids^9,10^. Oligonucleotides, including those with modifications, are traditionally produced via solid-phase chemical synthesis. However, solid-phase chemical synthesis of RNA and modified RNA oligonucleotides has many limitations^11,12^. For example, the length of an RNA or modified RNA oligonucleotide that can be efficiently produced by solid-phase chemical synthesis is quite limited (typically ≤40 nt), and some of the modified phosphoramidites are very expensive or even not commercially available. Besides, solid-phase chemical synthesis does not synthesize an oligonucleotide by transcription from a template and thus cannot be used for the evolution of functional oligonucleotides via iterative rounds of selection^13–15^. Therefore, there is a pressing need to develop enzymatic synthesis methods for the efficient preparation of RNA and modified nucleic acids, including XNAs. To enzymatically synthesize XNAs with good efficiency, researchers have employed protein engineering strategies, such as directed evolution, to tailor natural DNA polymerases to be efficient XNA polymerases^16^. For example, the Romesberg group successfully evolved the Stoffel fragment (SF) of Taq DNA polymerase for efficient transcription, reverse transcription, and amplification of 2’-substituted nucleic acids^17–19^.

The labelling of nucleic acids is crucial for their applications in basic research, biotechnology and biomedicine, and therefore, various methods have been developed for the labelling of different nucleic acids in the past^20–23^. For example, DNA and RNA oligonucleotides whose 5’ end or 3’ end is labelled with a small reactive group have been produced by solid-phase chemical synthesis^20,24,25^. On the other hand, nucleoside and nucleotide analogues with different reactive groups have been extensively synthesized and used for the labelling of DNA and RNA^26^. Using these methods, oligonucleotides labelled with a variety of functional molecules, such as drugs and fluorophores, have been produced and applied in disease diagnosis^27^ and therapy^28^, as well as in bioimaging^29^ and molecular detection^30^. However, these methods either rely on the solid-phase chemical synthesis of labelled oligonucleotides, which has limitations as described above, or use modified nucleosides or nucleotides of natural bases, whose incorporation sites are constrained by the sequence. This underscores the urgent need for highly efficient methods for controllable labelling of nucleic acids at precise sites. It is worth noting that, in recent years, unnatural base pairs (UBPs) have emerged to provide promising tools for site-specific labelling of nucleic acids^31^.

UBPs have been developed to expand the genetic alphabet in the past decades^31–35^. The unnatural bases that compose a UBP pair with each other orthogonally to the pairing between natural bases, and thus allow site-specific incorporation of functionalities into nucleic acids^36^. Several DNA and RNA polymerases have been proven to be able to efficiently replicate or transcribe UBP dNaM-dTPT3 and its analogues^37,38^, and many studies have been focused on using functionalized derivatives of dNaM-dTPT3 or its analogues for site-specific labelling of DNA and RNA^39^. Previously, we systematically explored the incorporation of dNaMTP, dTPT3TP, and their analogues into various nucleic acids by a variety of polymerases^40–44^. Based on the recognition of unnatural substrates by different terminal deoxynucleotidyl transferases, functionalized derivatives of dTPT3TP and rTPT3TP have been used in the 3’-end labelling of DNA and a few 2’-modified nucleic acids^40,43^. Although UBPs have already demonstrated great potential in the labelling of DNA and RNA, little effort has been made in investigating the activities of the engineered XNA polymerases for UBP synthesis, which would allow the preparation of nucleic acids possessing both unnatural sugar-phosphate backbones for enhanced stability and unnatural bases for site-specific functionalization once verified. As a rare example, the enzymatic synthesis of threose nucleic acids (TNAs) containing TPT3 base was achieved, without further demonstration of site-specific functionalization of these molecules through the unnatural base^45^.

Herein, we first explored and demonstrated the activity of SF mutant SFM4-6, which has been evolved to be efficient for the synthesis of 2’-modified nucleic acids^17^, for the replication of UBP dNaM-dTPT3 and its analogues. SFM4-6’s activities for incorporating deoxyribonucleoside triphosphate dTPT3TP, ribonucleoside triphosphate rTPT3TP, and their analogues opposite a dNaM or natural nucleotide in the DNA template were systematically characterized. Based on this, we developed a method for the synthesis of RNA and 2’-modified RNA oligonucleotides site-specifically incorporating a d/rTPT3 nucleotide or one of their functional analogues with a dNaM-containing DNA template and SFM4-6. Meanwhile, we established a method for the preparation of 5’-end-unnatural base (UB)-labelled RNA and 2’-modified RNA oligonucleotides with a natural DNA template via controlled pause and restart primer extension. Moreover, dual-labelled 2’-F-CU-RNA (both cytidine and uridine residues in the sequence are modified with a 2’-fluoro substitution) oligonucleotides were also prepared, further demonstrating the powerful capability of the combined use of SFM4-6 and UBP for producing site-specifically labelled RNA and 2’-modified RNA oligonucleotides with good precision and universality. To show the value of the established methods in practical application, we prepared several functional RNA and 2’-modified RNA oligonucleotides labelled with these methods, including an siRNA, several aptamers and an aptasensor, with their functions successfully demonstrated.

## Results and discussion

### Recognition of UBP dNaM-dTPT3 and its analogues by SFM4-6

**Fig. 1:**
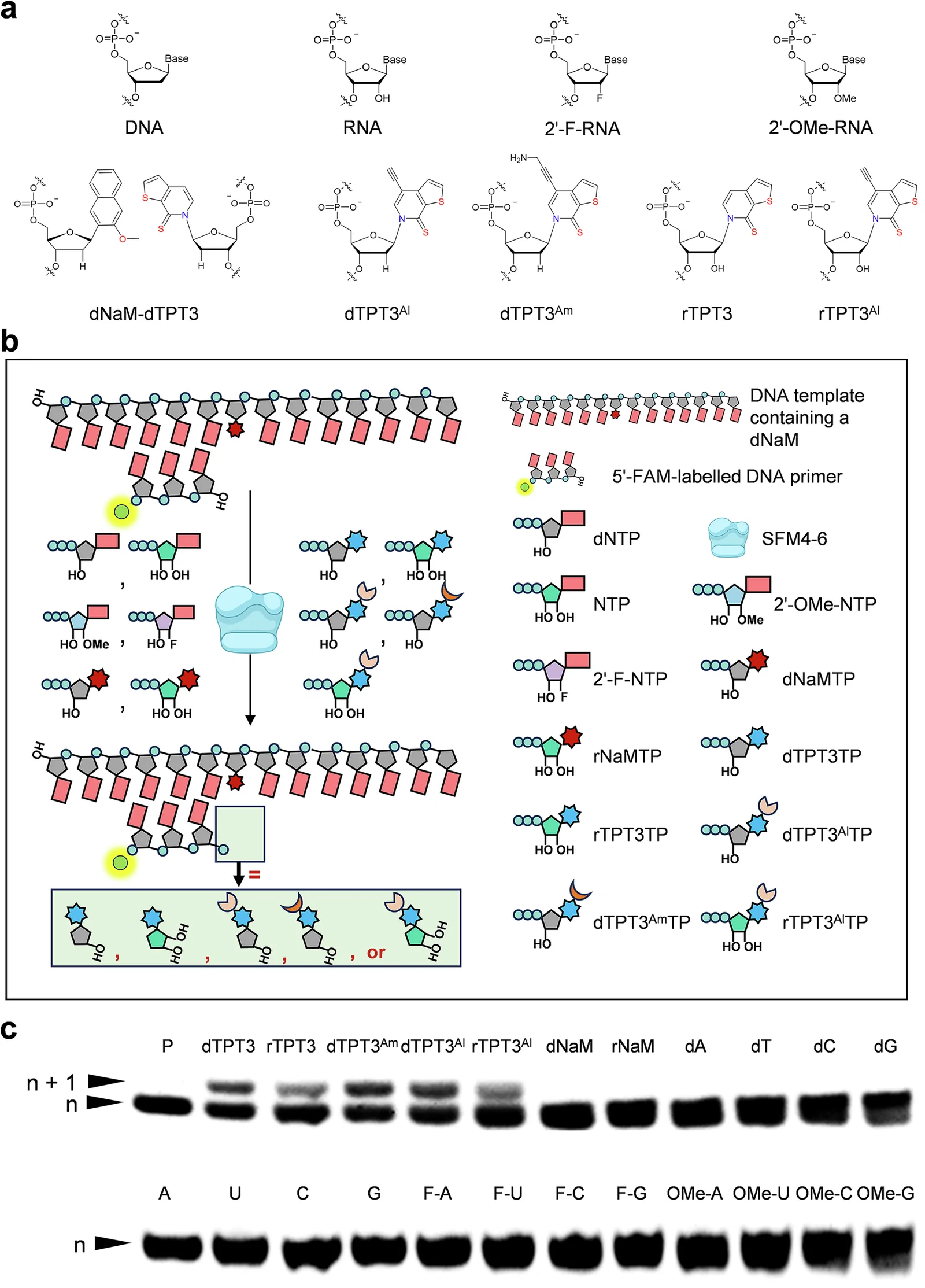
SFM4-6-mediated specific incorporation of nucleoside triphosphates containing d/rTPT3 or their analogues opposite a dNaM nucleotide in the DNA template. **a** Chemical structures of DNA, RNA, 2’-F-RNA, 2’-OMe-RNA, UBP dNaM-dTPT3, unnatural nucleotides dTPT3^Al^, dTPT3^Am^, rTPT3, and rTPT3^Al^. **b** Schematic diagram of the primer extension assay for SFM4-6-mediated incorporation of natural or unnatural nucleoside triphosphates opposite the dNaM nucleotide in the DNA template. **c** Gel analysis for the products of primer extension with nucleoside triphosphate dTPT3TP, rTPT3TP, dTPT3^Am^TP, dTPT3^Al^TP, rTPT3^Al^TP, dNaMTP, rNaMTP, each one of natural dNTPs, each one of natural NTPs, each one of 2’-F-NTPs, or each one of 2’-OMe-NTPs. The products were analysed with 20% denaturing PAGE gels containing 8 M urea, and the gels were directly visualized without staining. n: position for primer FAM-P-20 (20 nt); n + 1: position for primer FAM-P-20 incorporating one more nucleotide (21 nt). Lane P: primer FAM-P-20. Lanes dTPT3, rTPT3, dTPT3^Am^, dTPT3^Al^, and rTPT3^Al^: primer extension with dTPT3TP, rTPT3TP, dTPT3^Am^TP, dTPT3^Al^TP, or rTPT3^Al^TP, respectively. Lanes dNaM and rNaM: primer extension with dNaMTP or rNaMTP, respectively. Lanes dA, dT, dC, and dG: primer extension with dATP, dTTP, dCTP, or dGTP, respectively. Lanes A, U, C, and G: primer extension with ATP, UTP, CTP, or GTP, respectively. Lanes F-A, F-U, F-C, and F-G: primer extension with 2’-F-ATP, 2’-F-UTP, 2’-F-CTP, or 2’-F-GTP, respectively. Lanes OMe-A, OMe-U, OMe-C, and OMe-G: primer extension with 2’-OMe-ATP, 2’-OMe-UTP, 2’-OMe-CTP, or 2’-OMe-GTP, respectively. Source data are provided in the Source Data file. Figure created in part with BioRender.com. Mei, J. (2026) https://BioRender.com/v4y9m7u.

Previous studies have shown that several DNA polymerases and RNA polymerases from different families are capable of recognizing UBP dNaM-dTPT3 and its analogues and conducting their replication or transcription^37,38,40,41,46^. To investigate whether SFM4-6, a representative evolved SF mutant efficient for the synthesis of 2’-modified nucleic acids, can also recognize dNaM-d/rTPT3TP (rTPT3 denotes the ribonucleoside of dTPT3) and its analogues (Fig. 1a) and conduct their replication during the synthesis of different natural and 2’-modified nucleic acids, we first carried out the primer extension experiment to test and compare the incorporation efficiencies of different natural and unnatural nucleoside triphosphates opposite a dNaM nucleotide in a DNA template by SFM4-6. 5’-End-6-carboxyfluorescein (FAM)-labelled primer FAM-P-20 was annealed to a dNaM-containing DNA template, T55-X (Supplementary Table 1), and then extended with SFM4-6 in the presence of one of different nucleoside triphosphates (dTPT3TP, rTPT3TP, dTPT3^Am^TP, dTPT3^Al^TP, rTPT3^Al^TP, dNaMTP, rNaMTP, dNTPs, NTPs, 2’-F-NTPs, and 2’-OMe-NTPs) (Fig. 1a, b). Gel analysis of the products revealed that under the same reaction conditions, when dTPT3TP, rTPT3TP, or any of their functionalized analogues was included in the reaction solution, the primer was efficiently extended by the incorporation of a nucleotide opposite the dNaM nucleotide in the DNA template (Fig. 1c). In sharp contrast, no obvious primer extension was observed when any of other nucleoside triphosphate was included in the reaction solution. This result clearly indicates that SFM4-6 could efficiently and specifically incorporate dTPT3TP, rTPT3TP, or any of their analogues opposite a dNaM nucleotide in the DNA template.

To quantitatively characterize the activities of SFM4-6 in synthesizing UBPs dNaM-dTPT3, dNaM-rTPT3, dNaM-dNaM, and dNaM-rNaM, steady-state kinetic experiments were carried out. 5’-End-FAM-labelled primer FAM-P-20 was extended by SFM4-6 through the incorporation of dTPT3TP, rTPT3TP, dNaMTP, or rNaMTP opposite a dNaM nucleotide in DNA template T55-X under varying reaction conditions. To compare the efficiencies of SFM4-6 for the synthesis of these UBPs with those for the synthesis of the natural base pairs, primer FAM-P-20 was also extended with SFM4-6 polymerase by incorporating dTTP or UTP opposite a dA nucleotide in DNA template T55-A (Supplementary Table 1) under varying reaction conditions. The products were analyzed with 20% denaturing PAGE (polyacrylamide gel electrophoresis) gels containing 8 M urea. The fitted Michaelis-Menten plots are shown in Supplementary Fig. 1, and the calculated *K*_m_, *k*_cat_, and *k*_cat_/*K*_m_ values for SFM4-6-mediated incorporation of one of the nucleoside triphosphates containing an unnatural or natural base (dTPT3TP, rTPT3TP, dNaMTP, rNaMTP, dTTP, and UTP) opposite the corresponding nucleotide are summarized in Table 1. The catalytic efficiencies (reflected by the *k*_cat_/*K*_m_ values) for the synthesis of dNaM-dTPT3 and dNaM-rTPT3 are close to those for the synthesis of the natural base pairs (dA-dT and dA-U), whereas the catalytic efficiencies for the synthesis of self-pairs dNaM-dNaM and dNaM-rNaM are 184 and 282-fold lower, respectively. This again suggests the high efficiency and specificity of SFM4-6-mediated synthesis of dNaM-dTPT3 and dNaM-rTPT3. The order for the synthesis efficiencies of different base pairs is as follows: dA-dT > dA-U ≈ dNaM-dTPT3 > dNaM-rTPT3 » dNaM-dNaM > dNaM-rNaM.

**Table 1:** Steady-state kinetic constants for SFM4-6-mediated incorporation of different unnatural and natural nucleoside triphosphates opposite a dNaM or dA nucleotide in the DNA template

3’-CCGAAGCATACAACACACCTTACGGGAGXTCCGTTGTTAAAGTGTGTCCTTTGTC-5’ 5’-FAM-ATGTTGTGTGGAATGCCCTC				
X	Nucleoside triphosphate	*K*_m_ (μM)	*k*_cat_ (min^−1^)	*k*_cat_/*K*_m_ (mM^−1^ min^−1^)
NaM	dTPT3TP	0.22 ± 0.014	29.31 ± 0.45	1.33 × 10^5^
	rTPT3TP	0.83 ± 0.14	20.43 ± 1.16	2.46 × 10^4^
	dNaMTP	1.69 ± 0.22	1.75 ± 0.14	1.04 × 10^3^
	rNaMTP	2.32 ± 0.16	1.14 ± 0.12	0.49 × 10^3^
A	dTTP	0.17 ± 0.067	32.52 ± 0.84	1.91 × 10^5^
	UTP	0.26 ± 0.12	35.82 ± 1.30	1.38 × 10^5^

### SFM4-6-mediated synthesis of RNA, 2’-F-CU-RNA, 2’-F/OMe-RNA, and 2’-OMe-RNA oligonucleotides site-specifically incorporating a nucleotide of dTPT3, rTPT3, or one of their analogues

**Fig. 2:**
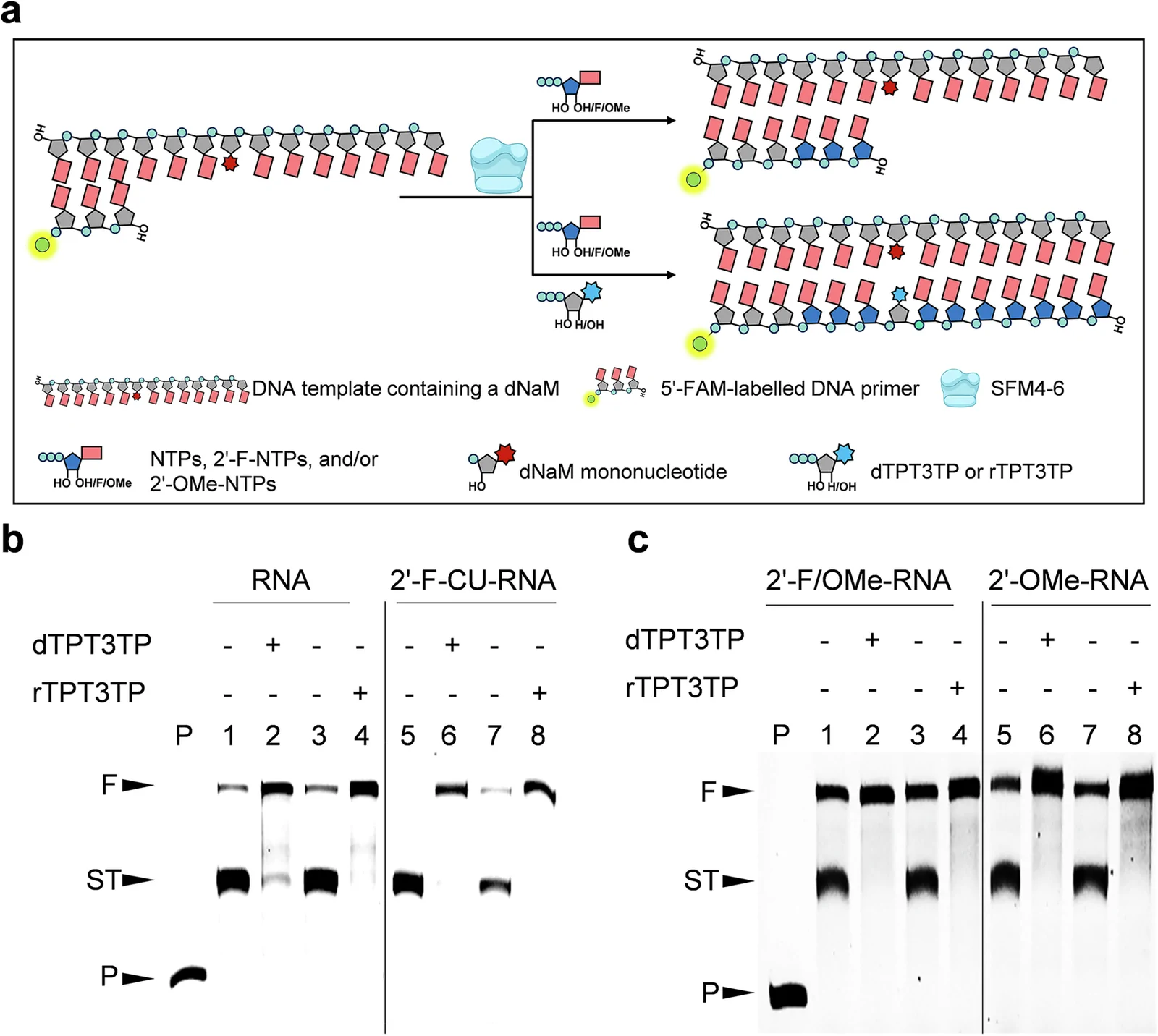
Primer extension assay for SFM4-6-mediated synthesis of RNA and 2’-modified RNA oligonucleotides site-specifically incorporating a dTPT3 or rTPT3 nucleotide using a dNaM-containing DNA template. **a** Schematic diagram of the primer extension assay for the synthesis of RNA, 2’-F-CU-RNA, 2’-F/OMe-RNA, and 2’-OMe-RNA site-specifically incorporating a dTPT3 or rTPT3 nucleotide. **b** Gel analysis for the products of primer extension with NTPs; or ATP, 2’-F-UTP, 2’-F-CTP, and GTP in the absence or presence of dTPT3TP and/or rTPT3TP. **c** Gel analysis of the primer extension products produced with 2’-OMe-ATP, 2’-F-UTP, 2’-F-CTP, and 2’-OMe-GTP; or 2’-OMe-NTPs in the absence or presence of dTPT3TP and/or rTPT3TP. For both panels **b** and **c**, the products were analyzed with 20% denaturing PAGE gels containing 8 M urea, and the gels were directly visualized without staining. P: position for primer FAM-P-18 (18 nt); ST: position for the product of primer extension stalled at the position opposite the dNaM nucleotide in the template; F: position for the full-length primer extension product. Source data are provided in the Source Data file. Figure created in part with BioRender.com. Mei, J. (2026) https://BioRender.com/v4y9m7u.

With the activity of SFM4-6 for the synthesis of UBPs dNaM-dTPT3 and dNaM-rTPT3 shown and characterized, we further carried out primer extension experiments to investigate the capability of SFM4-6 to specifically incorporate dTPT3TP, rTPT3TP or one of their analogues opposite a dNaM nucleotide in the DNA template during the synthesis of RNA or 2’-modified RNA oligonucleotides with a DNA template. 5’-End-FAM-labelled primer FAM-P-18 (Supplementary Table 1) was extended with different combinations of NTPs and 2’-modified NTPs with dNaM-containing DNA template T55-X, in the absence or presence of dTPT3TP and/or rTPT3TP (Fig. 2a). Gel analysis of the products showed that for the synthesis of the RNA or 2’-F-CU-RNA oligonucleotide, almost all of the primer was efficiently extended to the full length in the presence of dTPT3TP or rTPT3TP, while the extension of a large proportion of the primer stalled at the position opposite the dNaM nucleotide in the template in the absence of dTPT3TP and rTPT3TP (Fig. 2b). This indicates that the dTPT3 or rTPT3 nucleotide was efficiently incorporated into the synthesized RNA or 2’-F-CU-RNA oligonucleotide, specifically at the position opposite the dNaM nucleotide in the template. For the synthesis of the 2’-F/OMe-RNA and 2’-OMe-RNA oligonucleotides, also almost all of the primer was efficiently extended to the full length in the presence of dTPT3TP or rTPT3TP (Fig. 2c). In the absence of dTPT3TP and rTPT3TP, although the extension of a large proportion of the primer also stalled at the position opposite the dNaM nucleotide in the template, a substantial amount of full-length primer extension product was observed, indicating an increase in the nonspecific incorporation of 2’-modified NTPs opposite the dNaM nucleotide, which might be attributed to the supplemented MnCl_2_, prolonged reaction time or thermal cycling programme employed for the synthesis of 2’-F/OMe-RNA and 2’-OMe-RNA. Overall, these results suggest that SFM4-6 is capable of efficiently synthesizing RNA and 2’-modified RNA oligonucleotides incorporating a dTPT3 or rTPT3 nucleotide at a specific position with good or moderate specificity. The full-length primer extension products produced in the presence of dTPT3TP or rTPT3TP were further treated with DNase I, and the result showed that the DNA primer and template could be effectively removed by DNase I, without obvious cleavage of the RNA and 2’-modified RNA oligonucleotides at the position of dTPT3 or rTPT3 nucleotide (Supplementary Fig. 2). This indicates the tolerance of the incorporated dTPT3 or rTPT3 nucleotide to DNase I degradation, allowing the production of pure RNA and 2’-modified RNA oligonucleotides incorporating a dTPT3 or rTPT3 nucleotide at a specific position.

To test if SFM4-6 is also able to synthesize RNA and 2’-modified RNA oligonucleotides incorporating a nucleotide of one of the functionalized analogues of dTPT3 and rTPT3 with a dNaM-containing DNA template, we carried out the same primer extension experiments as described above, except that the dTPT3TP or rTPT3 was replaced with one of their analogues whose base is modified with a propargylamino or ethynyl group (dTPT3^Am^TP, dTPT3^Al^TP, and rTPT3^Al^TP). Gel analysis of the products showed that all of dTPT3^Am^TP, dTPT3^Al^TP, and rTPT3^Al^TP can be successfully incorporated into all of the synthesized RNA and 2’-modified RNA oligonucleotides by SFM4-6, with varied efficiency and specificity, and the order for the synthesis efficiencies of different unnatural base-containing oligonucleotides is as follows: RNA > 2’-F-CU-RNA > 2’-F/OMe-RNA > 2’-OMe-RNA (Supplementary Fig. 3). By optimizing the reaction conditions, the yields of the full-length products for the dTPT3^Am^, dTPT3^Al^, or rTPT3^Al^-containing 2’-F/OMe-RNA, and 2’-OMe-RNA oligonucleotides can be effectively improved. For example, when the reaction time was increased from 4 h (Supplementary Fig. 3) to 17 h, and a thermocycling programme was applied to the reaction (Supplementary Fig. 4), for each of the dTPT3^Am^, dTPT3^Al^, or rTPT3^Al^-containing 2’-F/OMe-RNA and 2’-OMe-RNA oligonucleotides, the yield of the full-length product was significantly increased, and only a little or almost no byproduct was observed in the presence of dTPT3^Am^TP, dTPT3^Al^TP, or rTPT3^Al^TP (Supplementary Fig. 4). Then mass spectrometry analysis was performed to confirm the incorporation of the nucleotide of the unnatural base into the 2’-F/OMe-RNA and 2’-OMe-RNA oligonucleotides produced in the presence of the nucleoside triphosphate of the unnatural base and to investigate the identities of the oligonucleotides produced in the absence of the nucleoside triphosphate of the unnatural base. The 2’-F/OMe-RNA and 2’-OMe-RNA oligonucleotides produced in the absence and presence of rTPT3^Al^TP were analysed as examples, and for each oligonucleotide, the DNA primer and template were removed by DNase I treatment, and the product was purified before the mass spectrometry analysis. The results clearly demonstrated that the rTPT3^Al^ nucleotide was correctly and efficiently incorporated into the produced oligonucleotides in the presence of rTPT3^Al^TP, and in the absence of rTPT3^Al^TP, a 2’-F-U or 2’-OMe-A nucleotide was incorporated opposite the dNaM in the template instead (Supplementary Figs. 17 and 18). Next, the full-length primer extension products produced in the presence of dTPT3^Am^TP, dTPT3^Al^TP, or rTPT3^Al^TP were treated with DNase I, and then the degradation products were purified and coupled with 6-carboxyfluorescein N-succinimidyl (6-FAM-NHS) ester (for the product produced with dTPT3^Am^TP) or Alexa Fluor 488-azide (AF488-N_3_, for the products produced with dTPT3^Al^TP or rTPT3^Al^TP), respectively. Gel analysis of the products again showed that the DNA primer and template could be effectively removed by DNase I, without obvious cleavage of the RNA and 2’-modified RNA oligonucleotides at the position of dTPT3^Am^, dTPT3^Al^, or rTPT3^Al^ nucleotide (Supplementary Fig. 5). This indicates the tolerance of the incorporated dTPT3^Am^, dTPT3^Al^, or rTPT3^Al^ nucleotide to DNase I degradation, allowing the production of pure RNA and 2’-modified RNA oligonucleotides incorporating a dTPT3^Am^, dTPT3^Al^, or rTPT3^Al^ nucleotide at a specific position. For all of the DNase I degradation-produced RNA and 2’-modified RNA oligonucleotides coupled with 6-FAM-NHS ester or AF488-N_3_, a sharp and bright band was observed before the gels were stained with Cyber gold (a SYBR Gold analogue, with full specifications in the “Materials” subsection in the “Methods” section), indicating that the dTPT3^Am^, dTPT3^Al^, or rTPT3^Al^ nucleotide remained intact and reactive after the preparation of the oligonucleotides, and the produced RNA and 2’-modified RNA oligonucleotides can be site-specifically labelled or functionalized via dTPT3^Am^, dTPT3^Al^, or rTPT3^Al^ with excellent efficiency.

### SFM4-6-mediated synthesis of 5’-end-unnatural base (UB)-labelled RNA, 2’-F-CU-RNA, 2’-F/OMe-RNA, and 2’-OMe-RNA oligonucleotides via controlled pause and restart of primer extension with a natural DNA template

**Fig. 3:**
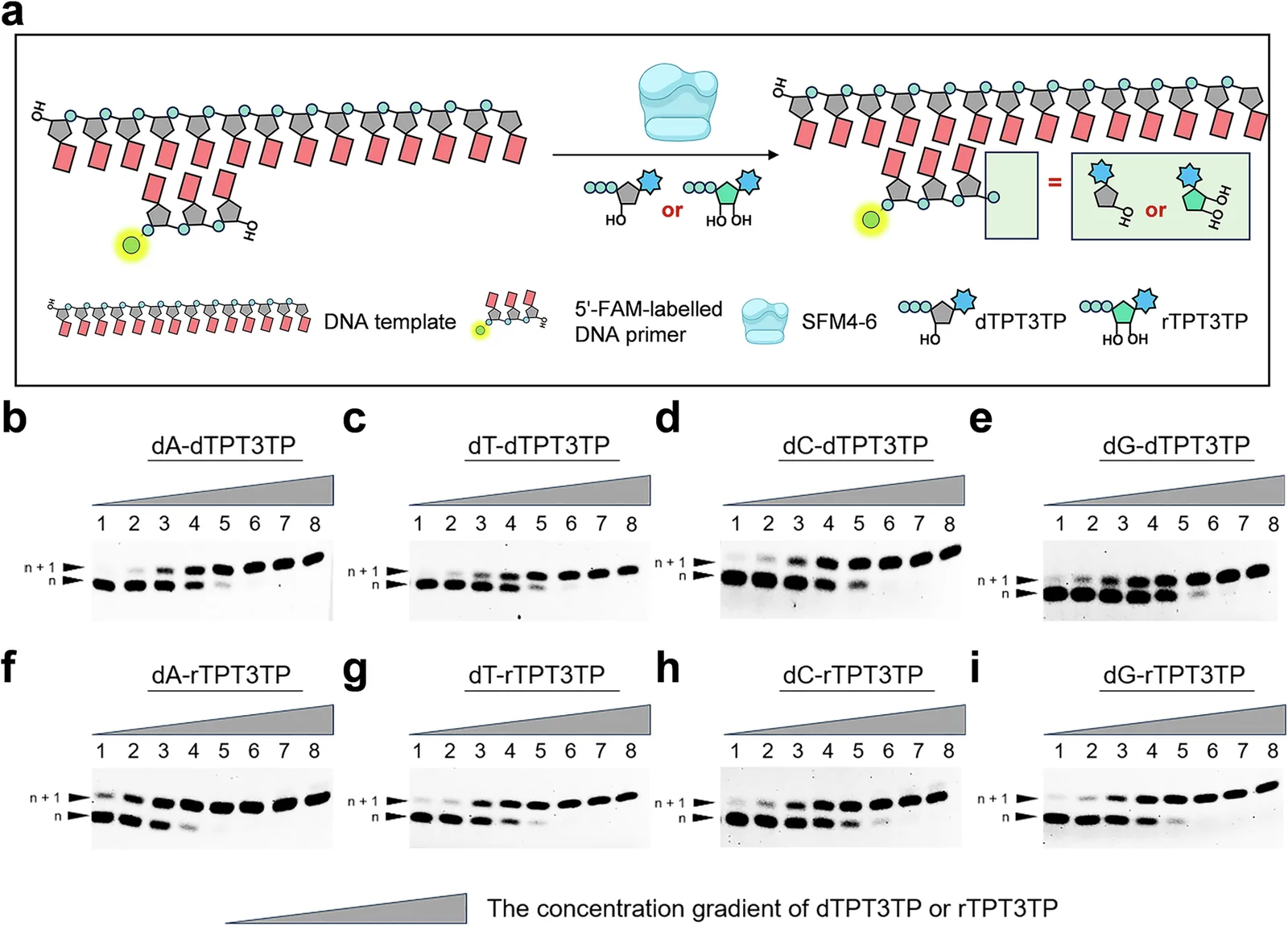
SFM4-6-mediated incorporation of dTPT3TP or rTPT3TP opposite a natural nucleotide in the DNA template. **a** Schematic diagram of the primer extension assay for SFM4-6-mediated incorporation of dTPT3TP or rTPT3TP opposite a natural nucleotide in different DNA templates. **b–i **Gel analysis for the products of primer extension with DNA template T55-A (**b**), T55-T (**c**), T55-C (**d**), or T55-G (**e**) for the incorporation of dTPT3TP opposite a dA, dT, dC, or dG nucleotide in the DNA template. Gel analysis of the products of primer extension with DNA template T55-A (**f**), T55-T (**g**), T55-C (**h**), or T55-G (**i**) for the incorporation of rTPT3TP opposite a dA, dT, dC, or dG nucleotide in the DNA template. The products were analyzed with 20% denaturing PAGE gels containing 8 M urea, and the gels were directly visualized without staining. Lanes 1–8: primer extension with 12.5, 25, 50, 100, 150, 200, 300, or 400 nM dTPT3TP or rTPT3TP, respectively. n: position for primer FAM-P-20 (20 nt); n + 1: position for primer FAM-P-20 incorporating one more nucleotide (21 nt). Source data are provided in the Source Data file. Figure created in part with BioRender.com. Mei, J. (2026) https://BioRender.com/v4y9m7u.

5’-End-labelling of oligonucleotides is essential for many of their applications^20,47^. In principle, the method for the synthesis of RNA and 2’-modified RNA oligonucleotides site-specifically incorporating a nucleotide of dTPT3, rTPT3, dTPT3^Am^, dTPT3^Al^, or rTPT3^Al^ established above could also be applied for the preparation of 5’-end-UB-labelled RNA and 2’-modified RNA oligonucleotides, simply by placing the templating dNaM nucleotide at the position opposite where the first nucleotide would be incorporated for the primer extension. However, this method relies on the use of a dNaM-containing DNA template, which traditionally needs to be synthesized by solid-phase chemical synthesis with the phosphoramidite of dNaM, or prepared enzymatically^40–42,48^. Previously, we have established a method for the synthesis of unnatural base-containing DNA oligonucleotides via controlled pause and restart of primer extension with a natural DNA template^41^. Here, we attempted to establish a similar method to synthesize the 5’-end-UB-labelled RNA and 2’-modified RNA oligonucleotides via controlled pause and restart of primer extension with a natural DNA template. In this method, the primer is extended first by incorporating one single nucleotide of the unnatural base opposite a natural nucleotide in the DNA template, and then natural or/and 2’-modified ribonucleoside triphosphates were directly added into the reaction solution to restart the primer extension and complete the synthesis of the RNA or 2’-modified RNA oligonucleotide. We first tested and optimized the reaction conditions for the incorporation of a single nucleotide of dTPT3 or rTPT3 opposite a varying natural nucleotide in the DNA template by SFM4-6 (Fig. 3a). As shown in Fig. 3b–i, with different DNA templates harbouring different natural nucleotides at the position opposite where the nucleotide of dTPT3 or rTPT3 would be incorporated, when a proper concentration (100–400 nM) of dTPT3TP or rTPT3TP was applied, the primer could be efficiently extended by incorporating a single nucleotide of dTPT3 or rTPT3, with no obvious unextended primer nor undesired byproducts observed.

**Fig. 4:**
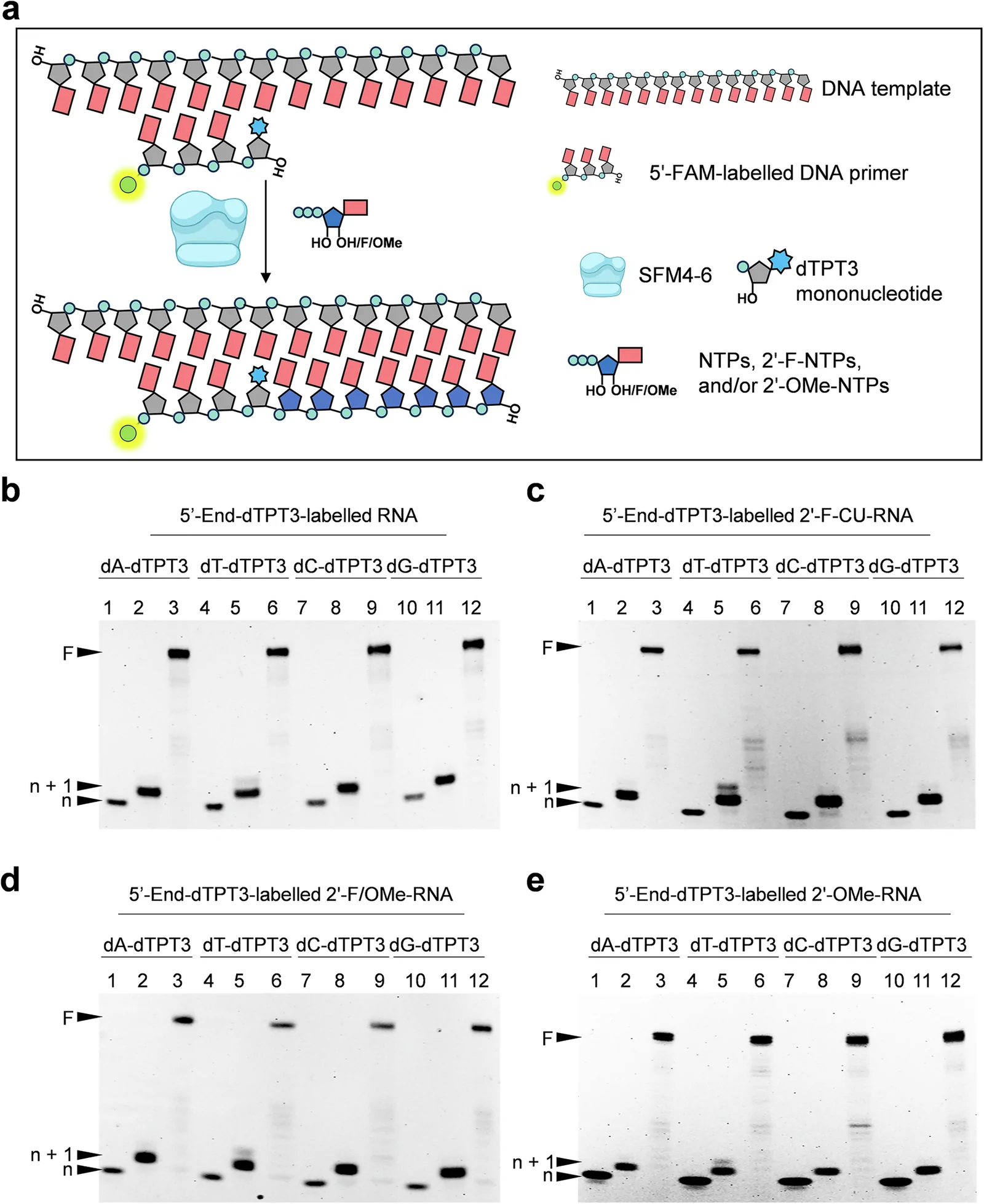
SFM4-6-mediated primer extension with controlled pause and restart for the enzymatic synthesis of 5’-end-dTPT3-labelled RNA and 2’-modified RNA oligonucleotides. **a** Schematic diagram of SFM4-6-mediated primer extension by the synthesis of RNA, 2’-F-CU-RNA, 2’-F/OMe-RNA, or 2’-OMe-RNA after the incorporation of a dTPT3 nucleotide opposite a natural nucleotide in the DNA template. **b–e** Gel analysis for the intermediate and final products of SFM4-6-mediated primer extension by the synthesis of RNA, 2’-F-CU-RNA, 2’-F/OMe-RNA, or 2’-OMe-RNA after the incorporation of a dTPT3 nucleotide opposite a natural nucleotide (dA, dT, dC, or dG nucleotide) in the DNA template. Lanes 1-12: the products for the synthesis of 5’-end-dTPT3-labelled RNA and 2’-modified RNA oligonucleotides using template T55-A (lanes 1–3), T55-T (lanes 4–6), T55-C (lanes 7–9), or T55-G (lanes 10–12), respectively. All the products were analyzed with 20% denaturing PAGE gels containing 8 M urea, and the gels were directly visualized without staining. Lanes 1, 4, 7, and 10: FAM-labelled DNA primer FAM-P-20; lanes 2, 5, 8, and 11: FAM-P-20 extended by the incorporation of a dTPT3 nucleotide opposite a dA, dT, dC, or dG nucleotide in the DNA template; lanes 3, 6, 9, and 12: FAM-P-20 extended with NTPs (**b**), ATP, 2’-F-UTP, 2’-F-CTP, and GTP (**c**), 2’-OMe-ATP, 2’-F-UTP, 2’-F-CTP, and 2’-OMe-GTP (**d**), or 2’-OMe-NTPs (**e**) after the incorporation of a dTPT3 nucleotide opposite a dA, dT, dC, or dG nucleotide in the DNA template. n: position for primer FAM-P-20 (20 nt); n + 1: position for primer FAM-P-20 incorporating a dTPT3 nucleotide (21 nt); F: full-length product of the primer extension. Source data are provided in the Source Data file. Figure created in part with BioRender.com. Mei, J. (2026) https://BioRender.com/v4y9m7u.

Next, we tested if the primer extension could be restarted by the direct addition of natural or/and 2’-modified ribonucleoside triphosphates into the reaction solution, and if the full-length RNA or 2’-modified RNA oligonucleotide product could be efficiently synthesized by SFM4-6 then. After the incorporation of the dTPT3 or rTPT3 nucleotide under the optimized conditions as described above, the natural or/and 2’-modified ribonucleoside triphosphates were added into the reaction solution to restart the primer extension by the synthesis of RNA, 2’-F-CU-RNA, 2’-F/OMe-RNA, or 2’-OMe-RNA oligonucleotide (Fig. 4a and Supplementary Fig. 6a). Gel analysis of the products revealed that for all of the cases, the full-length primer extension products could be efficiently synthesized by SFM4-6 (Fig. 4b–e and Supplementary Fig. 6b–e). This suggests that the 5’-end-dTPT3 or rTPT3-labelled RNA and 2’-modified RNA oligonucleotides could be successfully synthesized via controlled pause and restart of primer extension with a natural DNA template and SFM4-6.

Steady-state kinetic experiments were further carried out to rigorously characterize the SFM4-6-mediated incorporation of dTPT3TP or rTPT3TP opposite different natural nucleotides in the DNA template and to quantitatively prove that after the incorporation of the dTPT3 or rTPT3 nucleotide, the supplemented natural or 2’-modified ribonucleoside triphosphates are more efficiently incorporated opposite their complementary nucleotides in the template than dTPT3TP or rTPT3TP, during the synthesis of the RNA or 2’-modified RNA oligonucleotides. First, steady-state kinetic experiments were carried out for SFM4-6-mediated incorporation of dTPT3TP or rTPT3TP opposite a dA, dT, dC, or dG nucleotide in the DNA template. As shown in Supplementary Table 2, Supplementary Figs. 7 and 8, SFM4-6 showed measurable and varied efficiencies (reflected by the *k*_cat_/*K*_m_ values) for the incorporation of dTPT3TP and rTPT3TP opposite different natural nucleotides in the DNA template, although approximately 45- to 190-fold lower than those for the incorporation of dTPT3TP and rTPT3TP opposite a dNaM nucleotide (Table 1). The order for the synthesis efficiencies of different natural-unnatural mispairs is as follows: dA-dTPT3 > dT-dTPT3 > dG-dTPT3 > dC-dTPT3 > dA-rTPT3 > dT-rTPT3 > dG-rTPT3 > dC-rTPT3. This suggests that a DNA template with a dA nucleotide at the position opposite where the dTPT3TP or rTPT3TP would be incorporated should be optimal for the synthesis of 5’-end-dTPT3 or rTPT3-labelled RNA and 2’-modified RNA oligonucleotides via controlled pause and restart of primer extension. Next, steady-state kinetic experiments were carried out for SFM4-6-mediated incorporation of ATP, 2’-F-ATP, 2’-OMe-ATP, or dTPT3TP opposite a dT nucleotide at the n + 2 position in the DNA template after the primer was extended by the incorporation of a dTPT3 nucleotide opposite a dA nucleotide at the n + 1 position. As shown in Supplementary Table 2 and Supplementary Fig. 9, the efficiencies for the incorporation of ATP, 2’-F-ATP, and 2’-OMe-ATP are 1–2 orders of magnitude higher than that for the incorporation of dTPT3TP, suggesting little effect of the remaining dTPT3TP in the reaction solution on the synthesis of RNA and 2’-modified RNA oligonucleotides during the restarted primer extension in the method of synthesizing 5’-end-UB-labelled RNA and 2’-modified RNA oligonucleotides via controlled pause and restart of primer extension with a natural DNA template and SFM4-6. For all of the steady-state kinetic experiments described above, we followed the reported method for determining the steady-state kinetic constants of polymerases^49–51^. To rigorously validate that, in these experiments, the time points that we selected to measure the initial rates (when the extension percentages of the primer were ~20%) were within the linear phase of the reaction, we measured the time course of SFM4-6-mediated primer extension by the incorporation of dTPT3TP opposite a dA nucleotide in DNA template T55-A. The primer extension reaction was carried out with 64 µM dTPT3TP, the extension products at different reaction times (0–360 s) were analyzed by 20% denaturing PAGE gels, and the extension percentages of the primer were plotted against the reaction times (Supplementary Fig. 10). Linear fitting of the data in the range of 1–120 s, where the extension percentages of the primer were less or equal to ~20%, revealed a good linearity of the reaction curve in this range (*R*^2^ = 0.9928), suggesting that the time points that we selected to measure the initial rates were within the linear phase of the reaction, and the method used to determine the steady-state kinetic constants is reliable.

We then tested the usability of the same strategy for the preparation of 5’-end-dTPT3^Am^, dTPT3^Al^, or rTPT3^Al^-labelled RNA and 2’-modified RNA oligonucleotides, which could be further labelled or functionalized via coupling with NHS ester or azide-conjugated molecules for various practical applications. Experiments of primer extension with controlled pause and restart were carried out with the same protocol as described above, except that in the first step (incorporation of a single nucleotide of the unnatural base), dTPT3TP or rTPT3TP was replaced by dTPT3^Am^TP, dTPT3^Al^TP, or rTPT3^Al^TP, and only DNA template T55-A was used for testing (Supplementary Fig. 11a). The products were also analyzed with denaturing PAGE gels, and the result again demonstrated the efficient synthesis of all the 5’-end-dTPT3^Am^, dTPT3^Al^, or rTPT3^Al^-labelled RNA and 2’-modified RNA oligonucleotides (Supplementary Fig. 11b, c). The primer extension products were treated with DNase I to remove the DNA primer and template, and then mass spectrometry analysis was performed to verify that the desired oligonucleotides had been precisely produced (Supplementary Figs. 19–30).

Next, the 5’-end-UB-labelled RNA and 2’-modified RNA oligonucleotide products were coupled with 6-FAM-NHS ester (for dTPT3^Am^-labelled oligonucleotide products), biotin-PEG_4_-NHS ester (for dTPT3^Am^-labelled oligonucleotide products), AF488-N_3_ (for dTPT3^Al^ or rTPT3^Al^-labelled oligonucleotide products), or biotin-PEG_3_-azide (biotin-PEG_3_-N_3_, for dTPT3^Al^ or rTPT3^Al^-labelled oligonucleotide products), respectively (Supplementary Figs. 12a and 13a). The FAM or AF488-labelled oligonucleotide products were analyzed with 20% denaturing PAGE gels containing 8 M urea. For all of the products, a sharp fluorescent band was observed on the gel before the gels were stained with Cyber gold (Supplementary Fig. 12b–g), suggesting that the 5’-end dTPT3^Am^, dTPT3^Al^, and rTPT3^Al^ nucleotides in the oligonucleotide products are tolerant to DNase I, and the 5’-end-dTPT3^Am^, dTPT3^Al^, or rTPT3^Al^-labelled RNA and 2’-modified RNA oligonucleotides were successfully prepared and efficiently labelled with the fluorophore-coupled NHS ester or azide. The biotin-labelled oligonucleotide products were further purified and an aliquot of each product was incubated with streptavidin (SA) before all the products were analyzed with 9% PAGE gels (Supplementary Fig. 13a). As shown in Supplementary Fig. 13b–e, for all of the biotin-labelled oligonucleotide products, 100% of the band was shifted upon incubation of the oligonucleotide product with SA, indicating that 100% of each of the prepared RNA and 2’-modified RNA oligonucleotides contained the 5’-end dTPT3^Am^, dTPT3^Al^, or rTPT3^Al^ nucleotide, and 100% of each of them was successfully labelled with biotin. Clearly, the established method demonstrated excellent efficiency for the production of 5’-end-dTPT3^Am^, dTPT3^Al^, or rTPT3^Al^-labelled RNA and 2’-modified RNA oligonucleotides and further labelling or functionalization of them. This, together with the simplicity, rapidness, and universality, would allow this method to find broad use in the labelling of nucleic acids.

### Preparation of a 5’-end-fluorescent-labelled and functional siRNA employing dTPT3^Am^TP and SFM4-6

**Fig. 5:**
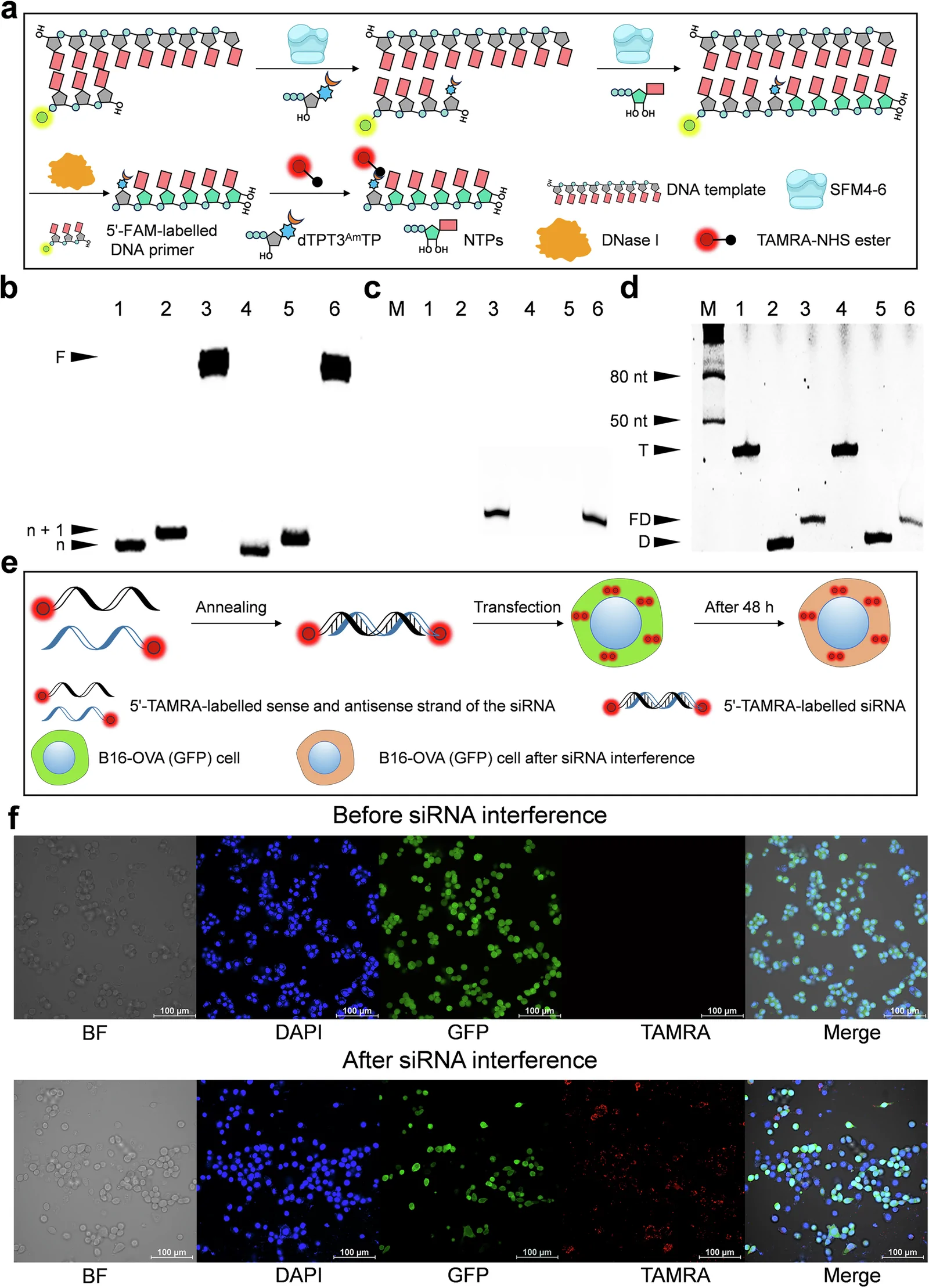
Unnatural base (UB) and SFM4-6-based preparation and functional verification of a fluorescently labelled siRNA. **a** Schematic diagram of the preparation of 5’-end-TAMRA-labelled sense or antisense strand of the siRNA via enzymatic synthesis of a 5’-end-dTPT3^Am^-labelled RNA oligonucleotide by SFM4-6. **b** Gel analysis for the intermediate and final products of the primer extension for the enzymatic synthesis of 5’-end-dTPT3^Am^-labelled sense and antisense strands of the siRNA. Lanes 1 and 4: primer FAM-P-20; lanes 2 and 5: primer FAM-P-20 extended by the incorporation of a dTPT3^Am^ nucleotide using template T42-sense (2) or T42-anti (5), respectively; lanes 3 and 6: product of primer extension by the synthesis of the sense strand (3) or antisense strand (6) RNA, respectively, after the incorporation of a dTPT3^Am^ nucleotide. n: position for primer FAM-P-20 (20 nt); n + 1: position for primer FAM-P-20 incorporating a dTPT3^Am^ nucleotide (21 nt); F: full-length product of the primer extension. **c**, **d** Degradation of the primer extension products with DNase I, and coupling of the produced 5’-end-dTPT3^Am^-labelled sense and antisense strands with TAMRA-NHS ester. The gel was visualized with an excitation wavelength of 555 nm (**c**) or 488 nm (**d**) before (**c**) and after (**d**) staining with Cyber Gold. Lane M: low range ssRNA ladder; lanes 1 and 4: DNA templates T42-sense (1) and T42-anti (4); lanes 2 and 5: 5’-end-dTPT3^Am^-labelled sense strand (2) and antisense strand (5) produced by DNase I degradation of the primer extension products; lanes 3 and 6: coupling products of the 5’-end-dTPT3^Am^-labelled sense strand (3) and antisense strand (6) with TAMRA-NHS ester. T: DNA template T42-sense/T42-anti (42 nt); D: DNase I degradation product of the primer extension product. FD: Coupling product of the 5’-end-dTPT3^Am^-labelled sense/antisense strand with TAMRA-NHS ester. **e** Schematic diagram for the in vitro siRNA interference experiment with the prepared 5’-end-TAMRA-labelled siRNA. **f** Verification of the interference activity of the 5’-end-TAMRA-labelled siRNA. Up: images of B16-OVA(GFP) cells before siRNA interference; down: images of B16-OVA(GFP) cells after interference with the prepared 5’-end-TAMRA-labelled siRNA. Source data are provided in the Source Data file. Figure created in part with BioRender.com. Mei, J. (2026) https://BioRender.com/v4y9m7u.

To demonstrate the practical application of the above-established method for the synthesis of 5’-end-UB-labelled RNA and 2’-modified RNA oligonucleotides, we next prepared several 5’-end-dTPT3^Am^-labelled functional RNA and 2’-modified RNA oligonucleotides, further labelled them with fluorophore-coupled NHS esters, and verified the function of the produced 5’-end-fluorescent-labelled oligonucleotides. Since 5’-end labelling for both the sense and antisense strands of an siRNA has been previously reported for the construction of siRNA therapeutics^52^, we first prepared a 5’-end-fluorescent-labelled siRNA for GFP and tested its function for the knockdown of GFP expression in B16-OVA(GFP) cells. The 5’-end-dTPT3^Am^-labelled sense and antisense strands of an siRNA for GFP (GFP-siRNA) were synthesized via controlled pause and restart of primer extension as described above (Fig. 5a). Gel analysis of the primer extension products demonstrated the successful synthesis of the 5’-end-dTPT3^Am^-labelled sense and antisense strands (Fig. 5b). After removal of the DNA primer and template by DNase I degradation, the produced pure 5’-end-dTPT3^Am^-labelled sense and antisense strands were coupled with 5(6)-carboxytetramethylrhodamine-N-succinimidyl (TAMRA-NHS) ester, resulting in the efficient production of the 5’-end-TAMRA-labelled sense and antisense strands, as indicated by the result of gel analysis of the coupling products (Fig. 5c, d). The 5’-end-TAMRA-labelled sense and antisense strands were annealed together to produce the 5’-end-TAMRA-labelled GFP-siRNA. To assess the activity of the TAMRA in the produced siRNA, we compared its fluorescence spectrum with that of a commercial 5’-end-TAMRA-labelled RNA oligonucleotide produced by a commercial oligonucleotide, TAMRA-R-21 (Supplementary Table 1), under identical conditions. As shown in Supplementary Fig. 14, the fluorescence spectra of these two molecules are almost identical, suggesting that the activity of the TAMRA was well retained during the preparation of the 5’-end-TAMRA-labelled GFP-siRNA. Then the siRNA was transfected into B16-OVA(GFP) cells, and the cells were subsequently cultured for 48 h (Fig. 5e) and then imaged by confocal fluorescence microscopy. As shown in Fig. 5f, the untransfected B16-OVA(GFP) cells exhibited normal expression of GFP, evidenced by the strong green fluorescence in these cells. The 5’-end-TAMRA-labelled GFP-siRNA efficiently entered the transfected cells, evidenced by the red fluorescence in these cells, and the expression of GFP significantly decreased in these transfected cells, suggesting the efficient interference with the siRNA. Flow cytometry experiments were further carried out with the B16(OVA)GFP cells, interfered with the prepared 5’-end-TAMRA-labelled GFP-siRNA or not, to quantitatively analyse the transfection and interference efficiencies. As shown in Supplementary Fig. 15, the prepared 5’-end-TAMRA-labelled GFP-siRNA efficiently entered more than 98% of the cells and reduced the expression of GFP in more than 95% of the cells. These results clearly suggested that the 5’-end-TAMRA-labelled siRNA prepared with dTPT3^Am^TP and SFM4-6 using the above-established method is functional, and this method should be useful for the production of various 5’-end-labelled functional RNAs.

### Preparation of a 5’-end-fluorescent-labelled and functional 2’-F-CU-RNA aptamer for human α-thrombin employing dTPT3^Am^TP and SFM4-6

**Fig. 6:**
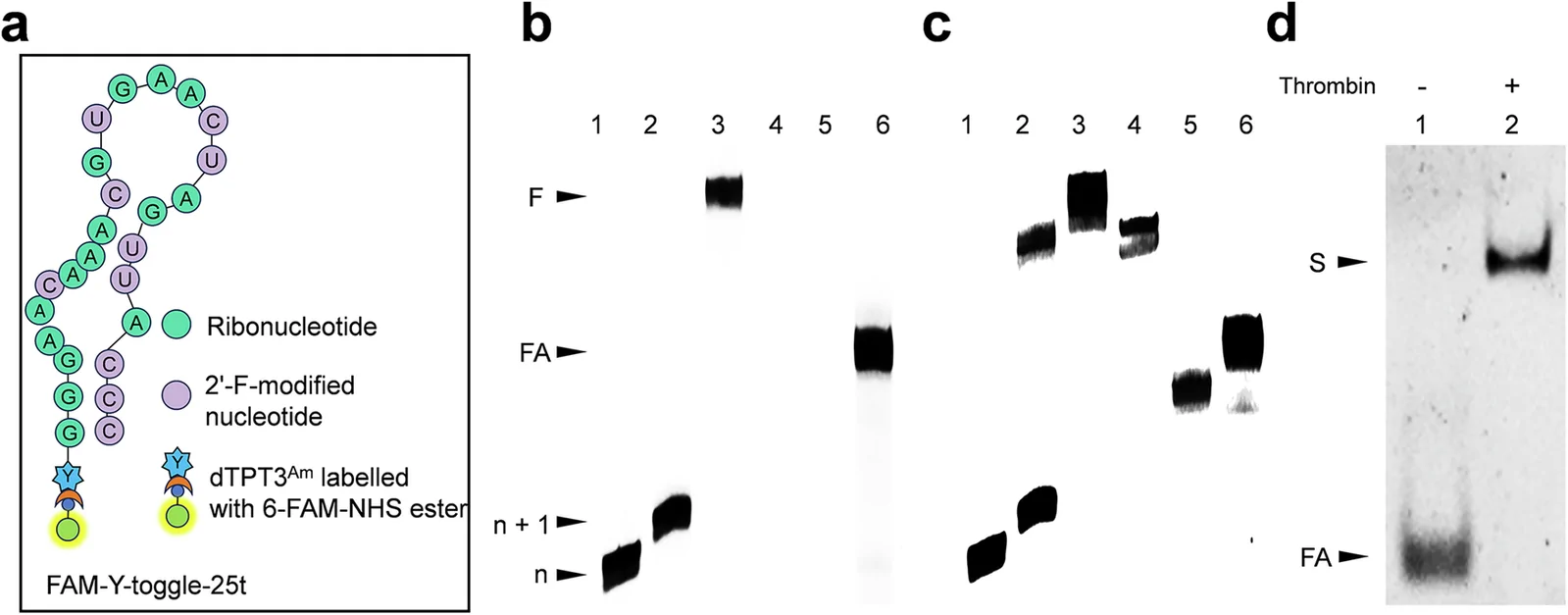
Preparation and binding affinity assay of a 5’-end-FAM-labelled 2’-F-CU-RNA aptamer for human α-thrombin. **a** Secondary structure of 5’-end-FAM-labelled 2’-F-CU-RNA aptamer FAM-Y-toggle-25t predicted with *RNAfold* software. **b**, **c** Gel analysis of the intermediate and final products for the preparation of FAM-Y-toggle-25t. The products were analyzed with a 20% denaturing PAGE gel containing 8 M urea, and the gel was visualized before (**b**) and after (**c**) staining with Cyber Gold. Lane 1: primer FAM-P-20; lane 2: FAM-P-20 extended by the incorporation of a dTPT3^Am^ nucleotide; lane 3: primer extension with ATP, 2’-F-UTP, 2’-F-CTP, and GTP after the incorporation of a dTPT3^Am^ nucleotide; lane 4: DNA template T46-thr; lane 5: product of DNase I degradation of the primer extension product; lane 6: 5’-end-FAM-labelled 2’-F-CU-RNA aptamer FAM-Y-toggle-25t produced by coupling the product of DNase I degradation with 6-FAM-NHS ester. n: position for primer FAM-P-20 (20 nt); n + 1: position for primer FAM-P-20 incorporating a dTPT3^Am^ nucleotide (21 nt); F: full-length product of the primer extension; FA: 5’-end-FAM-labelled 2’-F-CU-RNA aptamer FAM-Y-toggle-25t. **d** Assay for the binding of FAM-Y-toggle-25t with human α-thrombin. The products were analyzed with a 9% PAGE gel, and the gel was directly visualized without staining. FA: the prepared 5’-end-FAM-labelled 2’-F-CU-RNA aptamer FAM-Y-toggle-25t; S: the shifted band for FAM-Y-toggle-25t upon binding with human α-thrombin. Source data are provided in the Source Data file.

Next, we explored the use of the same method for the preparation of a 5’-end-fluorescent-labelled functional partially 2’-modified RNA oligonucleotide. Toggle-25t, which is a 2’-F-CU-RNA aptamer of human α-thrombin^53,54^, was selected as the example oligonucleotide. 5’-End-dTPT3^Am^-labelled 2’-F-CU-RNA aptamer toggle-25t, Y-toggle-25t, was synthesized via controlled pause and restart of primer extension as described above. After removal of the DNA primer and template by degradation with DNase I, the produced pure Y-toggle-25t was coupled with 6-FAM-NHS ester, producing 5’-end-FAM-labelled 2’-F-CU-RNA aptamer FAM-Y-toggle-25t (Fig. 6a). Gel analysis demonstrated the efficient production of all the desired products for different steps (Fig. 6b, c). FAM-Y-toggle-25t was folded and subjected to electrophoretic mobility shift assay (EMSA) for its binding with human α-thrombin. As shown in Fig. 6d, FAM-Y-toggle-25t demonstrated an excellent target binding affinity, evidenced by a significant shift of its band on the gel upon incubation with human α-thrombin. Clearly, the original function of the 2’-F-CU-RNA aptamer was not obviously affected by the 5’-end dTPT3^Am^ coupled with 6-FAM-NHS ester, suggesting the potential of this method for preparing 5’-end-labelled functional partially 2’-modified RNA molecules.

### Preparation of 5’-end-fluorescent-labelled and functional 2’-F/OMe-RNA aptamers for *Bacillus cereus* 5/B/6 metallo-β-lactamase (*B. cereus* 5/B/6 MBL) employing dTPT3^Am^TP and SFM4-6

**Fig. 7:**
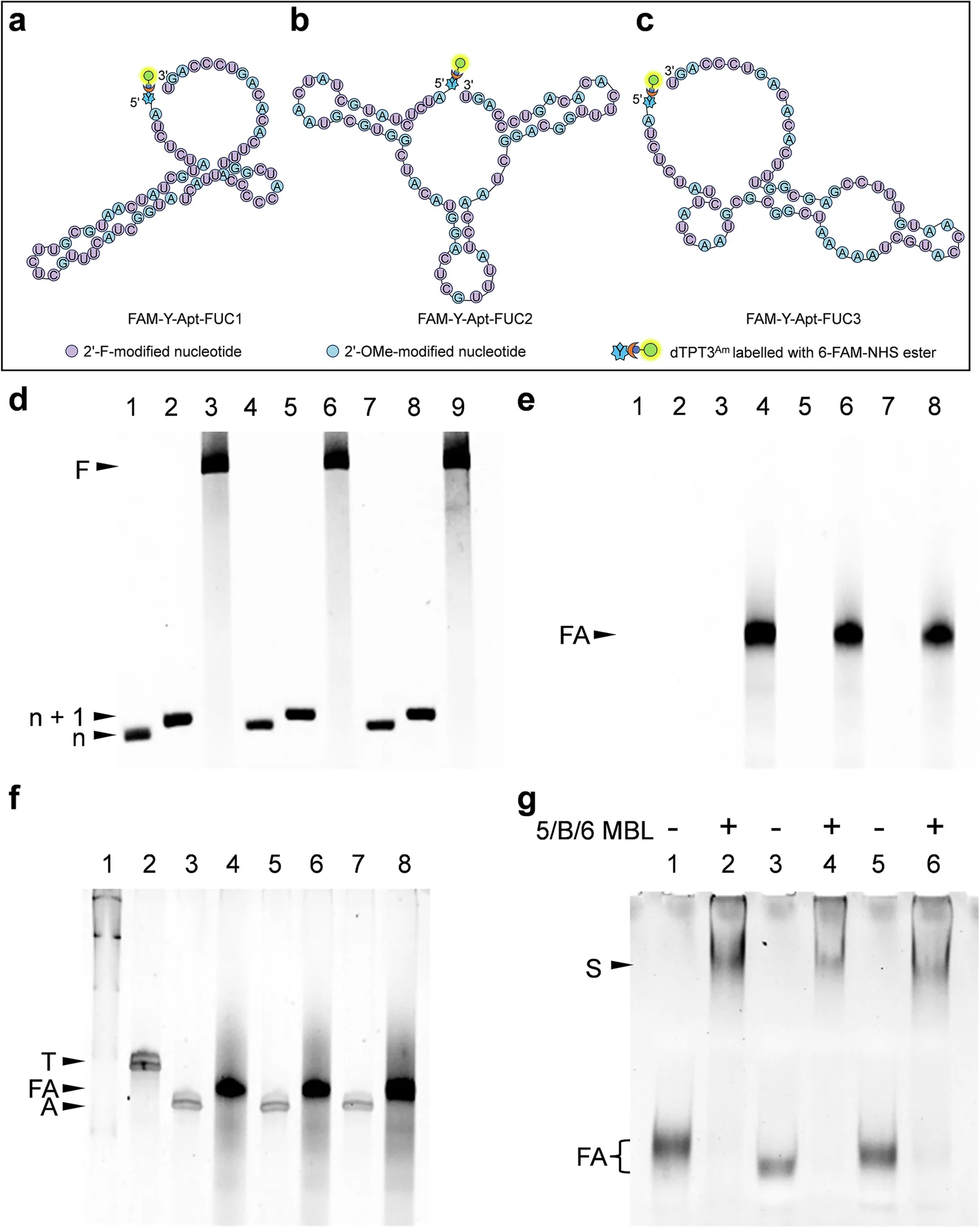
Preparation and functional test of 5’-end-FAM-labelled 2’-F/OMe-RNA aptamers for *B. cereus* 5/B/6 MBL. Secondary structures of 5’-end-FAM-labelled 2’-F/OMe-RNA aptamers FAM-Y-Apt-FUC1 (**a**), FAM-Y-Apt-FUC2 (**b**), and FAM-Y-Apt-FUC3 (**c**) predicted with *RNAfold* software. **d** Gel analysis for the intermediate and final products of primer extension for the preparation of Y-Apt-FUC1, Y-Apt-FUC2, and Y-Apt-FUC3. The gel was directly visualized without staining. Lanes 1, 4, and 7: DNA primer FAM-P-19; lanes 2, 5, and 8: FAM-P-19 extended by the incorporation of a dTPT3^Am^ nucleotide; lanes 3, 6, 9: primer extension with 2’-OMe-ATP, 2’-F-UTP, 2’-F-CTP, and 2’-OMe-GTP after the incorporation of a dTPT3^Am^ nucleotide, for the preparation of Y-Apt-FUC1 (3), Y-Apt-FUC2 (6), and Y-Apt-FUC3 (9). n: position for primer FAM-P-19 (19 nt); n + 1: position for primer FAM-P-19 incorporating a dTPT3^Am^ nucleotide (20 nt); F: full-length product of the primer extension. **e**, **f** Preparation of the 5’-end-FAM-labelled 2’-F/OMe-RNA aptamers. The gel was visualized before (**e**) and after (**f**) staining with Cyber Gold. Lane 1: low range ssRNA marker; lane 2: DNA template T90-1; lanes 3, 5, and 7: 5’-end-dTPT3^Am^-labelled 2’-F/OMe-RNA aptamers Y-Apt-FUC1(3), Y-Apt-FUC2 (5), and Y-Apt-FUC3 (7) produced by DNase I degradation of the primer extension products; lanes 4, 6, and 8: 5’-end-FAM-labelled 2’-F/OMe-RNA aptamers FAM-Y-Apt-FUC1(4), FAM-Y-Apt-FUC2 (6), and FAM-Y-Apt-FUC3 (8) produced by coupling the 5’-end-dTPT3^Am^-labelled 2’-F/OMe-RNA aptamers with 6-FAM-NHS ester. T: DNA template T90-1 (90 nt); A: 5’-end-dTPT3^Am^-labelled 2’-F/OMe-RNA aptamers produced by DNase I degradation; FA: the prepared 5’-end-FAM-labelled 2’-F/OMe-RNA aptamers. **g** Assay for the binding of FAM-Y-Apt-FUC1, FAM-Y-Apt-FUC2, and FAM-Y-Apt-FUC3 with *B. cereus* 5/B/6 MBL. The products were analyzed with a 9% PAGE gel, and the gel was imaged without staining. Lanes 1, 3, and 5: FAM-Y-Apt-FUC1(1), FAM-Y-Apt-FUC2 (3), and FAM-Y-Apt-FUC3 (5) incubated without *B. cereus* 5/B/6 MBL; lanes 2, 4, and 6: FAM-Y-Apt-FUC1 (2), FAM-Y-Apt-FUC2 (4), and FAM-Y-Apt-FUC3 (6) incubated with *B. cereus* 5/B/6 MBL. FA: the prepared 5’-end-FAM-labelled 2’-F/OMe-RNA aptamers; S: the shifted band for 5’-end-FAM-labelled 2’-F/OMe-RNA aptamers upon binding with *B. cereus* 5/B/6 MBL. Source data are provided in the Source Data file.

We then tested the preparation of 5’-end-fluorescent-labelled and functional fully 2’-modified RNA oligonucleotides with the same method. Apt-FUC1, Apt-FUC2, and Apt-FUC3 are three fully 2’-modified aptamers (composed of 2’-F-C, 2’-F-U, 2’-OMe-A, and 2’-OMe-G nucleotides) targeting *B. cereus* 5/B/6 MBL that were selected by systematic evolution of ligands by exponential enrichment (SELEX) technology using SFM4-6 for the preparation of the 2’-modified RNA library in our laboratory^15^ (Fig. 7a–c). Due to their good target binding affinity, we selected them as the example molecules to be prepared with the 5’-end fluorescent labelling and subjected to subsequent functional tests. 5’-End-FAM-labelled 2’-F/OMe-RNA aptamers FAM-Y-Apt-FUC1, FAM-Y-Apt-FUC2, and FAM-Y-Apt-FUC3 were prepared using the same method as FAM-Y-toggle-25t. Gel analysis demonstrated the efficient production of all the desired products for different steps (Fig. 7d–f). The EMSA experiment was then carried out to test the activity of these aptamers for binding with *B. cereus* 5/B/6 MBL. As shown in Fig. 7g, all of these aptamers exhibited high target binding affinities, indicated by significant shifts of their bands on the gel upon incubation with *B. cereus* 5/B/6 MBL. This demonstrates the versatility of this method for the preparation of not only 5’-end-labelled functional RNAs and partially 2’-modified RNAs, but also 5’-end-labelled functional fully 2’-modified nucleic acids.

### Preparation of site-specifically dual-labelled 2’-F-CU-RNA oligonucleotides via controlled pause and restart of SFM4-6-mediated primer extension with a dNaM-containing DNA template and functional d/rTPT3TP derivatives

**Fig. 8:**
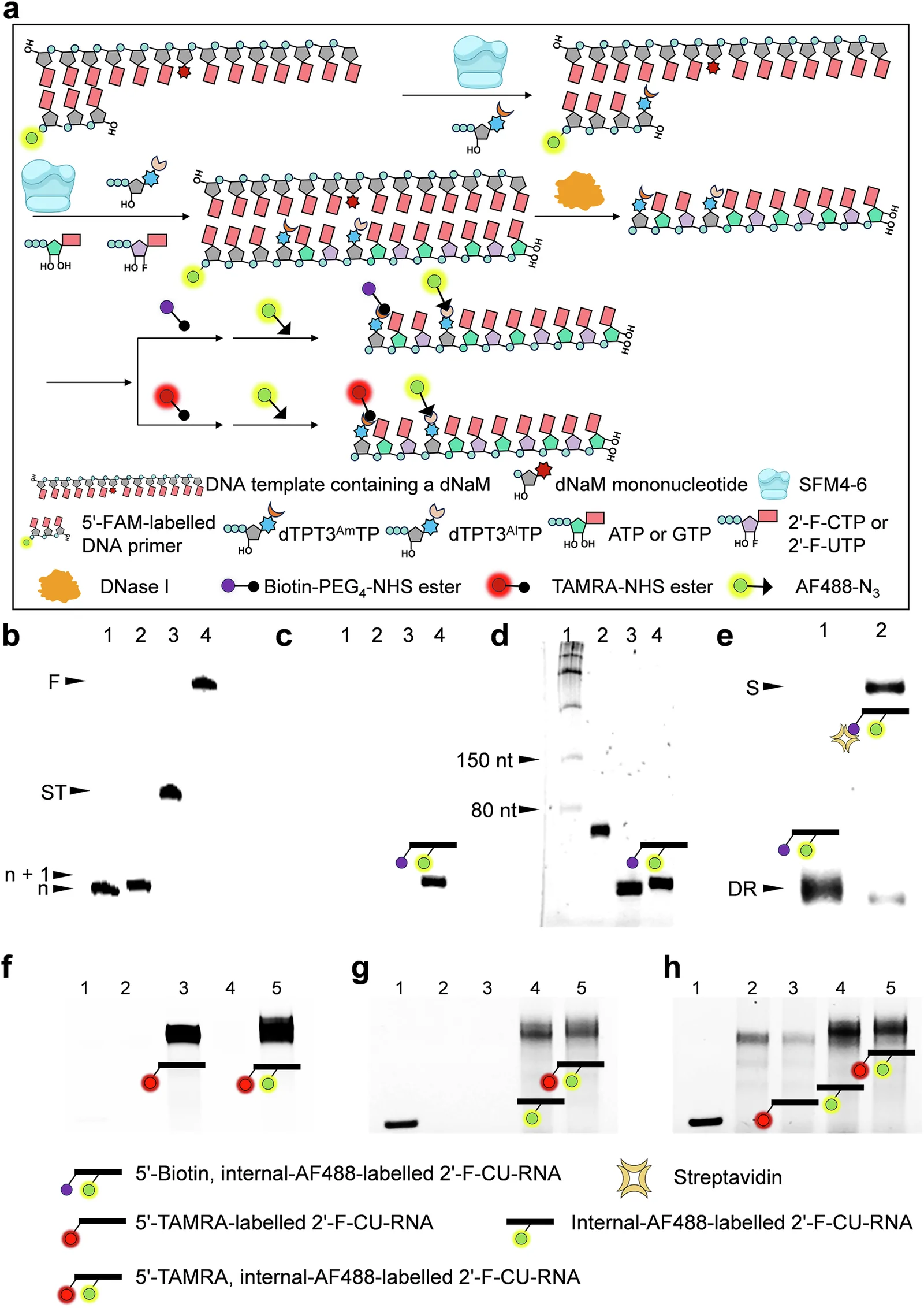
Preparation of dual-labelled 2’-F-CU-RNA oligonucleotides with functional analogues of dTPT3TP mediated by SFM4-6. **a** Schematic diagram. **b** Gel analysis for the products of SFM4-6-mediated primer extension. The gel was unstained. Lane 1: Primer FAM-P-18; lane 2: FAM-P-18 extended by incorporating a dTPT3^Am^ opposite a dA in the DNA template; lane 3: FAM-P-18 extended with ATP, 2’-F-UTP, 2’-F-CTP, and GTP after the incorporation of a dTPT3^Am^; lane 4: FAM-P-18 extended with ATP, 2’-F-UTP, 2’-F-CTP, GTP, and dTPT3^Al^TP after the incorporation of a dTPT3^Am^. n: FAM-P-18 (18 nt); n + 1: FAM-P-18 incorporating a dTPT3^Am^ (19 nt); ST: product of primer extension stalled at the position opposite the dNaM in the template; F: full-length primer extension product. **c**, **d** Gel analysis of the prepared dTPT3^Am^ and dTPT3^Al^-labelled 2’-F-CU-RNA oligonucleotide and AF488 and biotin-labelled 2’-F-CU-RNA oligonucleotide. The gel was visualized before (**c**) and after (**d**) Cyber Gold staining. Lane 1: low-range ssRNA ladder; lane 2: DNA template T55-X; lane 3: dTPT3^Am^ and dTPT3^Al^-labelled 2’-F-CU-RNA oligonucleotide prepared by DNase I degradation of the primer extension product in lane 4 in (**b**); lane 4: AF488 and biotin-labelled 2’-F-CU-RNA oligonucleotide prepared by coupling the oligonucleotide in lane 3 with AF488-N_3_ and biotin-PEG_4_-NHS ester. **e** Biotin gel-shift assay of the AF488 and biotin-labelled 2’-F-CU-RNA oligonucleotide. Lane 1: the AF488 and biotin-labelled 2’-F-CU-RNA oligonucleotide; lane 2: the oligonucleotide in lane 1 incubated with SA. DR: the AF488 and biotin-labelled 2’-F-CU-RNA oligonucleotide. S: the shifted band for the oligonucleotide upon binding with SA. **f**–**h** Gel analysis of the coupling products of the dTPT3^Am^ and dTPT3^Al^-labelled 2’-F-CU-RNA oligonucleotide with fluorescent molecules. The gel was visualized before (**f**, **g**) and after (**h**) Cyber Gold staining with an excitation at 555 nm (**f**) or 488 nm (**g**, **h**). Lane 1: Primer FAM-P-18; lane 2: the dTPT3^Am^ and dTPT3^Al^-labelled 2’-F-CU-RNA oligonucleotide; 3: the dual-labelled 2’-F-CU-RNA oligonucleotide coupled with TAMRA-NHS ester; 4: the dual-labelled 2’-F-CU-RNA oligonucleotide coupled with AF488-N_3_; 5: the dual-labelled 2’-F-CU-RNA oligonucleotide coupled with both TAMRA-NHS ester and AF488-N_3_. Source data are provided in the Source Data file. Figure created in part with BioRender.com. Mei, J. (2026) https://BioRender.com/v4y9m7u.

Site-specific dual labelling is important for some basic research and applications of nucleic acids, such as dynamic investigation of the mechanisms for the functions of nucleic acids via fluorescence resonance energy transfer (FRET) and construction of nucleic acid-based biosensors^39,55^. After establishing the above methods for preparing RNA and 2’-modified RNA oligonucleotides labelled at a specific internal position or the 5’ end, we next attempted to leverage the ability of SFM4-6 to synthesize the UBPs and unnatural-natural mispairs under different conditions to produce RNA or 2’-modified RNA oligonucleotides with dual labelling via unnatural bases. This was achieved by controlled pause and restart of the primer extension using a dNaM-containing DNA template mediated by SFM4-6 (Fig. 8a). To verify the design, FAM-labelled DNA primer FAM-P-18 was extended by the incorporation of a dTPT3^Am^ nucleotide opposite a dC nucleotide in DNA template T55-X. The product was purified, and the primer and template were re-annealed. Then the primer extension was restarted by the addition of ATP, 2’-F-UTP, 2’-F-CTP, and GTP, together with dTPT3^Al^TP or not. The products were analyzed by a 20% denaturing PAGE gel. As shown in Fig. 8b, the primer was efficiently extended by the incorporation of a dTPT3^Am^ nucleotide in the first step. For the restarted primer extension, the primer extension stalled at the position opposite the dNaM nucleotide in the template in the absence of dTPT3^Al^TP, while the primer was efficiently extended to the full length in the presence of dTPT3^Al^TP, suggesting that the dTPT3^Al^ nucleotide was successfully incorporated into the synthesized 2’-F-CU-RNA oligonucleotide. After DNase I treatment to remove the DNA primer and template, the 5’-end-dTPT3^Am^ and internal-dTPT3^Al^-labelled 2’-F-CU-RNA oligonucleotide was coupled with AF488-N_3_ only, both AF488-N_3_ and biotin-PEG_4_-NHS ester, TAMRA-NHS ester only, or both TAMRA-NHS ester and AF488-N_3_, and the products were analyzed by 20% denaturing PAGE gels (Figs. 8c, d, f–h) or a 9% PAGE gel (Fig. 8e). As shown in Fig. 8c–e, a bright and clear band was observed on the gel before the gel was stained with Cyber gold for the dual-labelled 2’-F-CU-RNA oligonucleotide coupled with both AF488-N_3_ and biotin-PEG_4_-NHS ester, suggesting the successful incorporation of the internal dTPT3^Al^ nucleotide and its efficient labelling by AF488-N_3_. Meanwhile, an aliquot of the dual-labelled 2’-F-CU-RNA oligonucleotide coupled with both AF488-N_3_ and biotin-PEG_4_-NHS ester was incubated with SA, and the product was analyzed with a 9% PAGE gel. As shown in Fig. 8e, a significant shift of the band for this oligonucleotide was observed upon its incubation with SA, suggesting the efficient incorporation and biotin labelling of the 5’-end dTPT3^Am^ nucleotide. Figure 8f–h show the gel analysis results of the coupling products of the dual-labelled oligonucleotide with TAMRA-NHS ester, AF488-N_3_, or both. When the gel was imaged with an excitation wavelength of 555 nm, fluorescent bands were observed for each of the products coupled with TAMRA-NHS ester (Fig. 8f). When the gel was imaged with an excitation wavelength of 488 nm, fluorescent bands were observed for each of the products coupled with AF488-N_3_ (Fig. 8g). After the gel was stained with Cyber gold and when the gel was imaged with an excitation wavelength of 488 nm, fluorescent bands were observed for all the products (Fig. 8h). These results again suggest the efficient incorporation and labelling of the 5’-end dTPT3^Am^ and internal dTPT3^Al^ nucleotides, and together with other results shown above, demonstrate the potential of the established method in the production of dual-labelled or dual-functionalized RNA and 2’-modified RNA oligonucleotides.

### Preparation of 3’-end-UB-labelled RNA and 2’-F/OMe-RNA oligonucleotides by SFM4-6-mediated incorporation of a dTPT3 or rTPT3 nucleotide opposite a natural nucleotide at the 5’ end of the DNA template

**Fig. 9:**
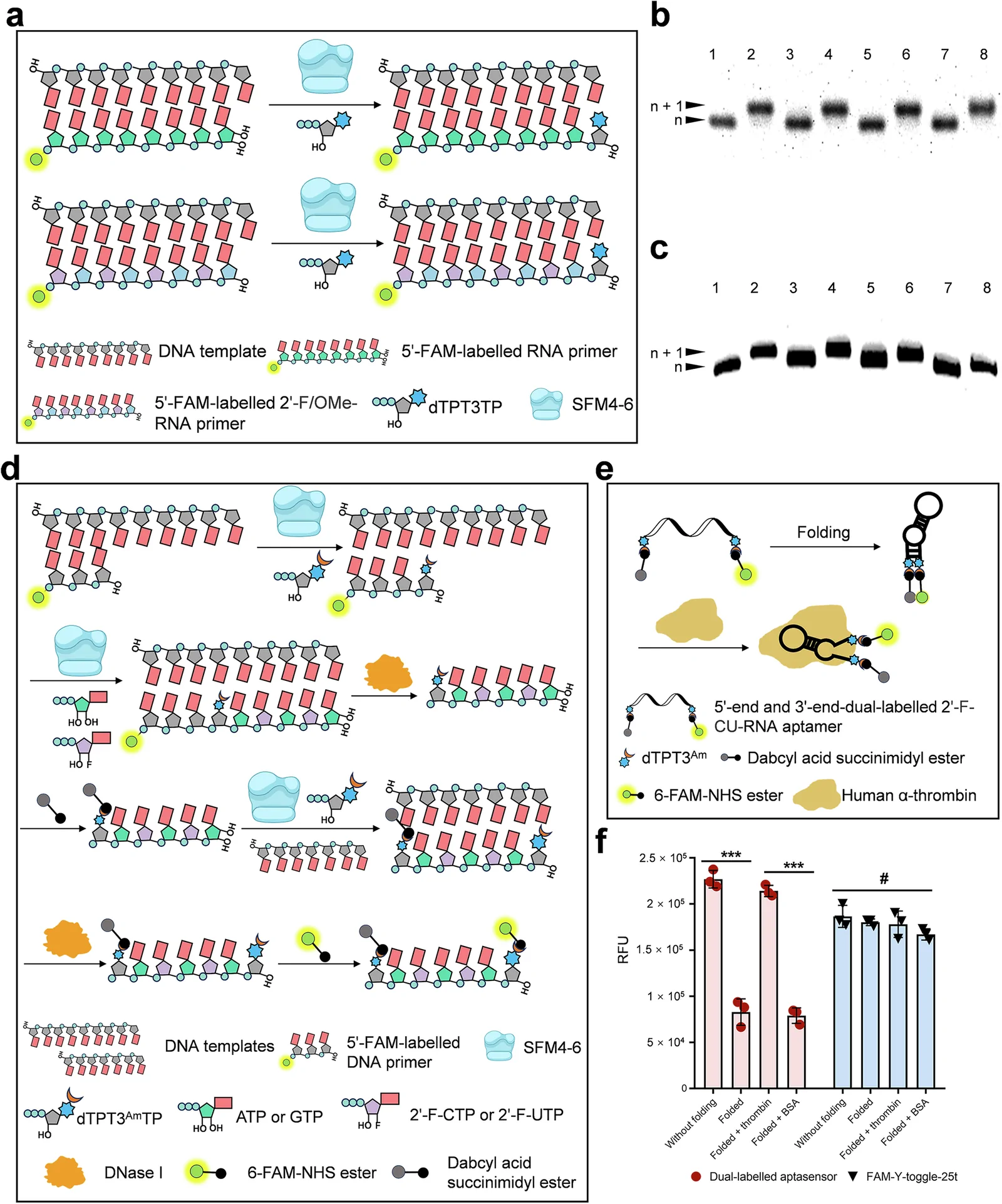
Preparation of 5’-end and 3’-end-dual-labelled RNA, 2’-F/OMe-RNA, and 2’-F-CU-RNA oligonucleotides and its application in the construction of a 2’-F-CU-RNA aptasensor. **a** Schematic diagram for the extension of RNA and 2’-F/OMe-RNA oligonucleotides by SFM4-6-mediated incorporation of dTPT3TP opposite a natural nucleotide at the 5’ end of the template. **b**, **c** Gel analysis for the products of the extension of RNA (**b**) and 2’-F/OMe-RNA (**c**) oligonucleotides. The products were analyzed with a 20% denaturing PAGE gel, and the gel was directly visualized without staining. Lanes 1, 3, 5, and 7: 5’-end-FAM-labelled RNA oligonucleotide FAM-R-25 (**b**) or 2’-F/OMe-RNA oligonucleotide FAM-f/m-25 (**c**); lanes 2, 4, 6, and 8: 5’-end-FAM-labelled RNA (**b**) or 2’-F/OMe-RNA (**c**) oligonucleotide extended by the incorporation of dTPT3TP opposite a dA (2), dT (4), dC (6), or dG (8) nucleotide with DNA template T26-A (2), T26-T (4), T26-C (6), or T26-G (8). n: position for oligonucleotide FAM-R-25 (25 nt) (**b**) or FAM-f/m-25 (25 nt) (**c**). n + 1: position for FAM-R-25 (**b**) or FAM-f/m-25 (**c**) extended by the incorporation of a dTPT3 nucleotide (26 nt). **d** Schematic diagram for the preparation of a 5’-end-Dabcyl and 3’-end-FAM-dual-labelled 2’-F-CU-RNA aptamer that can be used as a 2’-F-CU-RNA aptasensor for human α-thrombin. **e** Schematic diagram for the detection of human α-thrombin with the prepared 2’-F-CU-RNA aptasensor. **f** Functional test of the prepared 2’-F-CU-RNA aptasensor (pink) and 5’-end-FAM-labelled 2’-F-CU-RNA aptamer FAM-Y-toggle-25t (blue) for the detection of human α-thrombin. The fluorescence was read for the oligonucleotide without folding, the folded oligonucleotide, the folded oligonucleotide incubated with human α-thrombin, and the folded oligonucleotide incubated with BSA. Error bars indicate standard deviations from the means of the reads from three independent biological replicates (*n* = 3); statistical analysis was performed using an unpaired two-tailed *t*-test; ***P = 0.0001 for Folded versus without folding; ***P < 0.0001 for Folded + BSA versus Folded + thrombin; #: non-significance. Source data are provided in the Source Data file. Figure created in part with BioRender.com. Mei, J. (2026) https://BioRender.com/v4y9m7u.

Other than the dual-labelled 2’-modified RNA oligonucleotides described above, we also explored the preparation of 3’-end-UB-labelled, or 5’-end-FAM and 3’-end-UB-dual-labelled RNA and 2’-modified RNA oligonucleotides employing SFM4-6. 5’-End-FAM-labelled RNA or 2’-F/OMe-RNA primer FAM-R-25 or FAM-f/m-25 was annealed to a DNA template, which is complementary to the primer and has an additional dA, dT, dC, or dG nucleotide at the 5’ end, and extended via the incorporation of dTPT3TP or rTPT3TP opposite the 5’-end nucleotide of the template by SFM4-6 (Fig. 9a). As shown in Fig. 9b and c, with the optimized reaction time and concentration of dTPT3TP or rTPT3TP, for all cases, primer FAM-R-25 or FAM-f/m-25 was completely extended by the incorporation of a dTPT3 or rTPT3 nucleotide, demonstrating the successful and efficient preparation of 3’-end-UB-labelled, or 5’-end-FAM and 3’-end-UB-dual-labelled RNA and 2’-modified RNA oligonucleotides.

### Preparation of a functional 5’-end and 3’-end-dual-labelled 2’-F-CU-RNA aptasensor for human α-thrombin employing dTPT3^Am^TP and SFM4-6

To demonstrate the practical use of the above-established methods in the preparation of 5’-end and 3’-end-dual-labelled functional RNA or 2’-modified RNA oligonucleotides, we next prepared a 5’-end and 3’-end-dual-labelled 2’-F-CU-RNA aptasensor for human α-thrombin and tested its function for the target detection (Fig. 9d, e). First, 5’-end-dTPT3^Am^-labelled human α-thrombin 2’-F-CU-RNA aptamer Y-toggle-25t was prepared as described above, and then coupled with 4-[4-(dimethylamino)phenylazo]benzoic acid N-succinimidyl ester (Dabcyl acid SE, a small molecule quencher for the fluorescence of FAM) to produce 5’-end-Dabcyl-labelled aptamer Dabcyl-Y-toggle-25t (Fig. 9d, Supplementary Fig. 16). Dabcyl-Y-toggle-25t was further labelled at the 3’ end via the incorporation of a dTPT3^Am^ nucleotide by SFM4-6, and then coupled with 6-FAM-NHS ester to produce 5’-end-Dabcyl and 3’-end-FAM-dual-labelled 2’-F-CU-RNA aptamer Dabcyl-Y-toggle-25t-Y-FAM. 10 nM of folded Dabcyl-Y-toggle-25t-Y-FAM was mixed and incubated with 100 nM human α-thrombin or bovine serum albumin (BSA, control). The fluorescence of Dabcyl-Y-toggle-25t-Y-FAM without folding, folded Dabcyl-Y-toggle-25t-Y-FAM, and folded Dabcyl-Y-toggle-25t-Y-FAM incubated with human α-thrombin or BSA was read with a plate reader. As shown in Fig. 9f, the folding of Dabcyl-Y-toggle-25t-Y-FAM led to a significant decrease in the fluorescence, which could be attributed to a closer distance between the FAM and Dabcyl groups in the folded aptamer. Remarkably, incubation of the folded Dabcyl-Y-toggle-25t-Y-FAM with human α-thrombin led to a recovery of the fluorescence to over 94% of the original fluorescence, likely due to a conformational change of the aptamer that increased the distance between the FAM and Dabcyl groups upon the target binding. In sharp contrast, incubation of the folded Dabcyl-Y-toggle-25t-Y-FAM with BSA did not lead to an obvious change in the fluorescence, suggesting the good target specificity of the aptasensor. In parallel, a group of control experiments were also carried out by replacing Dabcyl-Y-toggle-25t-Y-FAM with a 5’-end-FAM-labelled aptamer for human α-thrombin, FAM-Y-toggle-25t, under identical conditions. No obvious difference in the fluorescence was observed among FAM-Y-toggle-25t without folding, folded FAM-Y-toggle-25t, folded FAM-Y-toggle-25t incubated with human α-thrombin, and folded FAM-Y-toggle-25t incubated with BSA (Fig. 9f), suggesting that both the fluorophore (FAM) and the quencher (Dabcyl) are necessary for the function of the aptasensor. These results clearly suggest the successful preparation of a 5’-end and 3’-end-dual-labelled 2’-F-CU-RNA aptasensor for human α-thrombin with our methods.

To conclude, in this work, we investigated and characterized the activity of a polymerase engineered for the efficient synthesis of 2’-modified nucleic acids, SFM4-6, for the synthesis of UBPs dNaM-dTPT3, dNaM-rTPT3, and their functionalized analogues. As revealed by the experimental results, SFM4-6 is capable of synthesizing these UBPs with good efficiency and specificity. Based on this, RNA and 2’-modified RNA oligonucleotides site-specifically incorporating a nucleotide of dTPT3, rTPT3, or one of their functionalized analogues were successfully synthesized with SFM4-6, allowing the production of site-specifically labelled or functionalized RNA and 2’-modified RNA oligonucleotides. Next, we explored and demonstrated the capability of SFM4-6 for the synthesis of unnatural-natural mispairs. Based on this, we established a method for the synthesis of 5’-end-UB-labelled RNA and 2’-modified RNA oligonucleotides via controlled pause and restart of primer extension with a natural DNA template and SFM4-6. To demonstrate the value of this method in practical application, we used this method to prepare several 5’-end-labelled functional RNA and 2’-modified RNA oligonucleotides, including an siRNA and several aptamers, and successfully verified their functions. Clearly, this method is efficient and versatile for the production of various 5’-end-labelled functional RNA and 2’-modified RNA oligonucleotides that will retain the original functions while possessing additional functions. Moreover, taking advantage of the varied activities of SFM4-6 for the synthesis of the UBPs and unnatural–natural mispairs, a method was developed for the preparation of site-specifically dual-labelled RNA and 2’-modified RNA oligonucleotides. Finally, we established a method for the preparation of 3’-end-UB-labelled RNA and 2’-modified RNA oligonucleotides by SFM4-6-mediated incorporation of a dTPT3 or rTPT3 nucleotide opposite a natural nucleotide at the 5’ end of the DNA template. To demonstrate the application of this method, we combined it with the method for the synthesis of 5’-end-UB-labelled RNA and 2’-modified RNA oligonucleotides to prepare a 5’-end-Dabcyl and 3’-end-FAM-labelled 2’-F-CU-RNA aptasensor for human α-thrombin, whose function was subsequently verified. In summary, this work not only demonstrates the capability of an engineered 2’-modified nucleic acid polymerase for the synthesis of a panel of UBPs, which enables the synthesis of nucleic acid molecules possessing both an unnatural sugar-phosphate backbone and the unnatural bases, but also provides a series of methods for the facile and efficient synthesis of RNA and 2’-modified RNA oligonucleotides site-specifically single or dual-labelled or functionalized at various sites.

## Methods

### Materials

Unnatural nucleoside triphosphates dNaMTP, rNaMTP, dTPT3TP, rTPT3TP, dTPT3^Am^TP, dTPT3^Al^TP, and rTPT3^Al^TP were purchased from WuXi AppTec (Wuxi, China). 2’-Fluoro-NTPs (2’-F-ATP, 2’-F-UTP, 2’-F-CTP, and 2’-F-GTP) and 2’-methoxy-NTPs (2’-OMe-ATP, 2’-OMe-UTP, 2’-OMe-CTP, and 2’-OMe-GTP) were purchased from Huaren Science & Technology Co., Ltd. (Wuhu, China). DNase I (RNase-free), 10× ThermoPol reaction buffer, and 5× OneTaq reaction buffer were purchased from New England Biolabs (Ipswich, MA, USA). Zymo ssDNA/RNA Clean & Concentrator^TM^ kit was purchased from Zymo Research (Irvine, CA, USA). Isopropyl-β-D-thiogalactoside (IPTG) and magnesium chloride (MgCl_2_) were purchased from Solarbio Science & Technology Co., Ltd. (Beijing, China). Cyber gold was purchased from Biolite Biotech Co., Ltd. (Xi’an, China). Tris(3-hydroxypropyltriazolylmethyl) amine (THPTA) was purchased from Xi’an Confluore Biological Technology Co., Ltd. (Xi’an, China). 6× Loading buffer was purchased from Beijing Genesand Biotech Co., Ltd. (Beijing, China). 6-Carboxyfluorescein N-succinimidyl (6-FAM-NHS) ester, 5(6)-carboxytetramethylrhodamine succinimidyl (TAMRA-NHS) ester, Alexa Fluor488 azide (AF488-N_3_), biotin-PEG_4_-NHS ester, and biotin-PEG_3_-azide (biotin-PEG_3_-N_3_) were purchased from Vector Laboratories Inc. (Newark, CA, USA). 4-[4-(Ddimethylamino)phenylazo]benzoic acid N-succinimidyl ester (Dabcyl acid SE) was purchased from Heowns Biochem Technologies, LLC, Tianjin (Tianjin, China). BCA protein assay kit and diethyl pyrocarbonate (DEPC)-treated water were purchased from Beyotime Biotechnology Co., Ltd. (Shanghai, China). 5’-End-6-carboxyfluorescein (FAM)-labelled RNA and 2’-F/OMe-RNA primers FAM-R-25 and FAM-f/m-25, 5’-end-6-carboxyfluorescein (FAM)-labelled DNA primers FAM-P-18, FAM-P-19, and FAM-P-20, DNA templates T55-X, T55-A, T55-T, T55-C, T55-G, T46-thr, T42-sense, T42-anti, T90-1, T90-2, T90-3, T26-A, T26-T, T26-C, and T26-G (Supplementary Table 1), low molecular weight DNA ladder, low range ssRNA ladder, and 2× TBE-urea sample buffer were purchased from Sangon Biotech Co., Ltd. (Shanghai, China). All FAM-labelled DNA primers, DNA templates containing dNaM, and RNA primers were purified by HPLC (purity ≥ 98%), and the other primers and templates were purified by PAGE (purity ≥ 98%). *E. coli* BL21(DE3)/pLysS cells harboring plasmid pET23b (+)-SFM4-6 were constructed and stored in our laboratory^15^. Murine melanoma cell line B16-OVA(GFP) was a gift from Zhu group from School of Biology and Biological Engineering, South China University of Technology.

### Ethics statement

No human participants, clinical samples, or live vertebrate animals were involved in this study. Therefore, ethical approval from an ethics committee and institution was not required for any of the experimental protocols described herein.

### Expression and purification of SFM4-6

SFM4-6 was expressed in *E. coli* BL21(DE3)/pLysS cells harboring plasmid pET23b (+)-SFM4-6. A single colony was inoculated into 2× YT medium supplemented with 100 μg/mL ampicillin and 25 μg/mL chloramphenicol, and the culture was incubated with shaking at 37 °C overnight. The overnight culture was re-inoculated at a ratio of 1:100 into fresh 2× YT medium supplemented with 100 μg/mL ampicillin and 25 μg/mL chloramphenicol and incubated with shaking at 37 °C. When OD_600_ of the culture reached 0.6–0.8, 0.4 mM IPTG was added to induce protein expression, and the culture was then incubated with shaking at 25 °C overnight. The cells were then harvested by centrifugation at 4629×*g* and 4 °C, resuspended in 1× buffer A (50 mM Tris–HCl, 300 mM NaCl, 10 mM imidazole, pH = 8.0), and disrupted by high-pressure homogenisation. The cell lysate was incubated at 70 °C for 20 min to precipitate most of the cellular proteins, which were then removed by centrifugation at 12,857×*g* and 4 °C for 40 min. The filtered supernatant was loaded onto a Ni-NTA sefinose resin 6FF column. The column was washed with 10 column volumes of 1× buffer A, and then the protein was eluted with elution buffer (50 mM Tris–HCl, 50–500 mM imidazole, 300 mM NaCl, pH = 8.0). The purified protein (in elution fractions of 100 and 200 mM imidazole) was then concentrated with an Amicon Ultra-15 centrifugal filter (50 kDa) and washed six times with dialysis buffer (50 mM Tris–HCl, 150 mM NaCl, 2 mM DTT, 0.1% tween 20, pH = 7.5). After concentration to a volume <500 µL, the purified SFM4-6 protein was quantified using a BCA protein assay kit and stored in 50% glycerol at −20 °C.

### Primer extension assay for the incorporation of different nucleoside triphosphates opposite a dNaM nucleotide in the DNA template by SFM4-6

To prepare the reaction mixture for primer extension, dNaM-containing DNA template T55-X and FAM-labelled DNA primer FAM-P-20 were annealed in ThermoPol reaction buffer and OneTaq standard reaction buffer by incubating at 95 °C for 10 min, slowly cooling down to room temperature, and incubating on ice for 10 min. Then 0.1 µM FAM-P-20/0.15 µM T55-X was mixed with 0.1 µM nucleoside triphosphate (dATP, dTTP, dCTP, dGTP, ATP, UTP, CTP, GTP, 2’-F-ATP, 2’-F-UTP, 2’-F-CTP, 2’-F-GTP, 2’-OMe-ATP, 2’-OMe-ATP, 2’-OMe-UTP, 2’-OMe-CTP, 2’-OMe-GTP, dNaMTP, rNaMTP, dTPT3TP, rTPT3TP, dTPT3^Am^TP, dTPT3^Al^TP, or rTPT3^Al^TP), 5 mM MgCl_2_, and 1 nM SFM4-6 in a mixture of 1× ThermoPol reaction buffer and 1× OneTaq standard reaction buffer, and the primer extension was carried out by incubating the mixture at 50 °C for 20 s. The reaction was quenched by the addition of 2× TBE-urea sample buffer and incubated at 95 °C for 10 min. All the products were analyzed with 20% denaturing PAGE gels containing 8 M urea.

### Steady-state kinetic experiments for SFM4-6-mediated incorporation of natural and unnatural nucleoside triphosphates opposite a natural or unnatural nucleotide in the DNA template

Steady-state kinetic experiments were first carried out for SFM4-6-mediated incorporation of dTPT3TP, rTPT3TP, dNaMTP, and rNaMTP opposite a dNaM nucleotide in the DNA template. DNA template T55-X and FAM-labelled DNA primer FAM-P-20 were annealed in ThermoPol reaction buffer and OneTaq standard reaction buffer as described above. Then 50 nM FAM-P-20/100 nM T55-X was mixed with varying concentrations of dTPT3TP, rTPT3TP, dNaMTP, or rNaMTP, 5 mM MgCl_2_, and 1 nM (for dTPT3TP or rTPT3TP) or 6 nM (for dNaMTP or rNaMTP) SFM4-6 in a mixture of 1× ThermoPol reaction buffer and 1× OneTaq standard reaction buffer. The reactions were incubated at 50 °C for 20 s (for dTPT3TP or rTPT3TP) or 2 min (for dNaMTP or rNaMTP), quenched by the addition of 2× TBE-urea sample buffer, and incubated at 95 °C for 10 min. To compare the kinetic constants for SFM4-6-mediated incorporation of nucleoside triphosphates containing unnatural and natural bases opposite their complementary nucleotides in the DNA template, steady-state kinetic experiments were also carried out for SFM4-6-mediated incorporation of dTTP and UTP opposite a dA nucleotide in the DNA template. DNA template T55-A and primer FAM-P-20 were annealed in ThermoPol reaction buffer and OneTaq standard reaction buffer as described above. Then 50 nM FAM-P-20/100 nM T55-A was mixed with varying concentrations of dTTP or UTP, 5 mM MgCl_2_, and 1 nM SFM4-6 in a mixture of 1× ThermoPol reaction buffer and 1× OneTaq standard reaction buffer. The reactions were incubated at 50 °C for 20 s, quenched by the addition of 2× TBE-urea sample buffer, and incubated at 95 °C for 10 min. All the products were analyzed with 20% denaturing PAGE gels containing 8 M urea, and the bands on the gels were quantified using Quantity One analysis software (Bio-Rad). *K*_m_ and *k*_cat_ values were calculated by fitting the data to the Michaelis–Menten equation with GraphPad Prism 8 software. All experiments were performed with three independent biological replicates.

### Primer extension assay for SFM4-6-mediated synthesis of RNA and 2’-modified RNA oligonucleotides incorporating a dTPT3, rTPT3, dTPT3^Am^, dTPT3^Al^, or rTPT3^Al^ nucleotide at a specific internal position using a dNaM-containing DNA template

To prepare the reaction mixture for primer extension, dNaM-containing DNA template T55-X and FAM-labelled DNA primer FAM-P-18 were annealed in ThermoPol reaction buffer and OneTaq standard reaction buffer as described above. Then 0.1 µM FAM-P-18/0.15 µM T55-X was mixed with 5 mM MgCl_2_, 0.6 mM each of NTPs; ATP, 2’-F-UTP, 2’-F-CTP, and GTP; 2’-OMe-ATP, 2’-F-UTP, 2’-F-CTP, and 2’-OMe-GTP; or 2’-OMe-NTPs, 0 or 10 µM dTPT3TP, rTPT3TP, dTPT3^Am^TP, dTPT3^Al^TP, or rTPT3^Al^TP, 0 or 1 mM MnCl_2_, and 40 nM or 200 nM SFM4-6 in a mixture of 1× ThermoPol reaction buffer and 1× OneTaq standard reaction buffer. For primer extension with NTPs, or ATP, 2’-F-UTP, 2’-F-CTP, and GTP, 40 nM SFM4-6 was used, and the reaction was incubated at 50 °C for 30 min. For primer extension with 2’-OMe-ATP, 2’-F-UTP, 2’-F-CTP, and 2’-OMe-GTP; or 2’-OMe-NTPs, 1 mM MnCl_2_ and 200 nM SFM4-6 was used, and the reaction was incubated at 50 °C for 4 h or carried out with the following thermocycling programme: 6 cycles of (50 °C, 2 h; 70 °C, 30 min); 50 °C, 2 h^13,15^. The reaction was quenched by the addition of 2× TBE-urea sample buffer and incubated at 95 °C for 10 min. All the products were analyzed with 20% denaturing PAGE gels containing 8 M urea.

### Fluorescent labelling assay of the RNA, 2’-F-CU-RNA, 2’-F/OMe-RNA, and 2’-OMe-RNA oligonucleotides incorporating a dTPT3^Am^, dTPT3^Al^, or rTPT3^Al^ nucleotide at a specific internal position prepared by SFM4-6-mediated primer extension

DNA template T55-X and FAM-labelled DNA primer FAM-P-18 were annealed in ThermoPol reaction buffer and OneTaq standard reaction buffer as described above. Then 0.1 µM FAM-P-18/0.15 µM T55-X was mixed with 5 mM MgCl_2_, 1 µM dTPT3^Am^TP, dTPT3^Al^TP, or rTPT3^Al^TP, 0.4 mM each of NTPs; ATP, 2’-F-UTP, 2’-F-CTP, and GTP; 2’-OMe-ATP, 2’-F-UTP, 2’-F-CTP, and 2’-OMe-GTP; or 2’-OMe-NTPs, 0 mM MnCl_2_ and 40 nM SFM4-6 (for primer extension with NTPs; or ATP, 2’-F-UTP, 2’-F-CTP, and GTP) or 1 mM MnCl_2_ and 200 nM SFM4-6 (for primer extension with 2’-OMe-ATP, 2’-F-UTP, 2’-F-CTP, and 2’-OMe-GTP; or 2’-OMe-NTPs) in a mixture of 1× ThermoPol reaction buffer and 1× OneTaq standard reaction buffer. For primer extension with NTPs; or ATP, 2’-F-UTP, 2’-F-CTP, and GTP, the reaction was incubated 50 °C for 30 min. For primer extension with 2’-OMe-ATP, 2’-F-UTP, 2’-F-CTP, and 2’-OMe-GTP, the reaction was incubated at 50 °C for 4 h. For primer extension with 2’-OMe-NTPs, the reaction was conducted with the following thermocycling programme: 6 cycles of (50 °C, 2 h; 70 °C, 30 min); 50 °C, 2 h. The product of primer extension was purified and concentrated with a Zymo ssDNA/RNA Clean & Concentrator^TM^ kit. Then the DNA primer and template were degraded by incubating the purified product with 0.5 U/µL DNase I (RNase-free) in 1× DNase I reaction buffer at 37 °C for 16 h, and the DNase I degradation product was purified and concentrated with a Zymo ssDNA/RNA Clean & Concentrator^TM^ kit. The produced dTPT3^Am^-containing oligonucleotides were mixed with 1 mM 6-FAM-NHS ester in 1× PBS buffer (pH = 8.5) and incubated 37 °C for 2 h. The produced dTPT3^Al^ or rTPT3^Al^-containing oligonucleotides were mixed with 100 μM CuSO_4_, 500 μΜ THPTA, 100 μM AF488-N_3_, and 2.5 mM ascorbic acid in 1× PBS buffer (pH = 7.4) and incubated at 50 °C for 30 min. All the reaction products were purified and concentrated with a Zymo ssDNA/RNA Clean & Concentrator^TM^ kit, and analyzed with 20% denaturing PAGE gels containing 8 M urea. The gels were visualized using a gel imager before and after Cyber gold staining.

### Primer extension assay for SFM4-6 mediated incorporation of dTPT3TP or rTPT3TP opposite a natural nucleotide in the template

DNA template T55-A, T55-T, T55-C, or T55-G and FAM-labelled DNA primer FAM-P-20 were annealed in ThermoPol reaction buffer and OneTaq standard reaction buffer as described above. 50 nM FAM-P-20/100 nM T55-A, T55-T, T55-C, or T55-G was mixed with 5 mM MgCl_2_, 12.5–400 nM dTPT3TP or rTPT3TP, and 3 nM (for dTPT3TP) or 6 nM (for rTPT3TP) SFM4-6 in a mixture of 1× ThermoPol reaction buffer and 1× OneTaq standard reaction buffer and incubated at 50 °C for 8 min (for T55-A), 10 min (for T55-T), 15 min (for T55-C), or 12 min (for T55-G). The reaction was quenched by the addition of 2× TBE-urea sample buffer and incubated at 95 °C for 10 min. All products were analyzed with 20% denaturing PAGE gels containing 8 M urea.

### Steady-state kinetic experiments for SFM4-6-mediated incorporation of dTPT3TP or rTPT3TP opposite a natural nucleotide in the template

DNA template T55-A, T55-T, T55-C, or T55-G and FAM-labelled DNA primer FAM-P-20 were annealed in ThermoPol reaction buffer and OneTaq standard reaction buffer as described above. 50 nM FAM-P-20/100 nM T55-A, T55-T, T55-C, or T55-G was mixed with 5 mM MgCl_2_, 0–64 μM dTPT3TP or rTPT3TP, and 6 nM SFM4-6 in a mixture of 1× ThermoPol reaction buffer and 1× OneTaq standard reaction buffer and incubated at 50 °C for 2 min. The reaction was quenched by the addition of 2× TBE-urea sample buffer and incubated at 95 °C for 10 min. All products were analyzed with 20% denaturing PAGE gels containing 8 M urea. The bands on the gels were quantified using Quantity One analysis software. The *K*_m_ and *k*_cat_ values were calculated by fitting the data to the Michaelis-Menten equation with GraphPad Prism 8 software. All experiments were performed with three independent biological replicates.

### Steady-state kinetic experiments for SFM4-6-mediated incorporation of ATP, 2’-F-ATP, 2’-OMe-ATP, and dTPT3TP opposite a dT nucleotide at the *n* + 2 position in the DNA template after the incorporation of a dTPT3 nucleotide opposite a dA nucleotide at the *n* + 1 position

DNA template T55-A and FAM-labelled DNA primer FAM-P-20 were annealed in ThermoPol reaction buffer and OneTaq standard reaction buffer as described above. 50 nM FAM-P-20/100 nM T55-A was mixed with 5 mM MgCl_2_, 0.2 μM dTPT3TP, and 6 nM SFM4-6 in a mixture of 1× ThermoPol reaction buffer and 1× OneTaq standard reaction buffer and incubated at 50 °C for 8 min. Then the primer/template was purified and concentrated with a Zymo ssDNA/RNA Clean & Concentrator^TM^ kit and quantified with Nano Drop 2000 (Thermo Fisher Scientific). The template and primer were annealed as described above again. Then 50 nM primer/template was mixed with 5 mM MgCl_2_, 0-8 μM ATP, 2’-F-ATP, or 2’-OMe-ATP or 0–64 μM dTPT3TP, and 1 nM (for ATP, 2’-F-ATP, or 2’-OMe-ATP) or 6 nM (for dTPT3TP) SFM4-6 in a mixture of 1× ThermoPol reaction buffer and 1× OneTaq standard reaction buffer and incubated at 50 °C for 20 s (for ATP or 2’-F-ATP) or 2 min (for 2’-OMe-ATP or dTPT3TP). The reaction was quenched by the addition of 2× TBE-urea sample buffer and incubated at 95 °C for 10 min. All the products were analyzed with 20% denaturing PAGE gels containing 8 M urea. The bands on the gels were quantified using Quantity One analysis software. *K*_m_ and *k*_cat_ values were calculated by fitting the data to the Michaelis–Menten equation with GraphPad Prism 8 software. All experiments were performed with three independent biological replicates.

### Measurement of the time course of SFM4-6-mediated incorporation of dTPT3TP opposite a dA nucleotide in the DNA template

FAM-labelled DNA primer FAM-P-20 and DNA template T55-A were annealed in ThermoPol reaction buffer and OneTaq standard reaction buffer as described above. 50 nM FAM-P-20/100 nM T55-A was mixed with 5 mM MgCl_2_, 64 μM dTPT3TP, and 6 nM SFM4-6 in a mixture of 1× ThermoPol reaction buffer and 1× OneTaq standard reaction buffer and incubated at 50 °C for 0, 10, 20, 40, 60, 80, 120, 160, 200, 240, 300, or 360 s. The reaction was quenched by the addition of 2× TBE-urea sample buffer and incubated at 95 °C for 10 min. The products were analyzed with 20% denaturing PAGE gels containing 8 M urea. The bands on the gels were quantified using Quantity One analysis software. The curves for the time course of primer extension were obtained by plotting the extension percentages of the primer at different time points using GraphPad Prism 8 software. Linear fitting of the data was carried out for the extension percentages of the primer in the range of 0–120 s, and the *R*² value was calculated. All experiments were performed with three independent biological replicates.

### SFM4-6-mediated primer extension with controlled pause and restart for the enzymatic synthesis of 5’-end-dTPT3 or rTPT3-labelled RNA, 2’-F-CU-RNA, 2’-F/OMe-RNA, and 2’-OMe-RNA oligonucleotides

DNA template T55-A and FAM-labelled DNA primer FAM-P-20 were annealed in ThermoPol reaction buffer and OneTaq standard reaction buffer as described above. 50 nM FAM-P-20/100 nM T55-A was mixed with 5 mM MgCl_2_, 0.2 μM dTPT3TP or rTPT3TP, and 3 nM (for dTPT3TP) or 6 nM (for rTPT3TP) SFM4-6 in a mixture of 1× ThermoPol reaction buffer and 1× OneTaq standard reaction buffer and incubated at 50 °C for 8 min. After that, 0.4 mM each of NTPs; ATP, 2’-F-UTP, 2’-F-CTP, and GTP; 2’-OMe-ATP, 2’-F-UTP, 2’-F-CTP, and 2’-OMe-GTP; or 2’-OMe-NTPs, and 1 mM MnCl_2_ (only for primer extension with 2’-OMe-ATP, 2’-F-UTP, 2’-F-CTP, and 2’-OMe-GTP; or 2’-OMe-NTPs) was directly added into the reaction solution to restart the primer extension. For the restarted primer extension with NTPs; or ATP, 2’-F-UTP, 2’-F-CTP, and GTP, the reaction was incubated at 50 °C for 30 min. For the restarted primer extension with 2’-OMe-ATP, 2’-F-UTP, 2’-F-CTP, and 2’-OMe-GTP; or 2’-OMe-NTPs, the reaction was carried out with the following thermocycling programme: 6 cycles of (50 °C, 2 h; 70 °C, 30 min); 50 °C, 2 h^13,15^. The reaction was quenched by the addition of 2× TBE-urea sample buffer and incubated at 95 °C for 10 min. All the products were analyzed with 20% denaturing PAGE gels containing 8 M urea.

### Enzymatic synthesis of 5’-end-dTPT3^Am^, dTPT3^Al^, or rTPT3^Al^-labelled RNA, 2’-F-CU-RNA, 2’-F/OMe-RNA, and 2’-OMe-RNA oligonucleotides by SFM4-6-mediated primer extension with controlled pause and restart, and fluorescent and biotin labelling of the produced oligonucleotides

To prepare 5’-end-dTPT3^Am^, dTPT3^Al^, or rTPT3^Al^-labelled RNA, 2’-F-CU-RNA, 2’-F/OMe-RNA, and 2’-OMe-RNA oligonucleotides, DNA template T55-A and primer FAM-P-20 were annealed in ThermoPol reaction buffer and OneTaq standard reaction buffer as described above. Then 0.4 μM FAM-P-20/0.6 μM T55-A was mixed with 5 mM MgCl_2_, 1 μM dTPT3^Am^TP, dTPT3^Al^TP, or rTPT3^AI^TP, and 50 nM SFM4-6 in a mixture of 1× ThermoPol reaction buffer and 1× OneTaq standard reaction buffer and incubated at 50 °C for 8 min to incorporate a dTPT3^Am^, dTPT3^Al^, or rTPT3^Al^ nucleotide opposite a dA nucleotide in the DNA template. Then 0.6 mM each of NTPs; ATP, 2’-F-UTP, 2’-F-CTP, and GTP; 2’-OMe-ATP, 2’-F-UTP, 2’-F-CTP, and 2’-OMe-GTP; or 2’-OMe-NTPs, and 1 mM MnCl_2_ (only for primer extension with 2’-OMe-ATP, 2’-F-UTP, 2’-F-CTP, and 2’-OMe-GTP; or 2’-OMe-NTPs) were directly added into the reaction mixture to restart the primer extension. For the restarted primer extension with NTPs; or ATP, 2’-F-UTP, 2’-F-CTP, and GTP, the reaction was incubated at 50 °C for 30 min. For the restarted primer extension with 2’-OMe-ATP, 2’-F-UTP, 2’-F-CTP, and 2’-OMe-GTP, the reaction was incubated at 50 °C for 4 h. For the restarted primer extension with 2’-OMe-NTPs, the reaction was carried out with the following thermocycling programme: 6 cycles of (50 °C, 2 h; 70 °C, 30 min); 50 °C, 2 h. The primer extension product was purified and concentrated with a Zymo ssDNA/RNA Clean & Concentrator^TM^ kit, and the DNA primer and template were removed by incubating the purified product with 0.5 U/µL DNase I (RNase-free) in 1× DNase I reaction buffer at 37 °C for 16 h. The DNase I degradation product was purified and concentrated with a Zymo ssDNA/RNA Clean & Concentrator^TM^ kit. Then the 5’-end-dTPT3^Am^-labelled oligonucleotide products were mixed with 1 mM 6-FAM-NHS ester or 1 mM biotin-PEG_4_-NHS ester in 1× PBS buffer (pH = 8.5) and incubated at 37 °C for 2 h to label them with biotin or FAM. The 5’-end-dTPT3^Al^ or rTPT3^Al^-labelled oligonucleotide products were mixed with 100 μM CuSO_4_, 500 μΜ THPTA, 100 μM AF488-N_3_ or 100 μM biotin-PEG_3_-N_3_, and 2.5 mM ascorbic acid in 1× PBS buffer (pH = 7.4) and incubated at 50 °C for 30 min to label them with AF488 or biotin. All the reaction products were purified and concentrated with a Zymo ssDNA/RNA Clean & Concentrator^TM^ kit. The FAM or AF488-labelled products were directly analyzed with 20% denaturing PAGE gels containing 8 M urea, and the gels were visualized with a gel imager before and after Cyber gold staining. For the biotin-labelled products, 1 µM of each product was, respectively, incubated with or without 0.1 mg/mL streptavidin (SA) at 37 °C for 2 h, and then all the products were analyzed with 6% PAGE gels. The gels were stained with Cyber gold and visualized with a gel imager.

### Mass spectrometry analysis of the enzymatically synthesized internally site-specifically labelled 2’-modified RNA oligonucleotides and 5’-end-labelled RNA and 2’-modified RNA oligonucleotides

2’-F/OMe-RNA and 2’-OMe-RNA oligonucleotides internally and site-specifically incorporating a nucleotide of rTPT3^Al^ were prepared using the following thermocycling programme for primer extension: 6 cycles of (50 °C, 2 h; 70 °C, 30 min); 50 °C, 2 h. 5’-End-dTPT3^Am^-labelled RNA, 5’-end-dTPT3^Al^-labelled RNA, 5’-end-rTPT3^Al^-labelled RNA, 5’-end-dTPT3^Am^-labelled 2’-F-CU-RNA, 5’-end-dTPT3^Al^-labelled 2’-F-CU-RNA, 5’-end-rTPT3^Al^-labelled 2’-F-CU-RNA, 5’-end-dTPT3^Am^-labelled 2’-F/OMe-RNA, 5’-end-dTPT3^Al^-labelled 2’-F/OMe-RNA, 5’-end-rTPT3^Al^-labelled 2’-F/OMe-RNA, 5’-end-dTPT3^Am^-labelled 2’-OMe-RNA, 5’-end-dTPT3^Al^-labelled 2’-OMe-RNA, and 5’-end-rTPT3^Al^-labelled 2’-OMe-RNA oligonucleotides were prepared as described above, followed by purification and concentration using a Zymo ssDNA/RNA Clean & Concentrator^TM^ kit. Then the purified products were incubated with 0.5 U/µL DNase I (RNase-free) in 1× DNase I reaction buffer at 37 °C for 16 h to remove the DNA primers and templates. The degradation products were analyzed with 20% denaturing PAGE gels containing 8 M urea. The target product bands were excised from the gel, and the excised gel slices were immersed in an appropriate volume of DEPC-treated water, followed by incubation at 50 °C for 12 h. After centrifugation to remove the gel slices, the target products were purified and concentrated using a Zymo ssDNA/RNA Clean & Concentrator^TM^ kit, and sent to Sangon Biotech Co., Ltd. (Shanghai, China) for mass spectrometry analysis. Mass spectrometric analysis was performed using an LTQ XL linear ion trap mass spectrometer system (Thermo Fisher Scientific, San Jose, CA, USA). Mass spectra were acquired over an *m/z* range of 15–4000, and the measured molecular masses were compared with the calculated values to confirm the identities of the synthesized oligonucleotides.

### Preparation of the 5’-end-TAMRA-labelled siRNA

To prepare the 5’-end-TAMRA-labelled sense and antisense strands of an siRNA for green fluorescent protein (GFP), GFP-siRNA, DNA template T42-sense or T42-anti and primer FAM-P-20 were annealed in ThermoPol reaction buffer and OneTaq standard reaction buffer as described above. 0.4 μM FAM-P-20/0.6 μM T42-sense or T42-anti was mixed with 5 mM MgCl_2_, 1 μM dTPT3^Am^TP, and 50 nM SFM4-6 in a mixture of 1× ThermoPol reaction buffer and 1× OneTaq standard reaction buffer and incubated at 50 °C for 10 or 12 min, respectively, to extend the primer by incorporating a dTPT3^Am^ opposite a dT or dG nucleotide in the template. Then 0.6 mM each of NTPs was directly added into the reaction mixture to restart the primer extension, and the reaction was incubated at 50 °C for 30 min. The primer extension products were analyzed by a 20% denaturing PAGE gel, and the gel was imaged with a gel imager without staining. For each of the sense and antisense strands, the primer extension product was purified and concentrated with a Zymo ssDNA/RNA Clean & Concentrator^TM^ kit, and the DNA primer and template were removed by incubating the purified product with 0.5 U/µL DNase I (RNase-free) in 1× DNase I reaction buffer at 37 °C for 16 h. The DNase I degradation product was purified and concentrated with a Zymo ssDNA/RNA Clean & Concentrator^TM^ kit. Then the 5’-end-dTPT3^Am^-labelled sense and antisense strands of GFP-siRNA were annealed in diethyl pyrocarbonate (DEPC) water by incubating at 70 °C for 5 min, slowly cooling down to room temperature, and incubating on ice for 10 min. The annealing product was mixed with 1 mM TAMRA-NHS ester in 1× PBS buffer (pH = 8.5) and incubated at 37 °C for 2 h. The produced 5’-end-TAMRA-labelled GFP-siRNA was purified and concentrated with a Zymo ssDNA/RNA Clean & Concentrator^TM^ kit and quantified with Nano Drop 2000. The TAMRA-labelled products were analyzed with a 20% denaturing PAGE gel containing 8 M urea, and the gel was imaged using a gel imager with an excitation wavelength of 555 nm before staining with Cyber gold and with an excitation wavelength of 488 nm after Cyber gold staining. The fluorescence spectrum for the prepared 5’-end-TAMRA-labelled GFP-siRNA was acquired with a microplate reader (Molecular Devices SpectraMax ID3, excitation wavelength: 250 nm; emission wavelength: 290–750 nm) and compared with that of a commercial 5’-end-TAMRA-labelled RNA oligonucleotide produced by chemical synthesis, TAMRA-R-21 (Supplementary Table 1).

### In vitro siRNA interference experiments

A GFP-expressing murine melanoma cell line B16-OVA(GFP) was used to test the cellular uptake of and gene silencing mediated by the 5’-end-TAMRA-labelled GFP-siRNA. B16-OVA(GFP) cells were maintained in cell growth medium [Roswell Park Memorial Institute 1640 (RPMI-1640) supplemented with 10% foetal bovine serum (FBS) and 1% penicillin–streptomycin]. Three days before the cellular uptake and gene silencing experiments, the cells were seeded in two ⌀15 mm glass-bottom dishes at a density of 10,000 cells/dish and cultured at 37 °C in 5% CO_2_. After 24 h, 1 µg of the 5’-end-TAMRA-labelled GFP-siRNA and 5 μL PEI transfection reagents were, respectively, added into 100 µL phenol red-free Opti-MEM^TM^, mixed, and incubated at room temperature for 30 min to produce a solution of siRNA–PEI complex. After removal of the cell growth media, the cells in the culture dishes were washed three times with 1× PBS buffer (pH = 7.4). The solution of siRNA–PEI complex (200 µL) was added into one culture dish with the cells, and 200 µL of phenol red-free Opti-MEM^TM^ was added into the other for a control experiment. Then the cells were incubated at 37 °C for 4 h. After removal of the supernatant, 1 mL of cell growth medium was added to each culture dish, and the cells were cultured at 37 °C in 5% CO_2_. After 48 h, the medium was removed, and the cells were washed three times with 1× PBS buffer (pH = 7.4), stained with Hoechst 33342 (Beyotime), and washed three times again with 1× PBS buffer (pH = 7.4). Then the cells were imaged with an ultra-high-resolution laser confocal microscope (Nikon AX N-STORM).

To quantitatively evaluate the interference efficiency of the prepared 5’-end-TAMRA-labelled GFP-siRNA on GFP expression in B16-OVA (GFP) cells, flow cytometry experiments were carried out to analyse the cells interfered with the 5’-end-TAMRA-labelled GFP-siRNA or not. Cells were seeded in six-well plates (50,000 cells/well) and cultured for 24 h prior to transfection. The siRNA–PEI complex was prepared by mixing 5 µg of the 5’-end-TAMRA-labelled GFP-siRNA with 25 µL PEI in 500 µL phenol red-free Opti-MEM^TM^ and incubating the mixture at room temperature for 30 min. siRNA–PEI complex was added to the cells prewashed with 1× PBS buffer (pH = 7.4), and then the cells were incubated at 37 °C for 4 h. After removal of the supernatant, 2 mL of cell growth medium was added to each well, and the cells were cultured at 37 °C in 5% CO_2_. After 48 h, the medium was discarded, and the cells were washed three times with 1× PBS (pH = 7.4). Then the cells in each well were detached by the addition of 500 µL of trypsin-EDTA (0.25%) solution and incubation at 37 °C for 2 min, and the digestion was quenched with 1 mL of growth medium. The cell suspension was centrifuged at 200×*g* and room temperature for 3 min, and the cell pellet was washed six times with 1× PBS (pH = 7.4) to remove the residual siRNA-PEI complex. The cells were filtered through a 40 µm mesh and analyzed using a flow cytometer (ThermoFisher Bigfoot). For the flow cytometry analysis, a minimum of 10,000 single-cell events were collected for each sample, and data analysis was performed using FlowJo software (v10.8.1). The following gating strategy was applied: intact cells were first identified based on the forward scatter area (FSC-A) and the side scatter area (SSC-A) to exclude cell debris. Single cells were then identified by analyzing the side scatter height (SSC-H) and the side scatter area (SSC-A). The specific gating strategy was shown in the Source Data file. Fluorescence gates for GFP (for the analysis of interference on GFP expression) and TAMRA (for the analysis of siRNA uptake) channels were established based on the signal of blank control cells (treated with only phenol red-free Opti-MEM^TM^) to define the negative and positive cell populations.

### Preparation and target binding assay of the 5’-end-FAM-labelled 2’-F-CU-RNA aptamer for human α-thrombin

To prepare 5’-end-FAM-labelled 2’-F-CU-RNA aptamer toggle-25t for human α-thrombin, FAM-Y-toggle-25t, DNA template T46-thr and primer FAM-P-20 were annealed in ThermoPol reaction buffer and OneTaq standard reaction buffer as described above. 0.4 μM FAM-P-20/0.6 μM T46-thr was mixed with 5 mM MgCl_2_, 1 μM dTPT3^Am^TP, and 50 nM SFM4-6 in a mixture of 1× ThermoPol reaction buffer and 1× OneTaq standard reaction buffer and incubated at 50 °C for 8 min. Then, 0.6 mM each of ATP, 2’-F-UTP, 2’-F-CTP, and GTP was directly added into the reaction mixture to restart the primer extension. The reaction was incubated at 50 °C for 30 min, and then the primer extension product was purified and concentrated with a Zymo ssDNA/RNA Clean & Concentrator^TM^ kit. The DNA primer and template were removed by incubating the purified product with 0.5 U/µL DNase I (RNase-free) in 1× DNase I reaction buffer at 37 °C for 16 h, and the DNase I degradation product was purified and concentrated with a Zymo ssDNA/RNA Clean & Concentrator^TM^ kit. The 5’-end-dTPT3^Am^-labelled 2’-F-CU-RNA aptamer was mixed with 1 mM 6-FAM-NHS ester in 1× PBS buffer (pH = 8.5) and incubated at 37 °C for 2 h. All intermediate and final products were analyzed with a 20% denaturing PAGE gel, and the gel was imaged before and after staining with Cyber gold. The produced 5’-end-FAM-labelled 2’-F-CU-RNA aptamer (FAM-Y-toggle-25t) was purified and concentrated with a Zymo ssDNA/RNA Clean & Concentrator^TM^ kit and quantified with Nano Drop 2000. For the target binding assay, 0.1 μM FAM-Y-toggle-25t was folded in 1× binding buffer (20 mM HEPES, 150 mM NaCl, 6 mM KCl, 2 mM MgCl_2_, pH = 7.5) by incubating at 95 °C for 10 min, slowly cooling down to room temperature, and then incubating on ice for 10 min. Then 10 μM thrombin was added into the solution of the folded aptamer, and the mixture was incubated at 37 °C for 2 h. The product was analyzed with a 9% PAGE gel, and the gel was visualized using a gel imager without staining.

### Preparation and target binding assay of 5’-end-FAM-labelled 2’-F/OMe-RNA aptamers for *Bacillus cereus* 5/B/6 metallo-β-lactamase (*B. cereus* 5/B/6 MBL)

To prepare 5’-end-FAM-labelled 2’-F/OMe-RNA aptamers Apt-FUC1, Apt-FUC2, and Apt-FUC3 for *B. cereus* 5/B/6 MBL, which were obtained via a SELEX experiment in our laboratory in a previous work^15^, FAM-Y-Apt-FUC1, FAM-Y-Apt-FUC2, and FAM-Y-Apt-FUC3, DNA template T90-1, T90-2, or T90-3 and primer FAM-P-19 were annealed in ThermoPol reaction buffer and OneTaq standard reaction buffer as described above. 0.4 μM FAM-P-19/0.6 μM T90-1, T90-2, or T90-3 was mixed with 5 mM MgCl_2_, 1 μM dTPT3^Am^TP, and 50 nM SFM4-6 in a mixture of 1× ThermoPol reaction buffer and 1× OneTaq standard reaction buffer and incubated at 50 °C for 15 min. Then 1 mM MnCl_2_ and 0.4 mM each of 2’-OMe-ATP, 2’-F-UTP, 2’-F-CTP, and 2’-OMe-GTP were added to restart the primer extension. The reaction was conducted with the following thermocycling programme: 6 cycles of (50 °C, 2 h; 70 °C, 30 min); 50 °C, 2 h. All primer extension products were analyzed with a 20% denaturing PAGE gel, and the gel was imaged without staining. The primer extension products were purified and concentrated with a Zymo ssDNA/RNA Clean & Concentrator^TM^ kit, and the DNA primer and template were removed by incubating the purified product with 0.5 U/µL DNase I (RNase-free) in 1× DNase I reaction buffer at 37 °C for 16 h. The DNase I degradation product was purified and concentrated with a Zymo ssDNA/RNA Clean & Concentrator^TM^ kit, and the 5’-end-dTPT3^Am^-labelled 2’-F/OMe-RNA aptamers were, respectively, mixed with 1 mM 6-FAM-NHS ester in 1× PBS buffer (pH = 8.5) and incubated at 37 °C for 2 h. All intermediate and final products were analyzed with a 20% denaturing PAGE gel, and the gel was imaged before and after staining with Cyber gold. The produced 5’-end-FAM-labelled 2’-F/OMe-RNA aptamers were purified and concentrated with a Zymo ssDNA/RNA Clean & Concentrator^TM^ kit and quantified with Nano Drop 2000. For the target binding assay for each aptamer, 0.1 μM 5’-end-FAM-labelled 2’-F/OMe-RNA aptamer was folded in 1× *B. cereus* 5/B/6 MBL binding buffer (20 mM HEPES, 150 mM NaCl, 6 mM KCl, 2 mM MgCl_2_, 1 mM ZnSO_4_, pH = 7.5) by incubating at 95 °C for 10 min, slowly cooling down to room temperature, and then incubating on ice for 10 min. Then 10 μM *B. cereus* 5/B/6 MBL was added into the solution of the folded aptamer, and the mixture was incubated at 37 °C for 2 h. The products were analyzed with a 9% PAGE gel containing 0.1 mM ZnSO_4_. During the gel running process, the temperature of the gel was maintained below 30 °C using an ice box. The gel was visualized with a gel imager without staining.

### Preparation of site-specifically dual-labelled 2’-F-CU-RNA oligonucleotides via controlled pause and restart of SFM4-6-mediated primer extension with a dNaM-containing DNA template and functional d/rTPT3TP derivatives

DNA template T55-X, which contains a dNaM nucleotide, and primer FAM-P-18 were annealed in ThermoPol reaction buffer and OneTaq standard reaction buffer as described above. 0.4 µM FAM-P-18/0.6 µM T55-X was mixed with 5 mM MgCl_2_, 1 µM dTPT3^Am^TP, and 6 nM SFM4-6 in a mixture of 1× ThermoPol reaction buffer and 1× OneTaq standard reaction buffer and incubated at 50 °C for 15 min. The primer extension products were purified and concentrated with a Zymo ssDNA/RNA Clean & Concentrator^TM^ kit and quantified with Nano Drop 2000. The template and primer were annealed again as described above. Then 0.4 µM primer/template was mixed with 5 mM MgCl_2_, 0.4 mM each of ATP, 2’-F-UTP, 2’-F-CTP, and GTP, 0 or 10 µM dTPT3^Al^TP, and 6 nM SFM4-6 in a mixture of 1× ThermoPol reaction buffer and 1× OneTaq standard reaction buffer to restart the primer extension. The reaction was incubated at 50 °C for 30 min. All intermediate and final products of the primer extension were analyzed with a 20% denaturing PAGE gel, and the gel was directly visualized without staining. The primer extension product was purified and concentrated with a Zymo ssDNA/RNA Clean & Concentrator^TM^ kit, and the DNA primer and template were removed by incubating the purified product with 0.5 U/µL DNase I (RNase-free) in 1× DNase I reaction buffer at 37 °C for 16 h. The DNase I degradation product was purified and concentrated with a Zymo ssDNA/RNA Clean & Concentrator^TM^ kit to produce the dTPT3^Am^ and dTPT3^Al^-labelled 2’-F-CU-RNA oligonucleotide. The dTPT3^Am^ and dTPT3^Al^-labelled 2’-F-CU-RNA oligonucleotide was first mixed with 1 mM biotin-PEG_4_-NHS ester or 1 mM TAMRA-NHS ester in 1× PBS buffer (pH = 8.5) and incubated at 37 °C for 2 h. The product was purified with a Zymo ssDNA/RNA Clean & Concentrator^TM^ kit, mixed with 100 μM CuSO_4_, 500 μΜ THPTA, 100 μM AF488-N_3_, and 2.5 mM ascorbic acid in 1× PBS buffer (pH = 7.4), and incubated at 50 °C for 30 min to produce the biotin and AF488-labelled 2’-F-CU-RNA oligonucleotide, or TAMRA and AF488-labelled 2’-F-CU-RNA oligonucleotide. Besides, two aliquots of dTPT3^Am^ and dTPT3^Al^-labelled 2’-F-CU-RNA oligonucleotide were reacted with only TAMRA-NHS ester or AF488-N_3_, respectively, to produce the TAMRA-labelled 2’-F-CU-RNA oligonucleotide and AF488-labelled 2’-F-CU-RNA oligonucleotide. All the products were purified with a Zymo ssDNA/RNA Clean & Concentrator^TM^ kit. The biotin and AF488-labelled 2’-F-CU-RNA oligonucleotide was mixed with 0.1 mg/mL SA or not, and incubated at 37 °C for 2 h. The products were analyzed with a 9% PAGE gel, and the gel was visualized with a gel imager without staining. All other labelling products were analyzed with 20% denaturing PAGE gels containing 8 M urea, and the gels were visualized using a gel imager with an excitation wavelength of 488 or 555 nm before staining with Cyber gold and with an excitation wavelength of 488 nm after staining with Cyber gold.

### SFM4-6-mediated extension of RNA and 2’-F/OMe-RNA primers by incorporating a dTPT3 or rTPT3 nucleotide opposite a natural nucleotide at the 5’ end of the DNA template

DNA template T26-A, T26-T, T26-C, or T26-G and FAM-labelled RNA primer FAM-R-25 or FAM-labelled 2’-F/OMe-RNA primer FAM-f/m-25 were annealed in ThermoPol reaction buffer and OneTaq standard reaction buffer as described above. 0.4 µM FAM-R-25 or FAM-f/m-25/0.6 µM T26-A, T26-T, T26-C, or T26-G was mixed with 5 mM MgCl_2_, 10 µM dTPT3TP or rTPT3TP, and 0.5 µM SFM4-6 in a mixture of 1× ThermoPol reaction buffer and 1× OneTaq standard reaction buffer and incubated at 50 °C for 2 h. The reaction was quenched by the addition of 2× TBE-urea sample buffer and incubated at 95 °C for 10 min. All the products were analyzed with 20% denaturing PAGE gels containing 8 M urea, and the gels were imaged using a gel imager without staining.

### Preparation of the 5’-end and 3’-end-dual-labelled 2’-F-CU-RNA aptasensor for human α-thrombin and its performance test

The oligonucleotide of 5’-end-dTPT3^Am^-labelled 2’-F-CU-RNA aptamer for human α-thrombin, Y-toggle-25t, was prepared with DNA template T46-thr, FAM-labelled DNA primer FAM-P-20, dTPT3^Am^TP, and SFM4-6 as described above. Y-toggle-25t was first mixed with 1 mM 4-[4-(dimethylamino)phenylazo]benzoic acid N-succinimidyl ester (Dabcyl acid SE) in 1× PBS buffer (pH = 8.5) and incubated at 37 °C for 2 h. The produced 5’-end-Dabcyl-labelled 2’-F-CU-RNA aptamer (Dabcyl-Y-toggle-25t) was purified and concentrated with a Zymo ssDNA/RNA Clean & Concentrator^TM^ kit and quantified with Nano Drop 2000. Then DNA template T26-A and Dabcyl-Y-toggle-25t were annealed in ThermoPol reaction buffer and OneTaq standard reaction buffer as described above. 0.4 µM Dabcyl-Y-toggle-25t/T26-A was mixed with 5 mM MgCl_2_, 10 µM dTPT3^Am^TP, and 0.5 µM SFM4-6 in a mixture of 1× ThermoPol reaction buffer and 1× OneTaq standard reaction buffer and incubated at 50 °C for 12 h. The extension product was purified and concentrated with a Zymo ssDNA/RNA Clean & Concentrator^TM^ kit, and the DNA primer and template were removed by incubating the purified product with 0.5 U/µL DNase I (RNase-free) in 1× DNase I reaction buffer at 37 °C for 16 h. The DNase I degradation product was purified and concentrated with a Zymo ssDNA/RNA Clean & Concentrator^TM^ kit to produce the 5’-end-Dabcyl and 3’-end-dTPT3^Am^-dual-labelled 2’-F-CU-RNA aptamer. Then the product was mixed with 1 mM 6-FAM-NHS ester in 1× PBS buffer (pH = 8.5) and incubated at 37 °C for 2 h. The produced 5’-end-Dabcyl and 3’-end-FAM-dual-labelled 2’-F-CU-RNA aptamer (aptasensor) was purified and concentrated with a Zymo ssDNA/RNA Clean & Concentrator^TM^ kit and quantified with Nano Drop 2000. All the reaction products were analyzed with a 20% denaturing PAGE gel containing 8 M urea. The gel was visualized using a gel imager before and after staining with Cyber gold. Aptamer Y-toggle-25t labelled with only the 5’-end FAM, FAM-Y-toggle-25t, was also prepared for the control experiments as described above.

To test the performance of this aptasensor for the detection of human α-thrombin, 0.1 μM of this aptasensor was folded in 1× binding buffer by incubating at 95 °C for 10 min, slowly cooling down to room temperature, and then incubating on ice for 10 min. For the thrombin detection experiment, four solutions were prepared: (1) 1× PBS buffer (pH = 7.4) containing 10 nM of the aptasensor without folding, (2) 1× PBS buffer (pH = 7.4) containing 10 nM of the folded aptasensor, (3) 1× PBS buffer (pH = 7.4) containing 10 nM of the folded aptasensor and 100 nM thrombin, and (4) 1× PBS buffer (pH = 7.4) containing 10 nM of the folded aptasensor and 100 nM BSA. For the control experiments, the aptasensor in each reaction solution was replaced with FAM-Y-toggle-25t. 100 μL of each of the solutions was added into a well of a black 96-well plate, and the plate was incubated at 37 °C for 30 min before the fluorescence intensity was read with a plate reader (excitation: 485 nm; emission: 535 nm).

### Statistics and reproducibility

All experiments were performed with at least three independent biological replicates. For experiments where representative gel electrophoresis images, confocal images, and flow cytometry analysis are shown in the manuscript and supplementary information file, similar results were obtained across three independent biological replicates.

### Reporting summary

Further information on research design is available in the Nature Portfolio Reporting Summary linked to this article.

## Supplementary information

Supplementary Information

Reporting Summary

Transparent Peer Review file

## Source data

Source data

## Data Availability

Source data are provided with this paper. The raw data generated in this study are provided in the Supplementary Information and the Source Data file. Source data are provided with this paper.
