# Supplementary material for: Engineered polymerase-mediated efficient synthesis of site-specifically functionalized RNA and 2’-modified RNA oligonucleotides via genetic alphabet expansion
Source: Nat Commun. 2026 Aug 13;17:9705. doi: 10.1038/s41467-026-76347-0 (PMC13562680; doi:10.1038/s41467-026-76347-0)
Supplement: Supplementary file 1 [file 41467_2026_76347_MOESM1_ESM.pdf]

Supplementary Information

Engineered polymerase-mediated efficient synthesis of site-specifically functionalized RNA and 2'-modified RNA oligonucleotides via genetic alphabet expansion

Jing Wu[1†], Junyan Mei[1†], Zhipeng Chen[1†], Xinyu Shao[1], Yaxin Wang[1], Ziqian Xiao[1], Yuhui Du[1] and Tingjian Chen[1*]

[1]MOE International Joint Research Laboratory on Synthetic Biology and Medicines, School of Biology and Biological Engineering, South China University of Technology, Guangzhou 510006, PR China

[†] These authors contributed equally to this work: J. W., J. M. and Z. C.

*** Correspondence:**

Tingjian Chen

chentj@scut.edu.cn

Contents

1. Supplementary Figures……………………………………………...…….……S3

2. Supplementary Tables…………………………………………………………S44

3. Supplementary Note…………………………………………………………...S47

Supplementary Figures

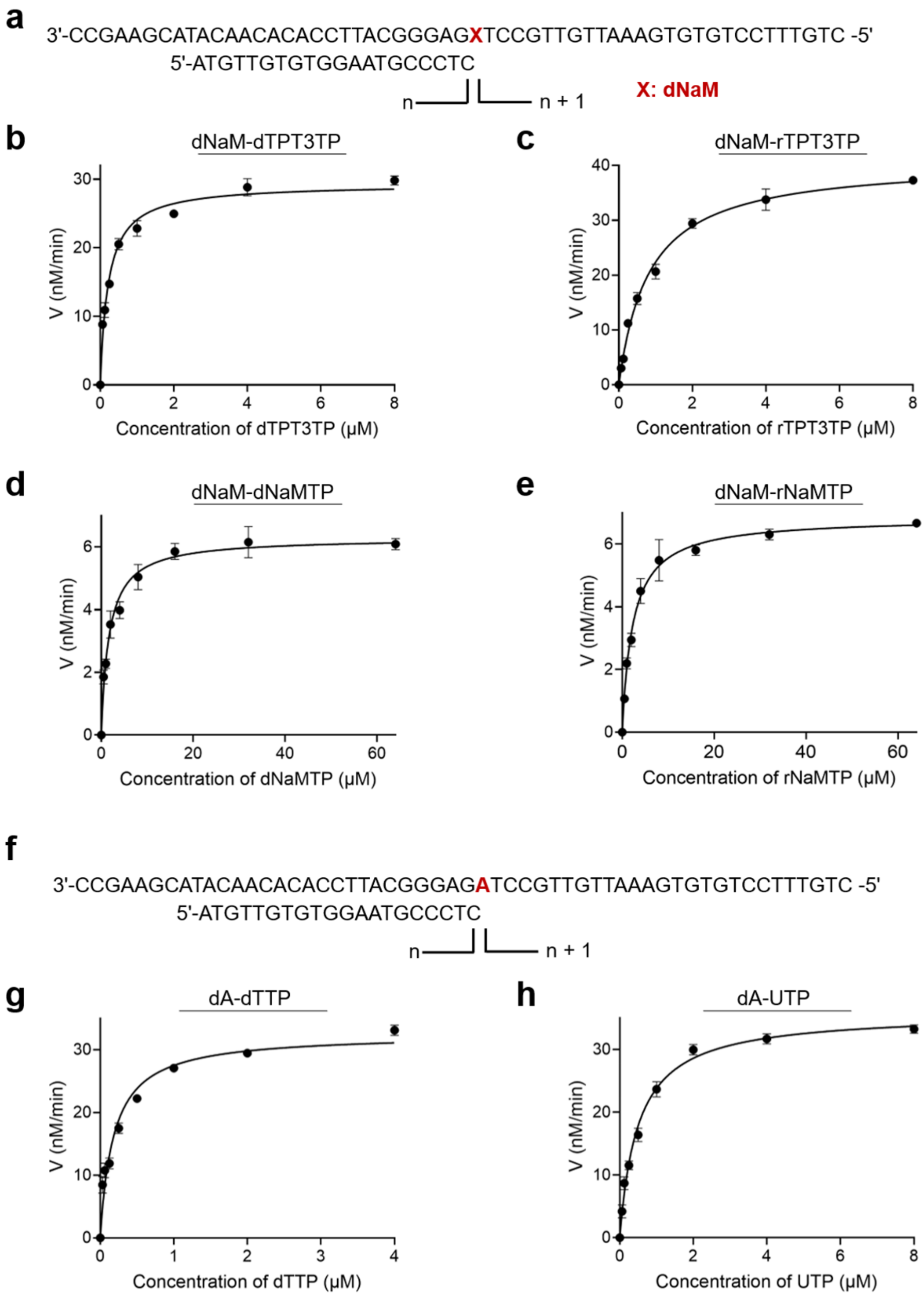

Supplementary Figure 1. Michaelis-Menten plots for SFM4-6-mediated incorporation of natural and unnatural nucleoside triphosphates opposite a dNaM or dA nucleotide in the DNA template. (**a**) Schematic diagram for the incorporation of dTPT3TP, rTPT3TP, dNaMTP, or rNaMTP opposite a dNaM nucleotide in DNA template T55-X. (**b**)

Incorporation of dTPT3TP opposite the dNaM nucleotide in the template. (**c**) Incorporation of rTPT3TP opposite the dNaM nucleotide in the template. (**d**) Incorporation of dNaMTP opposite the dNaM nucleotide in the template. (**e**) Incorporation of rNaMTP opposite the dNaM nucleotide in the template. (**f**) Schematic diagram for the incorporation of dTTP or UTP opposite a dA nucleotide in DNA template T55-A. (**g**) Incorporation of dTTP opposite the dA nucleotide in the template. (**h**) Incorporation of UTP opposite the dA nucleotide in the template. All kinetic measurements were performed with three independent biological replicates ($n = 3$), and error bars indicate standard deviations from the means. Source data are provided in the Source Data file.

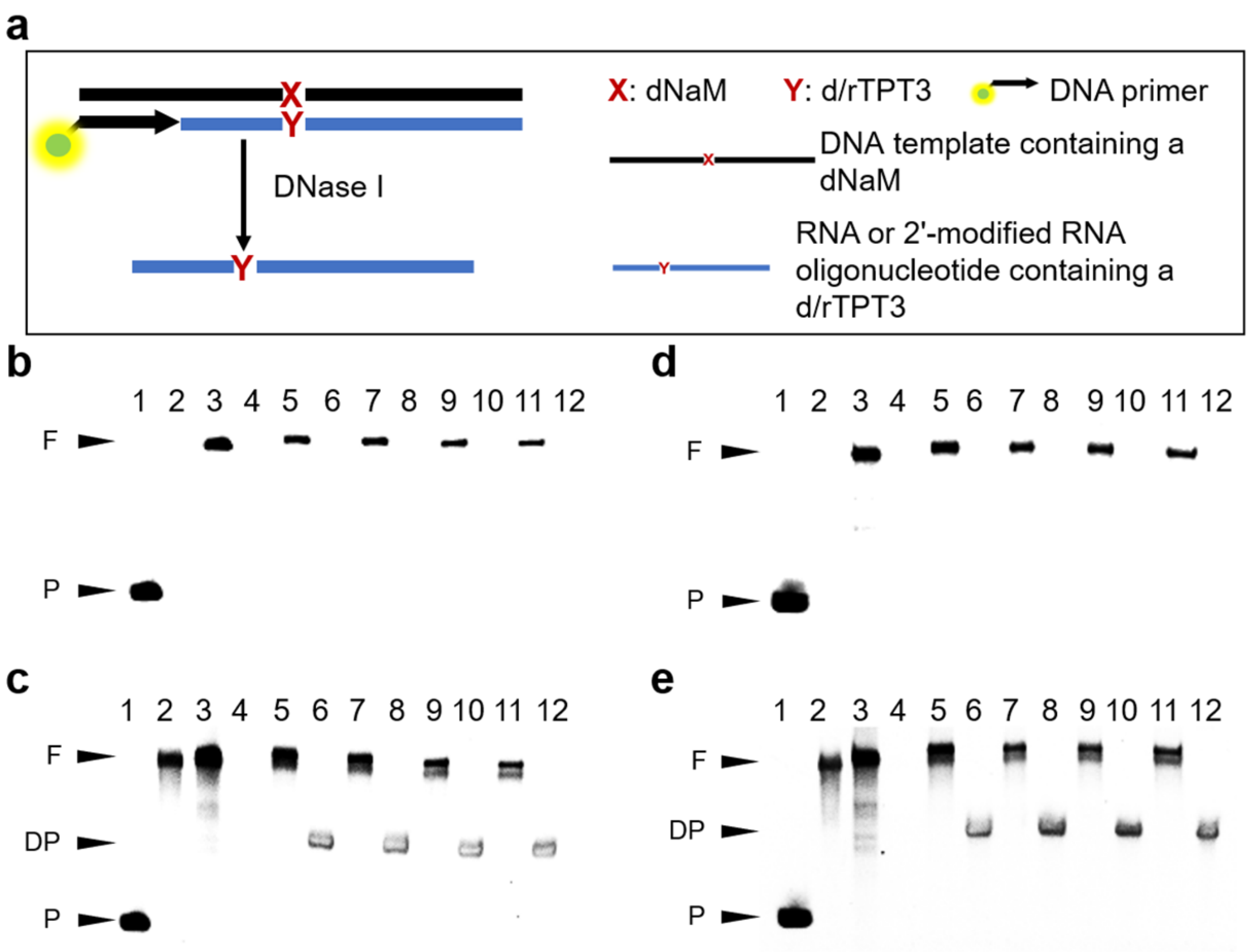

Supplementary Figure 2. Preparation of RNA, 2'-F-CU-RNA, 2'-F/OMe-RNA, and 2'-OMe-RNA oligonucleotides containing a d/rTPT3 nucleotide by DNase I degradation of the primer extension product. (**a**) Schematic diagram for the preparation of RNA, 2'-F-CU-RNA, 2'-F/OMe-RNA, and 2'-OMe-RNA oligonucleotides containing a d/rTPT3 nucleotide by DNase I degradation of the product of primer extension, in which a d/rTPT3 nucleotide was incorporated opposite a dNaM nucleotide in the template during the synthesis of RNA, 2'-F-CU-RNA, 2'-F/OMe-RNA, or 2'-OMe-RNA oligonucleotide. (**b**-**e**) Gel analysis of the primer extension and the DNase I degradation products containing a dTPT3 (**b** and **c**) or rTPT3 (**d** and **e**) nucleotide. The products were analyzed with 20% denaturing PAGE gels containing 8 M urea, and the gels were visualized before (**b** and **d**) and after (**c** and **e**) staining with Cyber Gold. Lane 1: FAM-labelled DNA primer FAM-P-18; lane 2: DNA template T55-X; lanes 3, 5, 7, 9, and 11: primer extension with dNTPs (3), NTPs (5), ATP, 2'-F-UTP, 2'-F-CTP, and GTP (7), 2'-OMe-ATP, 2'-F-UTP, 2'-F-CTP, and 2'-OMe-GTP (9), and 2'-OMe-NTPs (11); lanes 4, 6, 8, 10, and 12: DNase I degradation products of the primer extension products in lanes 3 (4), 5 (6), 7 (8), 9 (10), and 11(12). P: FAM-labelled DNA

primer FAM-P-18 (18 nt); DP: Degradation products; F: full-length extension product. Source data are provided in the Source Data file.

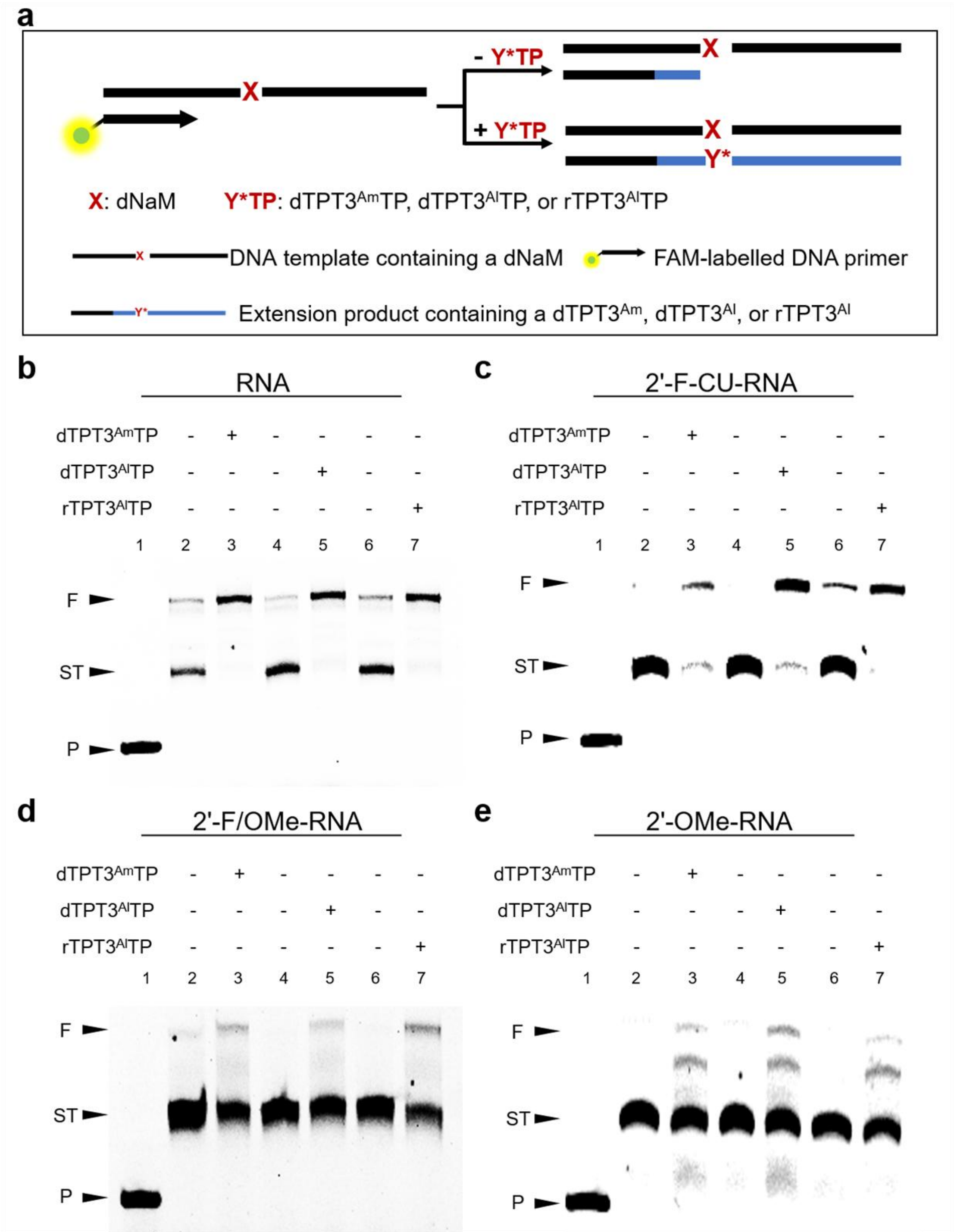

Supplementary Figure 3. Primer extension assay for SFM4-6-mediated incorporation of dTPT3^{Am}TP, dTPT3^{Al}TP, or rTPT3^{Al}TP opposite a dNaM nucleotide at a specific internal position of the DNA template during the synthesis of RNA, 2'-F-CU-RNA, 2'-F/OMe-RNA, or 2'-OMe-RNA. (**a**) Schematic diagram of the primer extension assay for SFM4-6-mediated incorporation of dTPT3^{Am}TP, dTPT3^{Al}TP, or rTPT3^{Al}TP opposite a dNaM nucleotide at a specific internal position of the DNA template during

the synthesis of RNA, 2'-F-CU-RNA, 2'-F/OMe-RNA, or 2'-OMe-RNA. (**b**-**e**) Gel analysis for the products of the primer extension reactions. The products were analyzed with 20% denaturing PAGE gels containing 8 M urea, and the gels were directly visualized without staining. Lane 1: FAM-labelled DNA primer FAM-P-18; lanes 2, 4, and 6: primer extension with NTPs (**b**), ATP, 2'-F-UTP, 2'-F-CTP, and GTP (**c**), 2'-OMe-ATP, 2'-F-UTP, 2'-F-CTP, and 2'-OMe-GTP (**d**), and 2'-OMe-NTPs (**e**) in the absence of dTPT3^{Am}TP, dTPT3^{Al}TP, and rTPT3^{Al}TP; lanes 3, 5, and 7: primer extension with NTPs (**b**), ATP, 2'-F-UTP, 2'-F-CTP, and GTP (**c**), 2'-OMe-ATP, 2'-F-UTP, 2'-F-CTP, and 2'-OMe-GTP (**d**), and 2'-OMe-NTPs (**e**) in the presence of dTPT3^{Am}TP (3), dTPT3^{Al}TP (5), or rTPT3^{Al}TP (7). For primer extension with NTPs or ATP, 2'-F-UTP, 2'-F-CTP, and GTP, 40 nM SFM4-6 was used, and the reaction was incubated at 50 °C for 30 min. For primer extension with 2'-OMe-ATP, 2'-F-UTP, 2'-F-CTP, and 2'-OMe-GTP or 2'-OMe-NTPs, 1 mM $MnCl_2$ and 200 nM SFM4-6 was used, and the reaction was incubated at 50 °C for 4 h. P: primer FAM-P-18 (18 nt); ST: product of primer extension stalled at the position opposite the dNaM nucleotide in the template; F: full-length primer extension product. Source data are provided in the Source Data file.

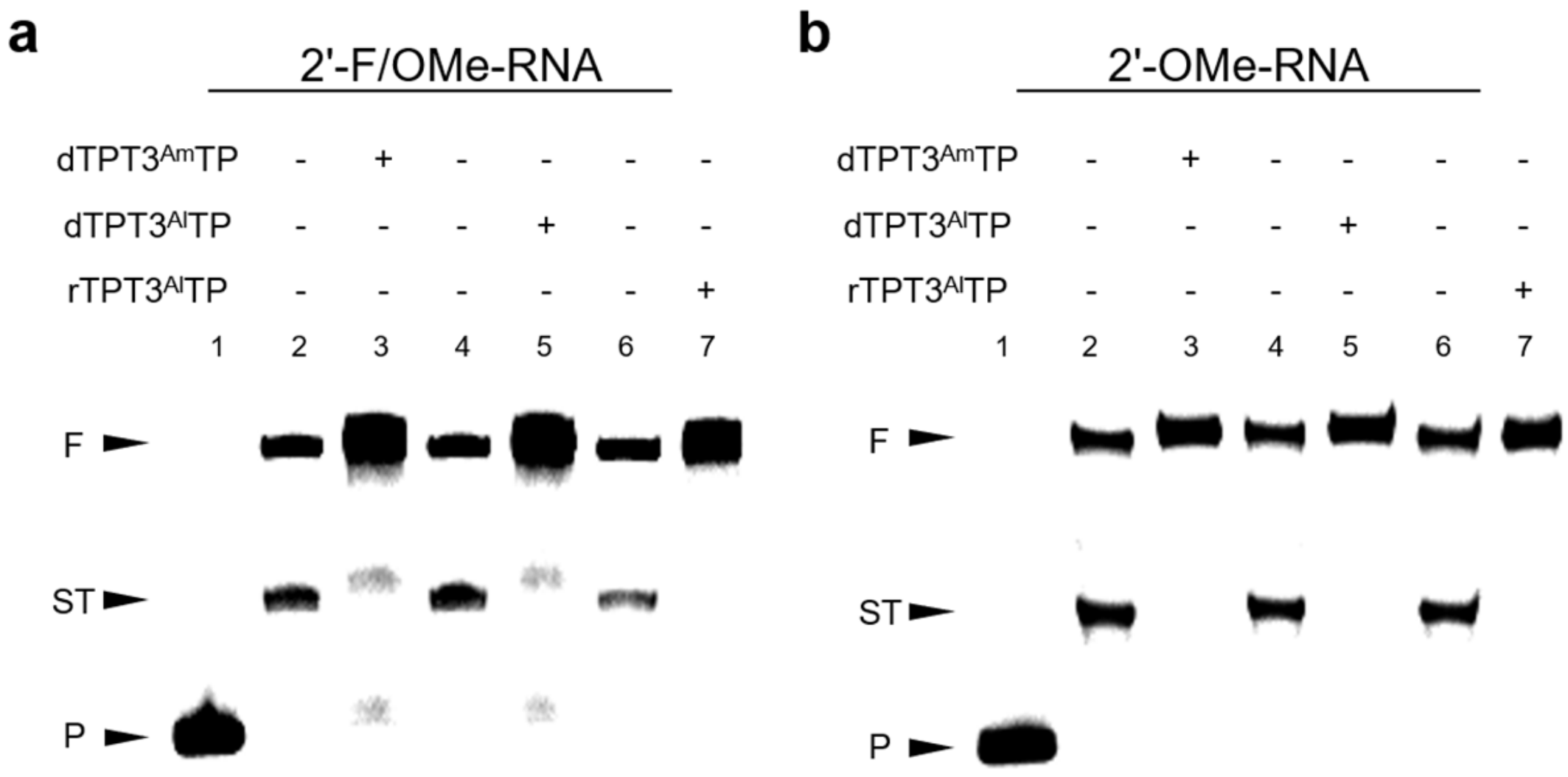

Supplementary Figure 4. Primer extension assay for SFM4-6-mediated incorporation of $dTPT3^{Am}TP$, $dTPT3^{Al}TP$, or $rTPT3^{Al}TP$ opposite a dNaM nucleotide at a specific internal position of the DNA template during the synthesis of 2'-F/OMe-RNA (**a**) or 2'-OMe-RNA (**b**) with an elongated reaction time and a thermocycling program. The products were analyzed with 20% denaturing PAGE gels containing 8 M urea, and the gels were directly visualized without staining. Lane 1: FAM-labelled DNA primer FAM-P-18; lanes 2, 4, and 6: primer extension with 2'-OMe-ATP, 2'-F-UTP, 2'-F-CTP, and 2'-OMe-GTP (**a**) and 2'-OMe-NTPs (**b**) in the absence of $dTPT3^{Am}TP$, $dTPT3^{Al}TP$, and $rTPT3^{Al}TP$; lanes 3, 5, and 7: primer extension with 2'-OMe-ATP, 2'-F-UTP, 2'-F-CTP, and 2'-OMe-GTP (**a**) and 2'-OMe-NTPs (**b**) in the presence of $dTPT3^{Am}TP$ (3), $dTPT3^{Al}TP$ (5), or $rTPT3^{Al}TP$ (7). 1 mM $MnCl_2$ and 200 nM SFM4-6 was used, and the reaction was carried out with the following thermocycling program: 6 cycles of (50 °C, 2 h; 70 °C, 30 min); 50 °C, 2 h. P: primer FAM-P-18 (18 nt); ST: product of primer extension stalled at the position opposite the dNaM nucleotide in the template; F: full-length primer extension product. Source data are provided in the Source Data file.

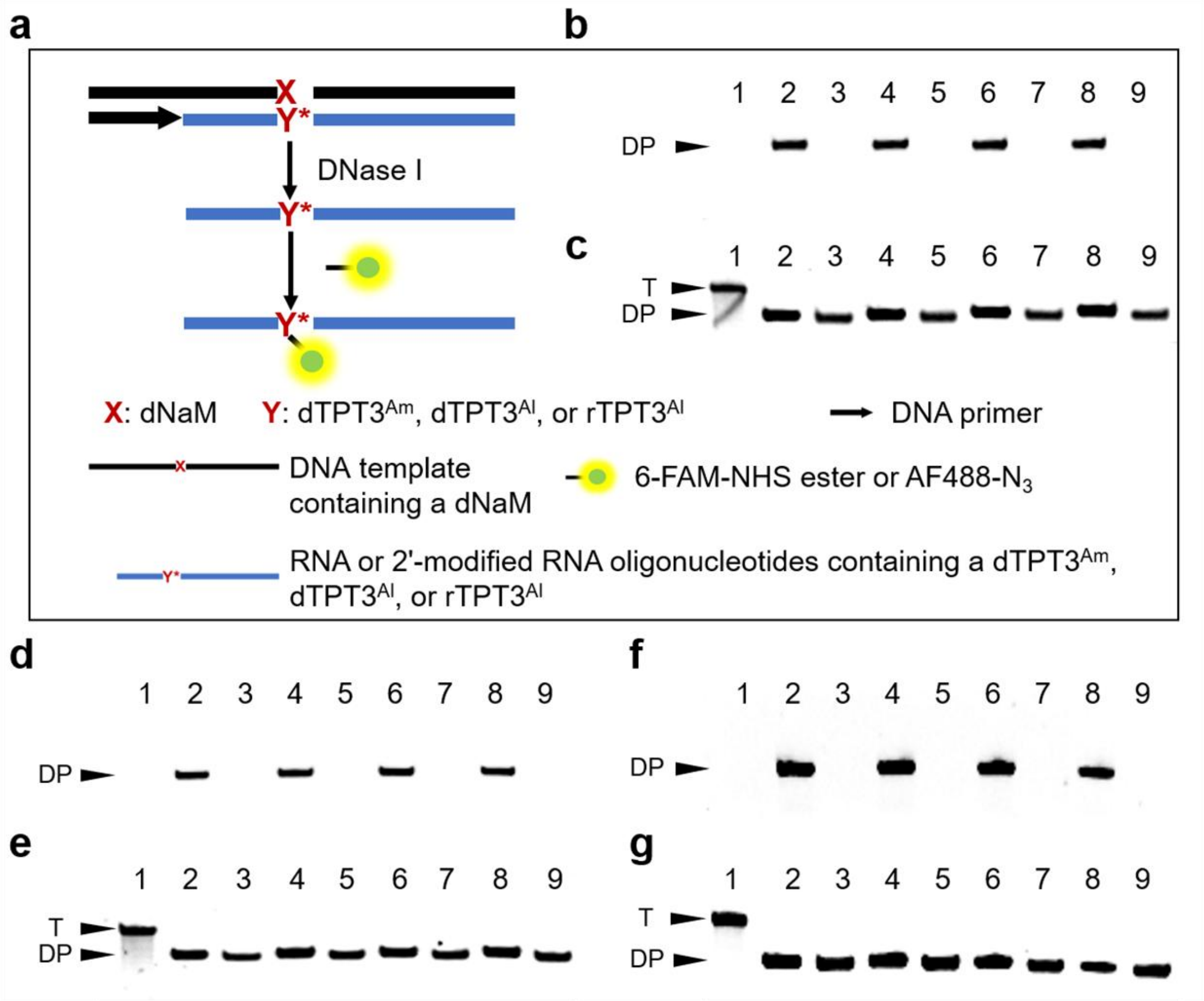

Supplementary Figure 5. Test for the fluorescent labelling of unnatural base (UB)-containing RNA, 2'-F-CU-RNA, 2'-F/OMe-RNA, and 2'-OMe-RNA oligonucleotides prepared by SFM4-6-mediated primer extension and DNase I degradation of the primer extension product. (**a**) Schematic diagram for the experiment. (**b-d**) Gel analysis of the prepared RNA, 2'-F-CU-RNA, 2'-F/OMe-RNA, and 2'-OMe-RNA oligonucleotides containing dTPT3^{Am} (**b** and **c**), dTPT3^{Al} (**d** and **e**), or rTPT3^{Al} (**f** and **g**) and the fluorescent labelling products of these oligonucleotides prepared by coupling with 6-FAM-NHS ester (**b** and **c**) or AF488-N_3 (**d, e, f,** and **g**). The products were analyzed with 20% denaturing PAGE gels containing 8 M urea, and the gels were visualized before (**b, d,** and **f**) and after (**c, e,** and **g**) staining with Cyber Gold. Lane 1: DNA template T55-X; lanes 2, 4, 6, and 8: the prepared RNA (2), 2'-F-CU-RNA (4), 2'-F/OMe-RNA (6), and 2'-OMe-RNA (8) oligonucleotides containing dTPT3^{Am} (**b** and **c**), dTPT3^{Al} (**d** and **e**), or rTPT3^{Al} (**f** and **g**) fluorescently labelled by coupling with 6-FAM-NHS ester (**b** and **c**) or N_3-AF488 (**d, e, f,** and **g**); lanes 3, 5, 7, and 9: the prepared

RNA (3), 2'-F-CU-RNA (5), 2'-F/OMe-RNA (7), and 2'-OMe-RNA (9) oligonucleotides containing dTPT3^{Am} (**b** and **c**), dTPT3^{Al} (**d** and **e**), or rTPT3^{Al} (**f** and **g**) without fluorescent labelling. T: DNA template T55-X (55 nt); DP: the degradation products with or without fluorescent labelling. Source data are provided in the Source Data file.

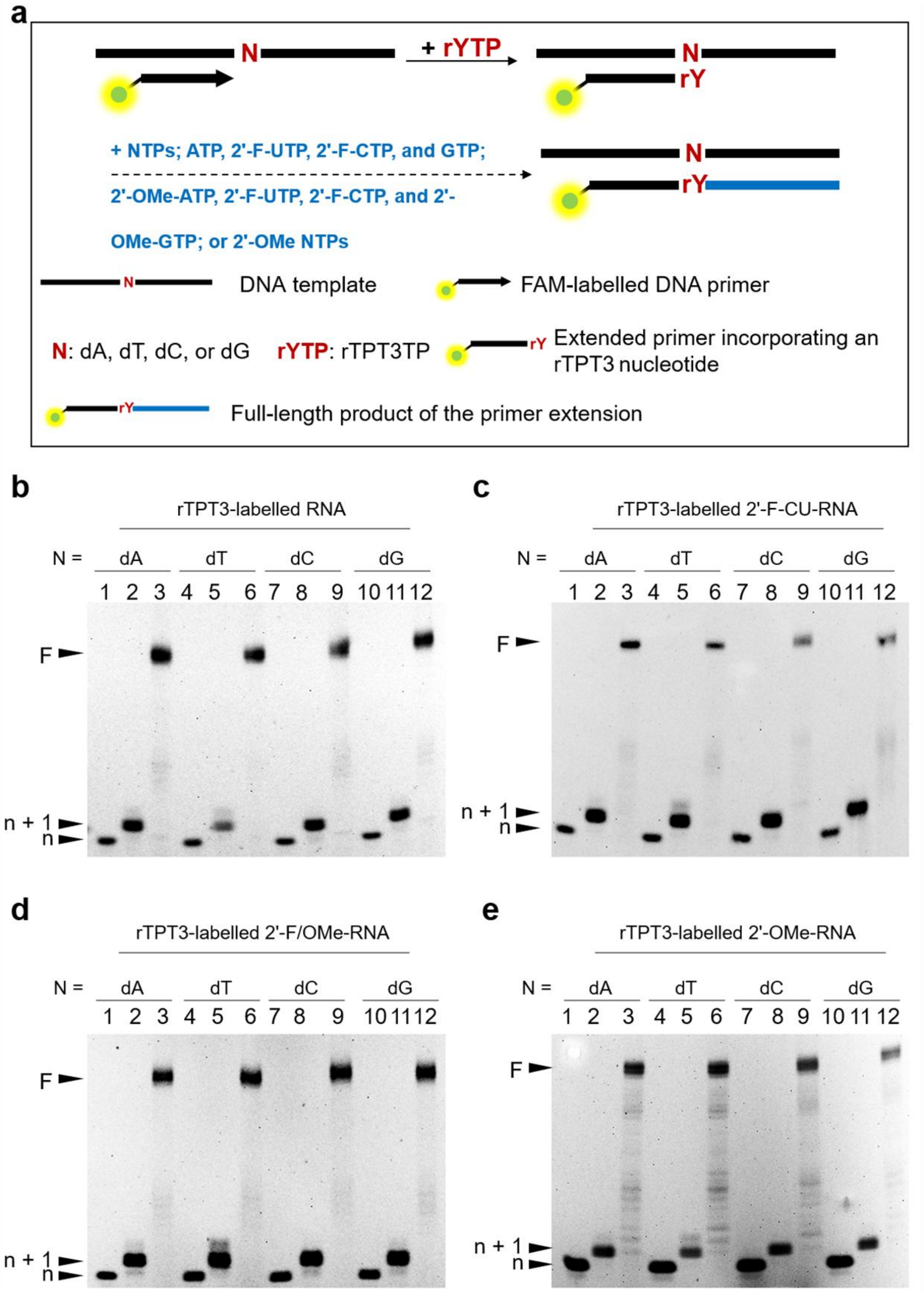

Supplementary Figure 6. SFM4-6-mediated primer extension with controlled pause and restart for the enzymatic synthesis of 5'-end-rTPT3-labelled RNA, 2'-F-CU-RNA, 2'-F/OMe-RNA, and 2'-OMe-RNA oligonucleotides. (**a**) Schematic diagram of SFM4-6-mediated primer extension by the synthesis of RNA, 2'-F-CU-RNA, 2'-F/OMe-RNA,

or 2'-OMe-RNA after the incorporation of an rTPT3 nucleotide opposite a natural nucleotide in the template. (**b**-**e**) Gel analysis for the products of SFM4-6-mediated primer extension by the synthesis of RNA, 2'-F-CU-RNA, 2'-F/OMe-RNA, or 2'-OMe-RNA after the incorporation of an rTPT3 nucleotide opposite a natural nucleotide in the template. (**b**-**e**) The products for primer extension with NTPs (**b**), ATP, 2'-F-UTP, 2'-F-CTP, and GTP (**c**), 2'-OMe-ATP, 2'-F-UTP, 2'-F-CTP, and 2'-OMe-GTP (**d**), or 2'-OMe-NTPs (**e**) after the incorporation of an rTPT3 nucleotide opposite a natural nucleotide. The products were analyzed with 20% denaturing PAGE gels containing 8 M urea, and the gels were directly visualized without staining. Lanes 1, 4, 7, and 10: FAM-labelled DNA primer FAM-P-20; lanes 2, 5, 8, and 11: FAM-P-20 extended by the incorporation of an rTPT3 nucleotide opposite a dA, dT, dC, or dG nucleotide in DNA template T55-A (2), T55-T (5), T55-C (8), or T55-G (11), respectively; lanes 3, 6, 9, and 12: FAM-P-20 extended with NTPs (**b**), ATP, 2'-F-UTP, 2'-F-CTP, and GTP (**c**), 2'-OMe-ATP, 2'-F-UTP, 2'-F-CTP, and 2'-OMe-GTP (**d**), or 2'-OMe-NTPs (**e**) after the incorporation of an rTPT3 nucleotide opposite a dA, dT, dC, or dG nucleotide in DNA template T55-A (3), T55-T (6), T55-C (9), or T55-G (12), respectively. n: primer FAM-P-20 (20 nt); n + 1: FAM-P-20 extended by the incorporation of an rTPT3 nucleotide (21 nt); F: full-length product of the primer extension. Source data are provided in the Source Data file.

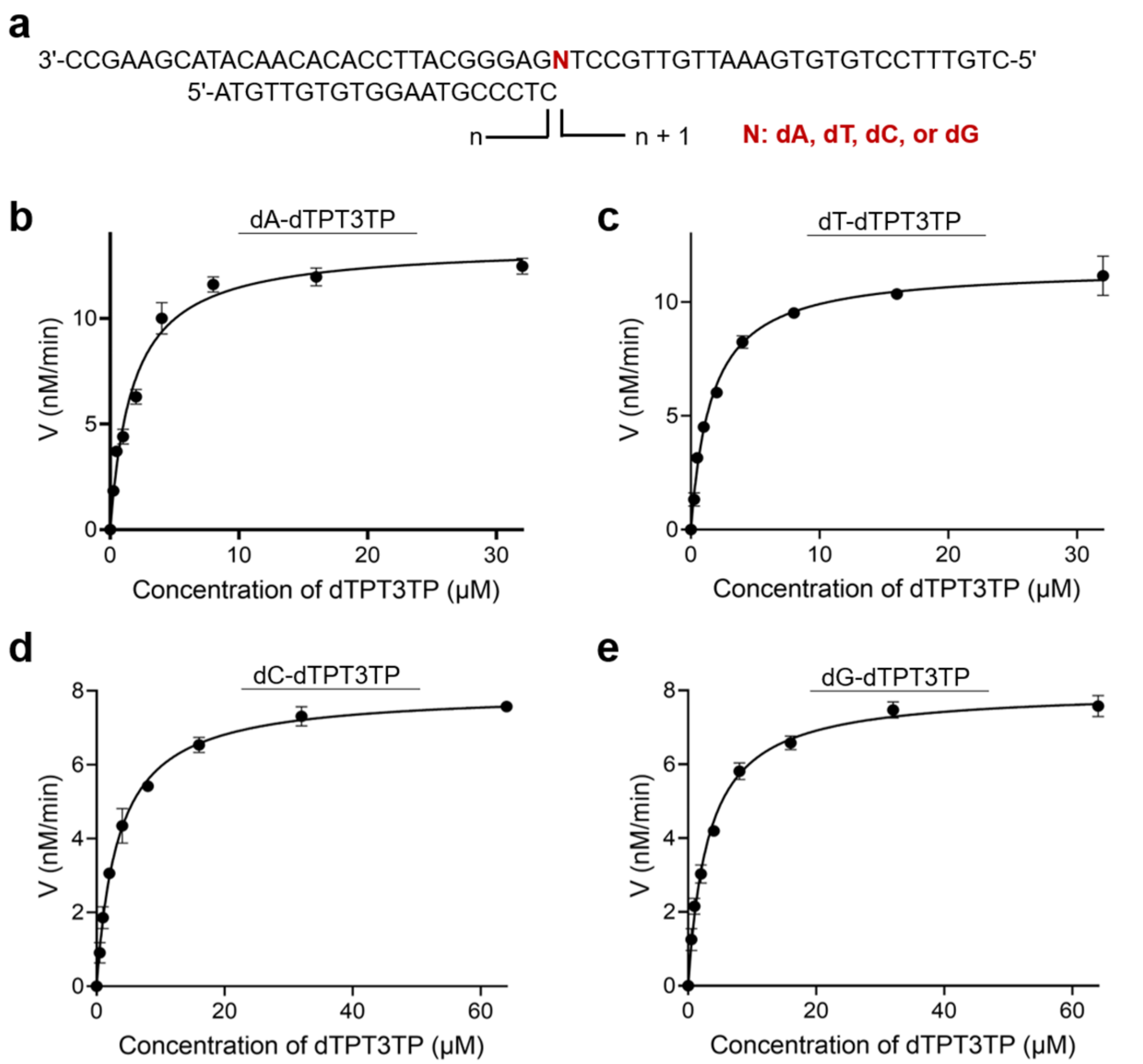

Supplementary Figure 7. Michaelis-Menten plots for SFM4-6-mediated incorporation of dTPT3TP opposite different natural nucleotides in the DNA template. (**a**) Schematic diagram for the incorporation of dTPT3TP opposite a dA, dT, dC, or dG nucleotide in DNA template T55-A/T/C/G. (**b**) Incorporation of dTPT3TP opposite the dA nucleotide in DNA template T55-A. (**c**) Incorporation of dTPT3TP opposite the dT nucleotide in DNA template T55-T. (**d**) Incorporation of dTPT3TP opposite the dC nucleotide in DNA template T55-C. (**e**) Incorporation of dTPT3TP opposite the dG nucleotide in DNA template T55-G. All kinetic measurements were performed with three independent biological replicates (n = 3), and error bars indicate standard deviations from the means. Source data are provided in the Source Data file.

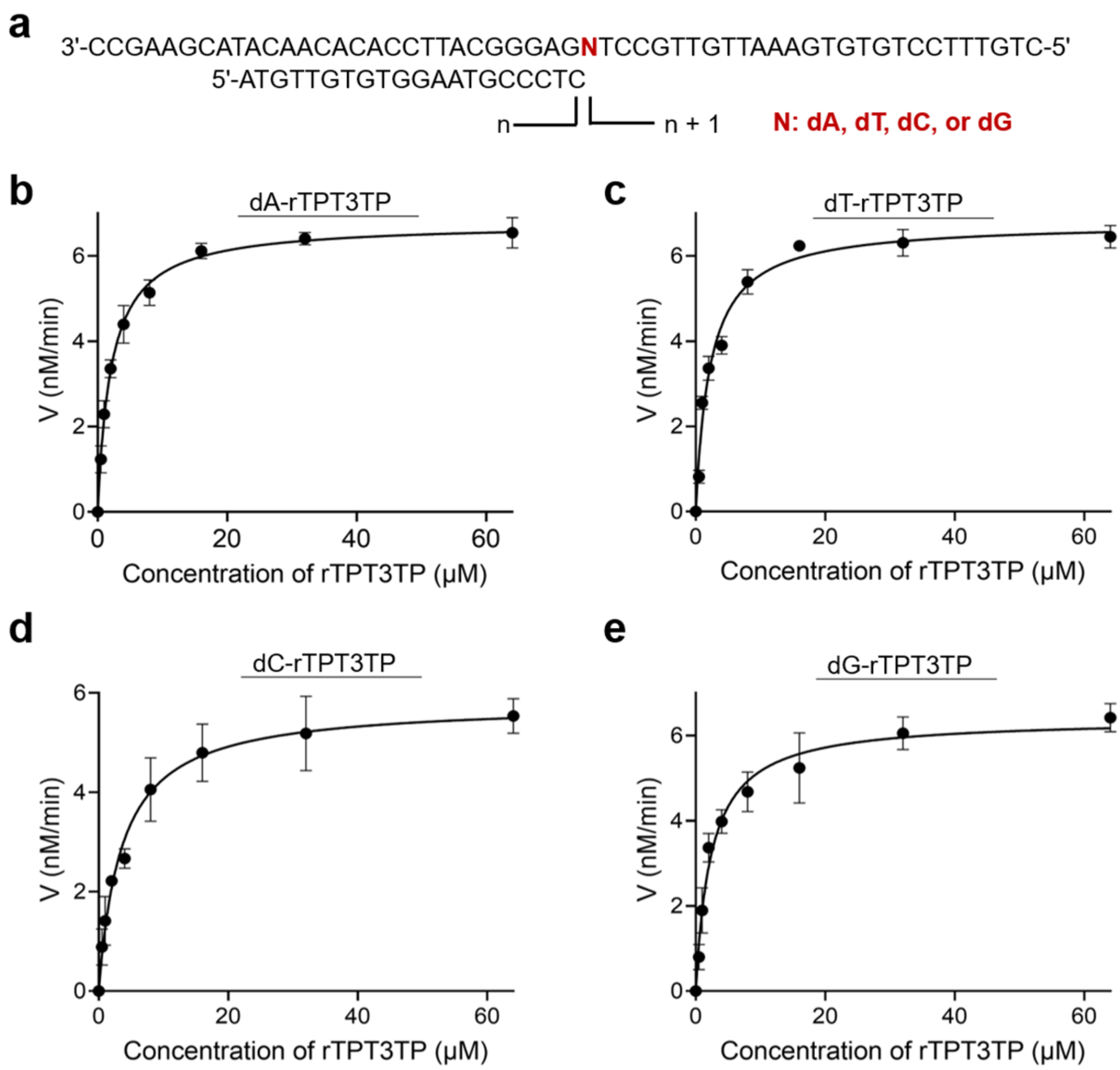

Supplementary Figure 8. Michaelis-Menten plots for SFM4-6-mediated incorporation of rTPT3TP opposite different natural nucleotides in the DNA template. (**a**) Schematic diagram for the incorporation of rTPT3TP opposite a dA, dT, dC, or dG nucleotide in DNA template T55-A/T/C/G. (**b**) Incorporation of rTPT3TP opposite the dA nucleotide in DNA template T55-A. (**c**) Incorporation of rTPT3TP opposite the dT nucleotide in DNA template T55-T. (**d**) Incorporation of rTPT3TP opposite the dC nucleotide in DNA template T55-C. (**e**) Incorporation of rTPT3TP opposite the dG nucleotide in DNA template T55-G. All kinetic measurements were performed with three independent biological replicates (*n* = 3), and error bars indicate standard deviations from the means. Source data are provided in the Source Data file.

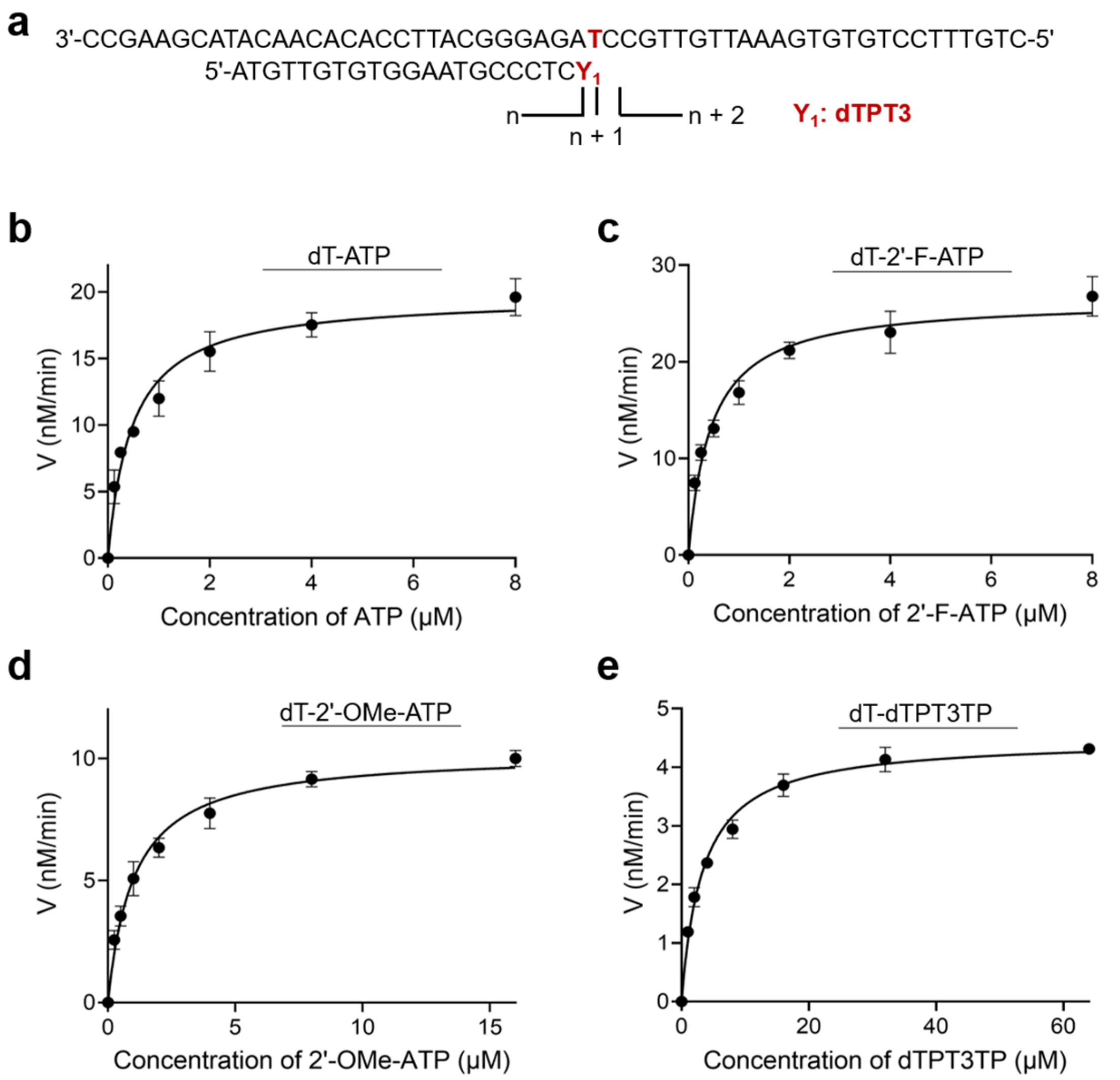

Supplementary Figure 9. Michaelis-Menten plots for SFM4-6-mediated incorporation of a natural or unnatural nucleoside triphosphate opposite a dT nucleotide at the n + 2 position of the DNA template after the incorporation of unnatural nucleoside triphosphate dTPT3TP opposite a dA nucleotide at the n + 1 position. (**a**) Schematic diagram for the incorporation of ATP, 2'-F-ATP, 2'-OMe-ATP, or dTPT3TP opposite a dT nucleotide at the n + 2 position of DNA template T55-A after the incorporation of a dTPT3 nucleotide at the n + 1 position. (**b**) Incorporation of ATP. (**c**) Incorporation of 2'-F-ATP. (**d**) Incorporation of 2'-OMe-ATP. (**e**) Incorporation of dTPT3TP. All kinetic measurements were performed with three independent biological replicates (*n* = 3), and error bars indicate standard deviations from the means. Source data are provided in the Source Data file.

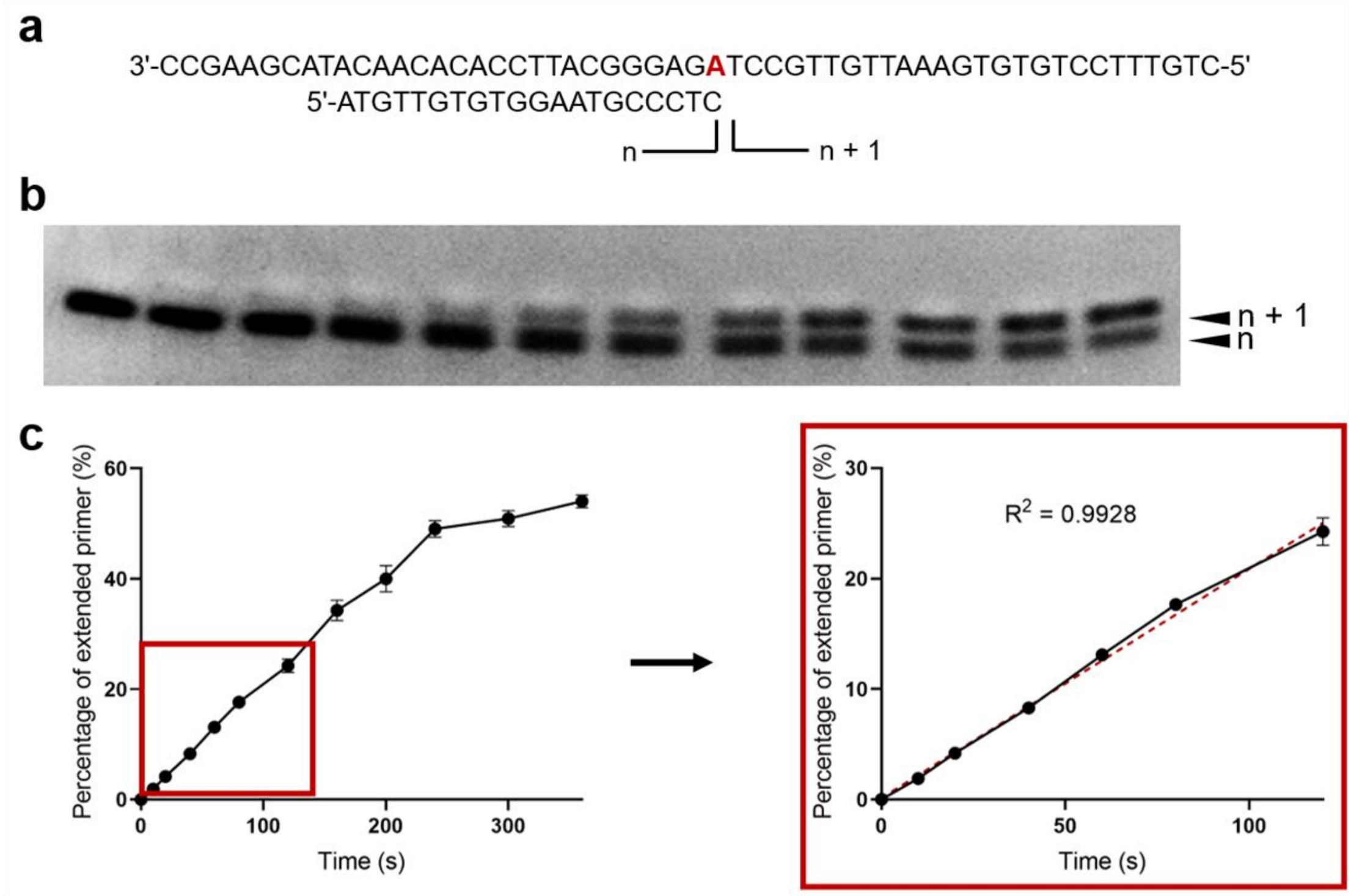

Supplementary Figure 10. Time course of SFM4-6-mediated primer extension by the incorporation of a dTPT3 nucleotide opposite a dA nucleotide in the template. (**a**) Schematic diagram for the primer extension by the incorporation of a dTPT3 nucleotide opposite a dA nucleotide in DNA template T55-A. (**b**) Gel analysis for the products of SFM4-6-mediated primer extension by the incorporation of a dTPT3 nucleotide opposite the dA nucleotide in DNA template T55-A after different reaction times (0, 10 s, 20 s, 40 s, 60 s, 80 s, 120 s, 160 s, 200 s, 240 s, 300 s, and 360 s). The concentration of dTPT3TP in the reaction solutions is 64 μM. The products were analyzed with 20% denaturing PAGE gel containing 8 M urea, and the gel was directly visualized without staining. n: primer FAM-P-20 (20 nt); n + 1: FAM-P-20 extended by the incorporation of a dTPT3 nucleotide (21 nt). (**c**) Correlation between the extension percentage of the primer and the reaction time (in the left panel: 0 - 360 s; in the right panel: 0 - 120 s). The extension percentage of the primer at each time point was calculated from the band intensities (which were obtained using Quantity One software) in the gel pictures, exemplified by (**b**). The experiment was performed with three independent biological replicates (*n* = 3), and error bars indicate standard deviations from the means. In the right panel, linear fitting of the data was carried out, and the fitted line and R^2 value were shown. Source data are provided in the Source Data file.

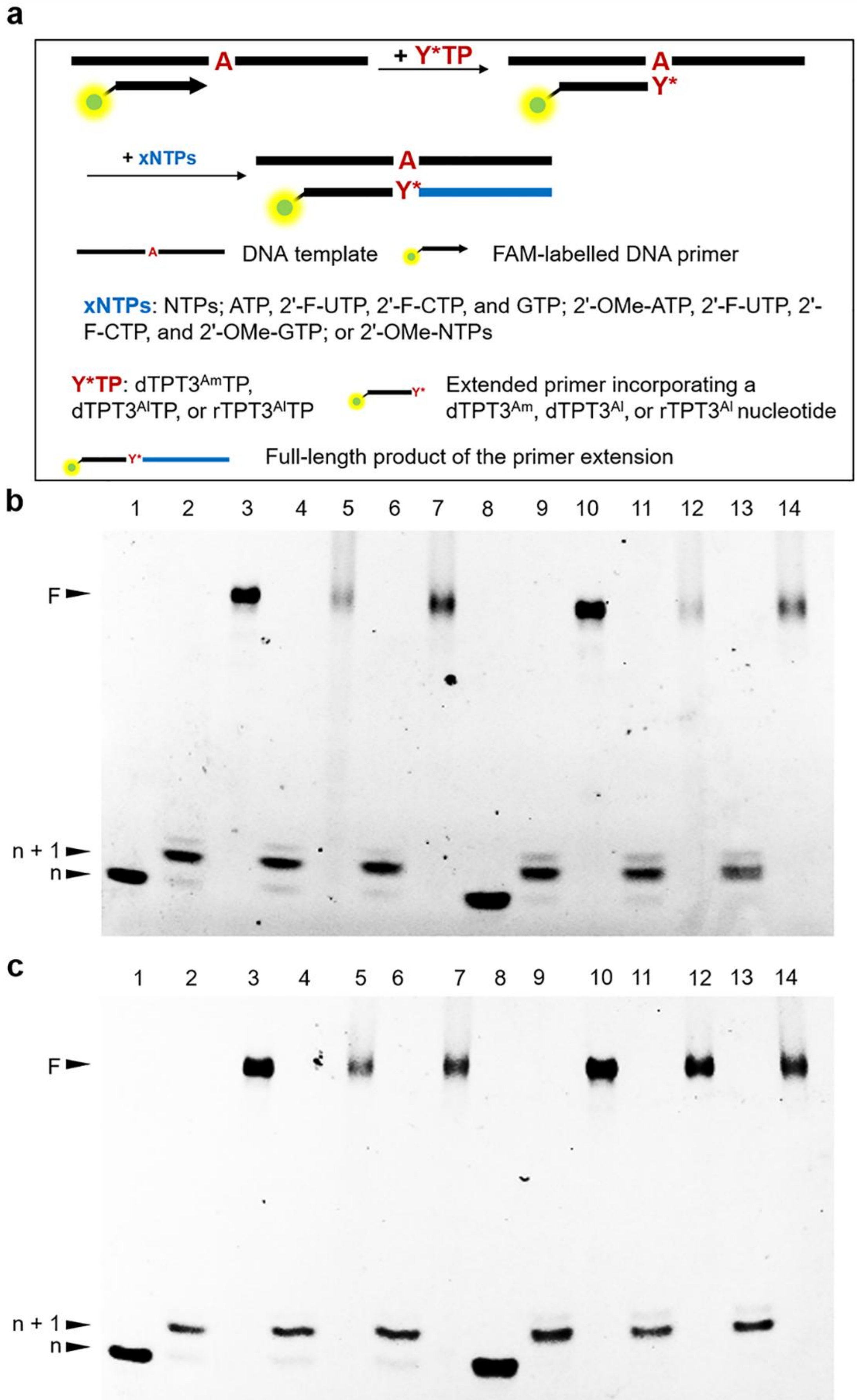
a
A
+ Y*TP
A
Y*
+ xNTPs
A
Y*
DNA template
FAM-labelled DNA primer
xNTPs: NTPs; ATP, 2'-F-UTP, 2'-F-CTP, and GTP; 2'-OMe-ATP, 2'-F-UTP, 2'-F-CTP, and 2'-OMe-GTP; or 2'-OMe-NTPs
Y*TP: dTPT3^Am TP, dTPT3^Al TP, or rTPT3^Al TP
Extended primer incorporating a dTPT3^Am, dTPT3^Al, or rTPT3^Al nucleotide
Full-length product of the primer extension
b
1 2 3 4 5 6 7 8 9 10 11 12 13 14
F
n + 1
n
c
1 2 3 4 5 6 7 8 9 10 11 12 13 14
F
n + 1
n

Supplementary Figure 11. SFM4-6-mediated primer extension with controlled pause and restart for the enzymatic synthesis of 5'-end-dTPT3^{Am}, dTPT3^{Al}, or rTPT3^{Al}-labelled RNA, 2'-F-CU-RNA, 2'-F/OMe-RNA, and 2'-OMe-RNA oligonucleotides. (**a**) Schematic diagram of SFM4-6-mediated primer extension by the synthesis of RNA, 2'-F-CU-RNA, 2'-F/OMe-RNA, or 2'-OMe-RNA after the incorporation of a dTPT3^{Am}, dTPT3^{Al}, or rTPT3^{Al} nucleotide opposite a dA nucleotide in the DNA template. (**b-c**) Gel analysis for the products of SFM4-6-mediated primer extension by the synthesis of RNA or 2'-F-CU-RNA (**b**), or 2'-F/OMe-RNA or 2'-OMe-RNA (**c**) after the incorporation of a dTPT3^{Am}, dTPT3^{Al}, or rTPT3^{Al} nucleotide opposite a dA nucleotide in the DNA template. The products were analyzed with 20% denaturing PAGE gels containing 8 M urea, and the gels were directly visualized without staining. Lanes 1 and 8: FAM-labelled DNA primer FAM-P-20; lanes 2 and 9: FAM-P-20 extended by the incorporation of a dTPT3^{Am} nucleotide; lanes 4 and 11: FAM-P-20 extended by the incorporation of a dTPT3^{Al} nucleotide; lanes 6 and 13: FAM-P-20 extended by the incorporation of an rTPT3^{Al} nucleotide; lanes 3, 5, and 7 in (**b**): primer extension with NTPs after the incorporation of a dTPT3^{Am} (3), dTPT3^{Al} (5), or rTPT3^{Al} (7) nucleotide; lanes 10, 12, and 14 in (**b**): primer extension with ATP, 2'-F-UTP, 2'-F-CTP, and GTP after the incorporation of a dTPT3^{Am} (10), dTPT3^{Al} (12), or rTPT3^{Al} (14) nucleotide; lanes 3, 5, and 7 in (**c**): primer extension with 2'-OMe-ATP, 2'-F-UTP, 2'-F-CTP, and 2'-OMe-GTP after the incorporation of a dTPT3^{Am} (3), dTPT3^{Al} (5), or rTPT3^{Al} (7) nucleotide; lanes 10, 12, and 14 in (**c**): primer extension with 2'-OMe-NTPs after the incorporation of a dTPT3^{Am} (10), dTPT3^{Al} (12), or rTPT3^{Al} (14) nucleotide. n: primer FAM-P-20 (20 nt); n + 1: FAM-P-20 extended by the incorporation of a dTPT3^{Am}, dTPT3^{Al}, or rTPT3^{Al} nucleotide (21 nt); F: full-length product of the primer extension. Source data are provided in the Source Data file.

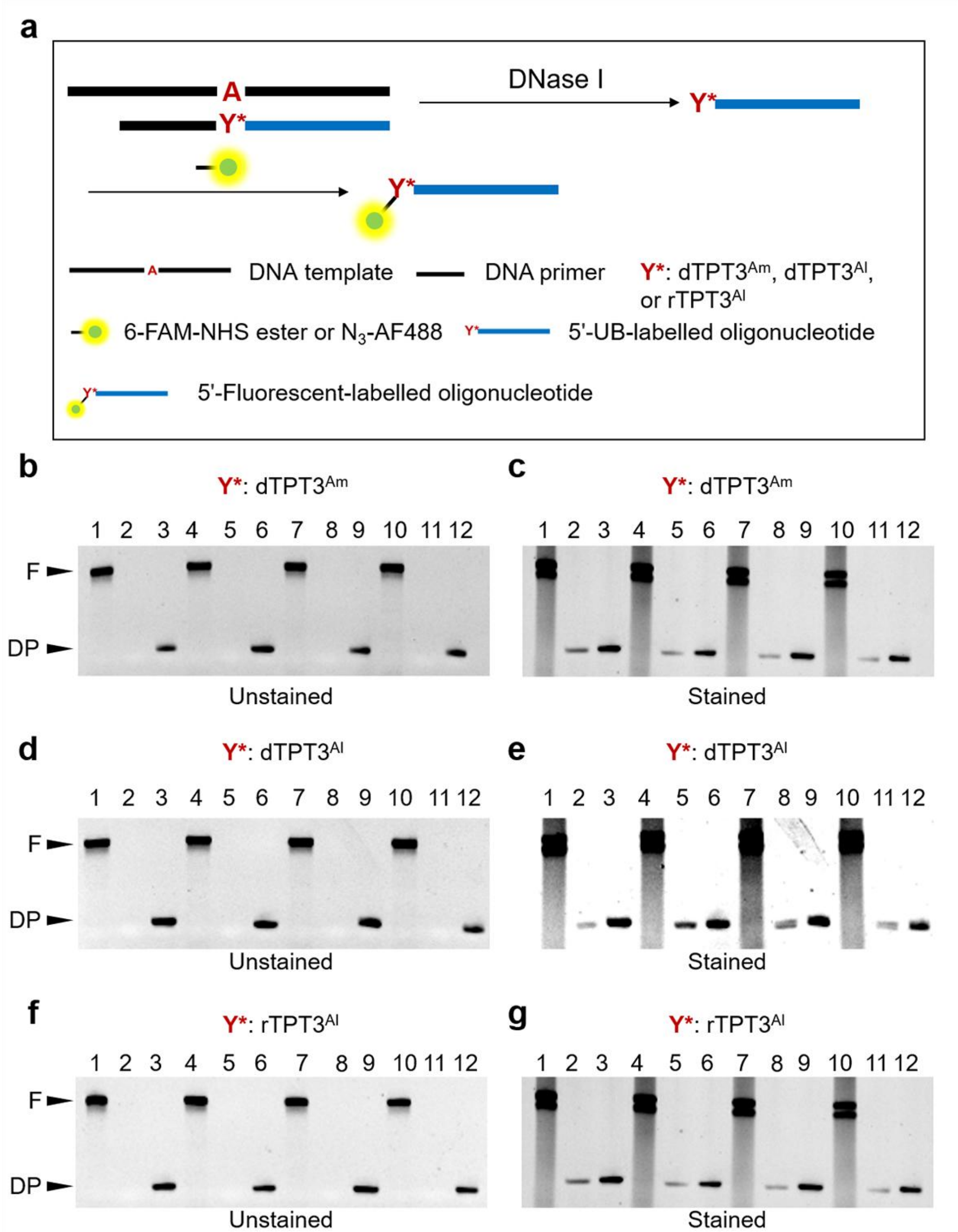

Supplementary Figure 12. Fluorescent labelling of 5'-end-dTPT3^{Am}, dTPT3^{Al}, or rTPT3^{Al}-labelled RNA, 2'-F-CU-RNA, 2'-F/OMe-RNA, and 2'-OMe-RNA oligonucleotides prepared via controlled pause and restart of primer extension with functional analogues of d/rTPT3TP mediated by SFM4-6. (**a**) Schematic diagram for the oligonucleotide preparation and fluorescent labelling experiment. (**b-g**) Gel analysis for the 5'-end-dTPT3^{Am} (**b** and **c**), dTPT3^{Al} (**d** and **e**), or rTPT3^{Al} (**f** and **g**)-labelled

RNA, 2'-F-CU-RNA, 2'-F/OMe-RNA, and 2'-OMe-RNA oligonucleotides prepared via controlled pause and restart of primer extension with functional analogues of d/rTPT3TP mediated by SFM4-6 and the products of coupling these oligonucleotides with 6-FAM-NHS ester (**b** and **c**) or AF488-N_3 (**d, e, f,** and **g**), respectively. The products were analyzed with 20% denaturing PAGE gels containing 8 M urea, and the gels were visualized before (**b, d,** and **f**) and after (**c, e,** and **g**) staining with Cyber Gold. Lanes 1, 4, 7, and 10: primer extension product for the preparation of 5'-dTPT3^{Am} (**b** and **c**), dTPT3^{Al} (**d** and **e**), or rTPT3^{Al} (**f** and **g**)-labelled RNA (1), 2'-F-CU-RNA (4), 2'-F/OMe-RNA (7), or 2'-OMe-RNA (10) oligonucleotide; lanes 2, 5, 8, and 11: 5'-end-dTPT3^{Am} (**b** and **c**), dTPT3^{Al} (**d** and **e**), or rTPT3^{Al} (**f** and **g**)-labelled RNA (2), 2'-F-CU-RNA (5), 2'-F/OMe-RNA (8), or 2'-OMe-RNA (11) oligonucleotide prepared by DNase I degradation of the primer extension product; lanes 3, 6, 9, and 12: the prepared 5'-dTPT3^{Am} (**b** and **c**), dTPT3^{Al} (**d** and **e**), or rTPT3^{Al} (**f** and **g**)-labelled RNA (3), 2'-F-CU-RNA (6), 2'-F/OMe-RNA (9), or 2'-OMe-RNA (12) oligonucleotide coupled with 6-FAM-NHS ester (**b** and **c**) or N_3-AF488 (**d, e, f,** and **g**), respectively. F: full-length product of the primer extension. DP: 5'-end-dTPT3^{Am}, dTPT3^{Al}, or rTPT3^{Al}-labelled RNA, 2'-F-CU-RNA, 2'-F/OMe-RNA, or 2'-OMe-RNA oligonucleotide prepared by DNase I degradation of the primer extension product or the product of coupling the prepared oligonucleotide with 6-FAM-NHS ester or AF488-N_3. Source data are provided in the Source Data file.

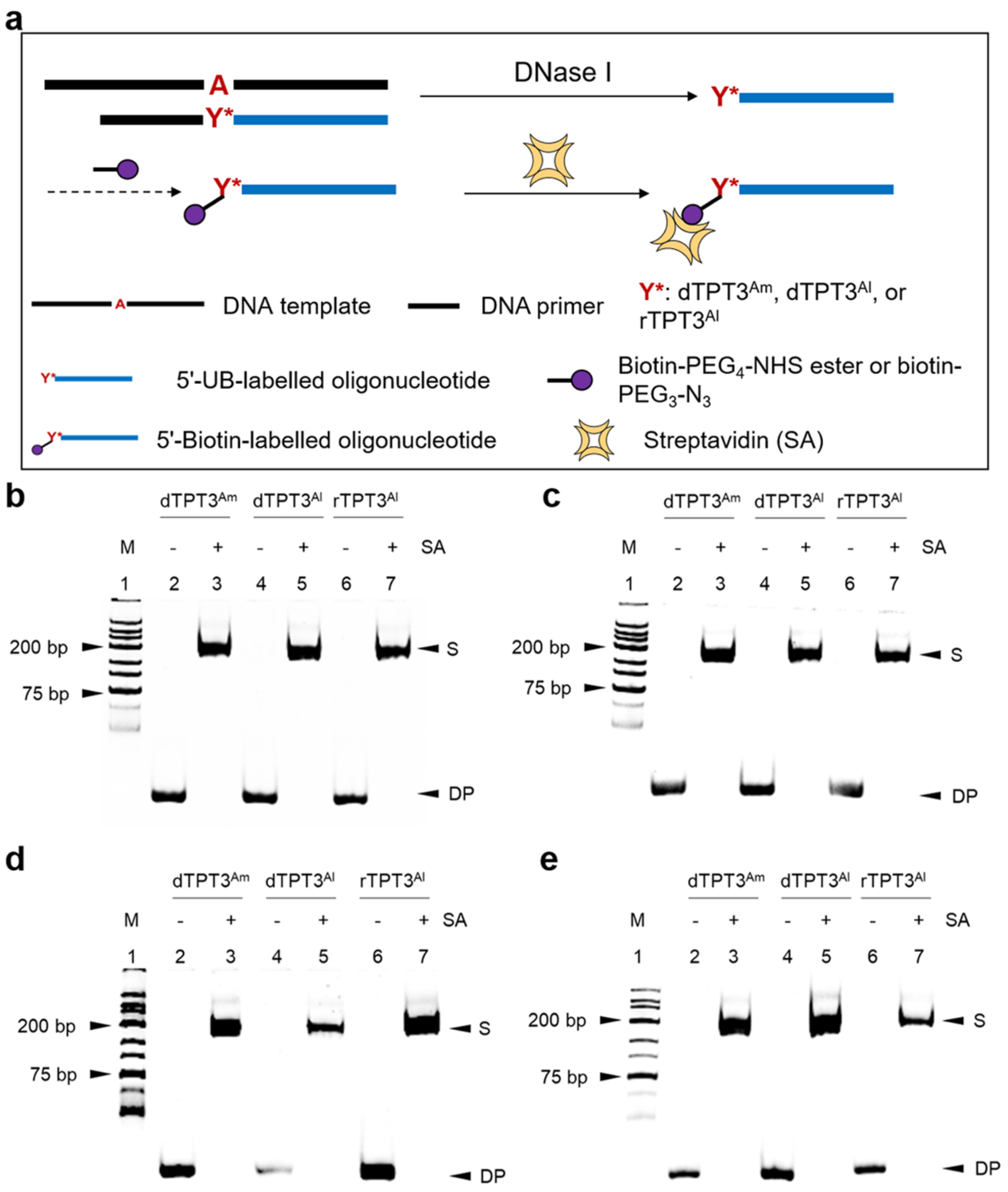

Supplementary Figure 13. Biotin labelling of 5'-end-dTPT3^{Am}, dTPT3^{Al}, or rTPT3^{Al}-labelled RNA, 2'-F-CU-RNA, 2'-F/OMe-RNA, and 2'-OMe-RNA oligonucleotides prepared via controlled pause and restart of primer extension with functional analogues of d/rTPT3TP mediated by SFM4-6 and biotin gel-shift assay of the labelling products. (**a**) Schematic diagram for the preparation and biotin labelling of the oligonucleotides and biotin gel-shift assay of the labelling products. (**b-e**) Biotin gel-shift assay of the 5'-end-dTPT3^{Am}, dTPT3^{Al}, or rTPT3^{Al}-labelled RNA (**b**), 2'-F-CU-RNA (**c**), 2'-F/OMe-RNA (**d**), and 2'-OMe-RNA (**e**) oligonucleotides prepared via controlled pause and

restart of primer extension with functional analogues of d/rTPT3TP mediated by SFM4-6 and subsequently coupled with biotin-PEG_4-NHS ester or biotin-PEG_3-N_3, respectively. The products were analyzed with 9% PAGE gels, and the gels were visualized after staining with Cyber Gold. Lane 1: low molecular weight DNA ladder; lanes 2, 4, and 6: the prepared 5'-end-$dTPT3^{Am}$ (2), $dTPT3^{Al}$ (4), or $rTPT3^{Al}$ (6)-labelled RNA (**b**), 2'-F-CU-RNA (**c**), 2'-F/OMe-RNA (**d**), or 2'-OMe-RNA (**e**) oligonucleotide coupled with biotin-PEG_4-NHS ester (2) or biotin-PEG_3-N_3 (4 and 6), respectively; lanes 3, 5, and 7: the prepared 5'-end-$dTPT3^{Am}$ (3), $dTPT3^{Al}$ (5), or $rTPT3^{Al}$ (7)-labelled RNA (**b**), 2'-F-CU-RNA (**c**), 2'-F/OMe-RNA (**d**), or 2'-OMe-RNA (**e**) oligonucleotide coupled with biotin-PEG_4-NHS ester (3) or biotin-PEG_3-N_3 (5 and 7) and incubated with streptavidin (SA). DP: the oligonucleotide (prepared by DNase I degradation of the primer extension product) coupled with biotin-PEG_4-NHS ester or biotin-PEG_3-N_3; S: the shifted band of the oligonucleotide coupled with biotin-PEG_4-NHS ester or biotin-PEG_3-N_3 upon binding with SA. Source data are provided in the Source Data file.

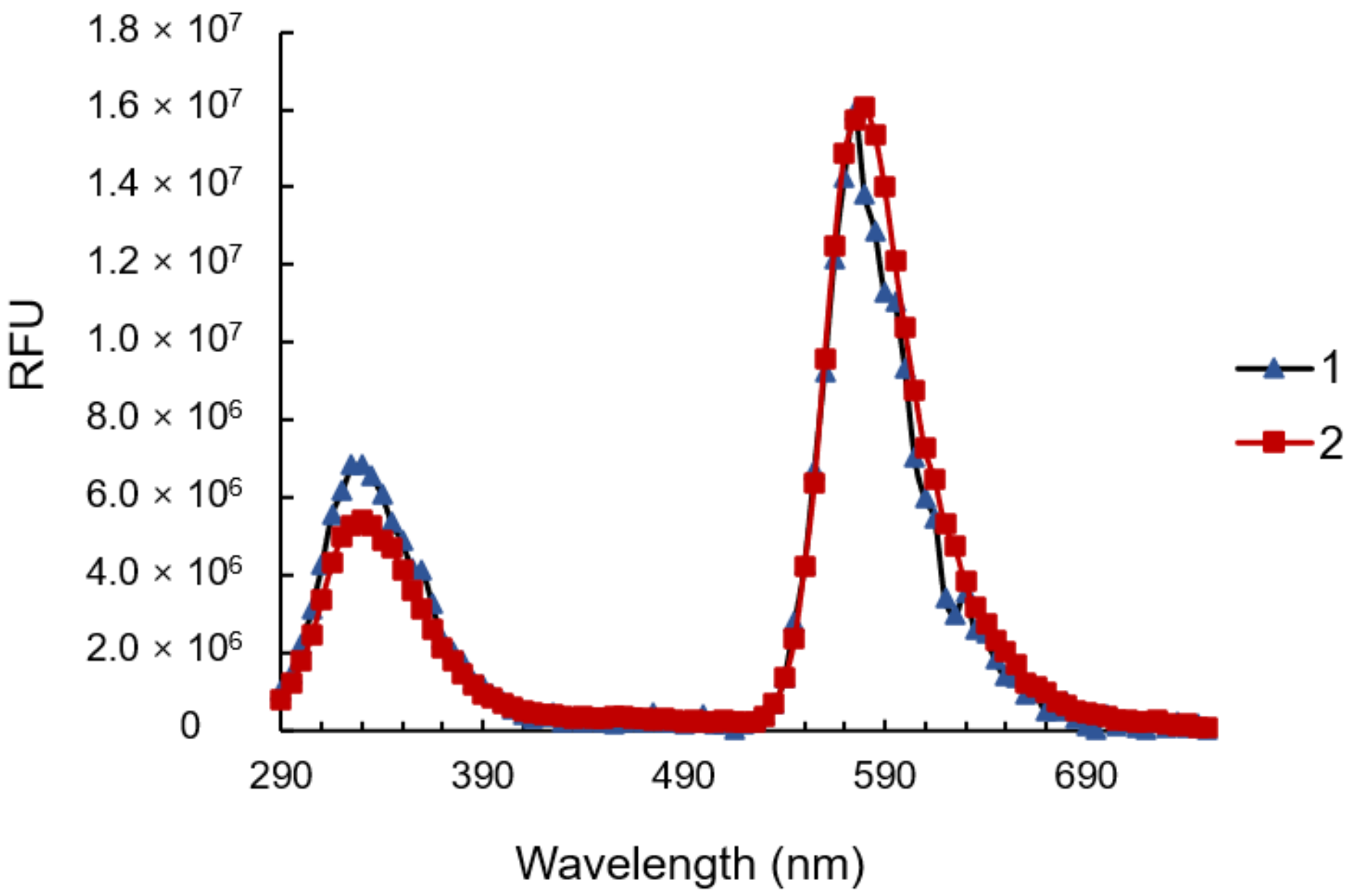

Supplementary Figure 14. Comparison of the fluorescence spectra of the enzymatically prepared 5'-end-TAMRA-labelled GFP-siRNA and a commercial 5'-end-TAMRA-labelled RNA produced by chemical synthesis, TAMRA-R-21 (Supplementary Table S1). 1: fluorescence spectrum (excitation wavelength: 250 nm; emission wavelength: 290 - 750 nm) of the commercial 5'-end-TAMRA-labelled RNA produced by chemical synthesis, TAMRA-R-21; 2: fluorescence spectrum (excitation wavelength: 250 nm; emission wavelength: 290 - 750 nm) of the enzymatically prepared 5'-end-TAMRA-labelled GFP-siRNA. Source data are provided in the Source Data file.

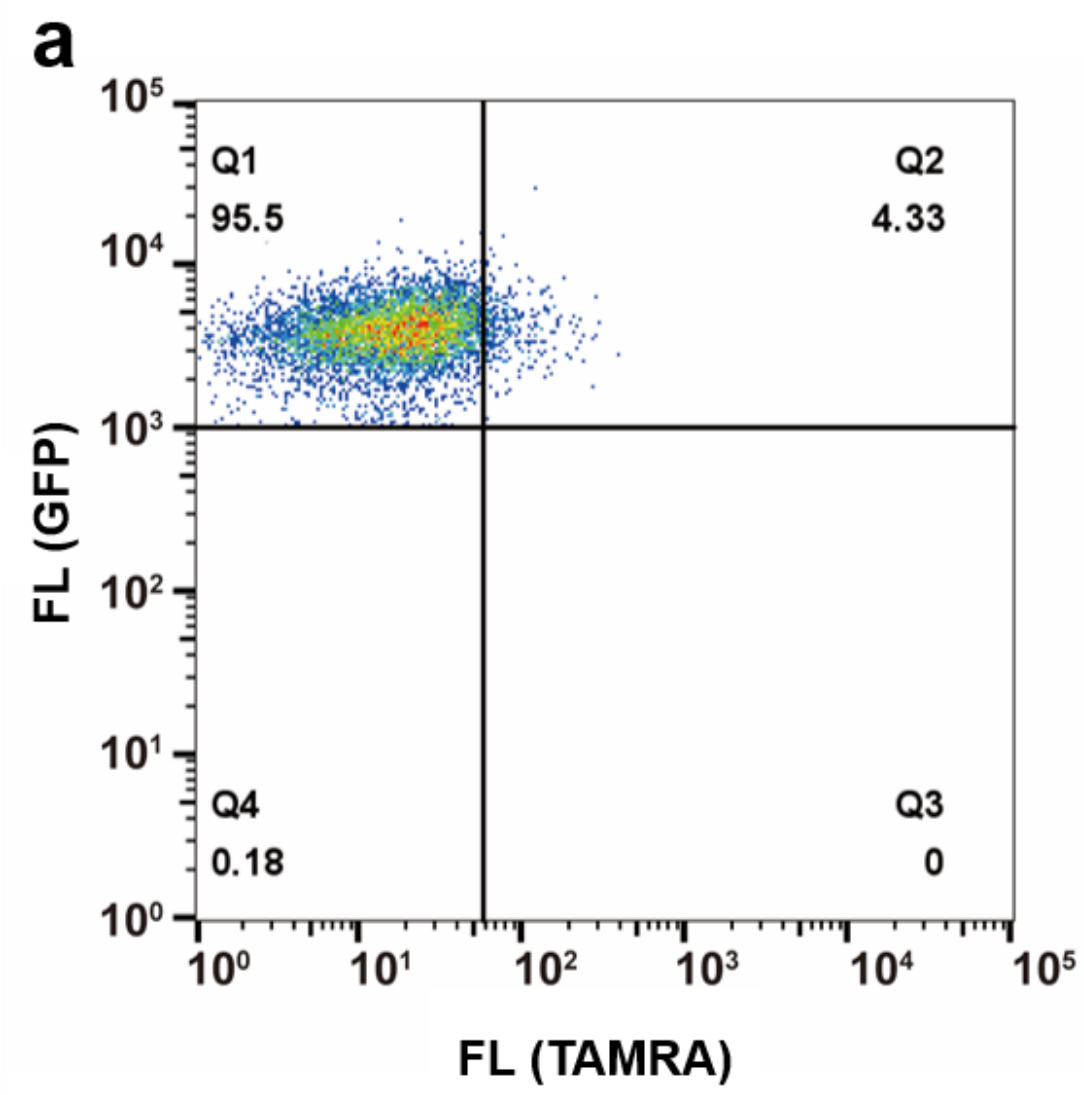

Supplementary Figure 15. Flow cytometry analysis for the *in vitro* interference experiments using the 5'-end-TAMRA-labelled siRNA prepared by SFM4-6. (**a**) B16(OVA)-GFP cells without interference with 5'-end-TAMRA-labelled siRNA. Q1 (UL): $GFP^{+}TAMRA^{-}$, 95.5%; Q2 (UR): $GFP^{+}TAMRA^{+}$, 4.33%; Q3 (LR): $GFP^{-}TAMRA^{+}$, 0%; Q4 (LL): $GFP^{-}TAMRA^{-}$, 0.18%. (**b**) B16(OVA)-GFP cells interfered with 5'-end-TAMRA-labelled siRNA. Q1 (UL): $GFP^{+}TAMRA^{-}$, 0.13%; Q2 (UR): $GFP^{+}TAMRA^{+}$, 2.51%; Q3 (LR): $GFP^{-}TAMRA^{+}$, 95.8%; Q4 (LL): $GFP^{-}TAMRA^{-}$, 1.57%. Source data are provided in the Source Data file.

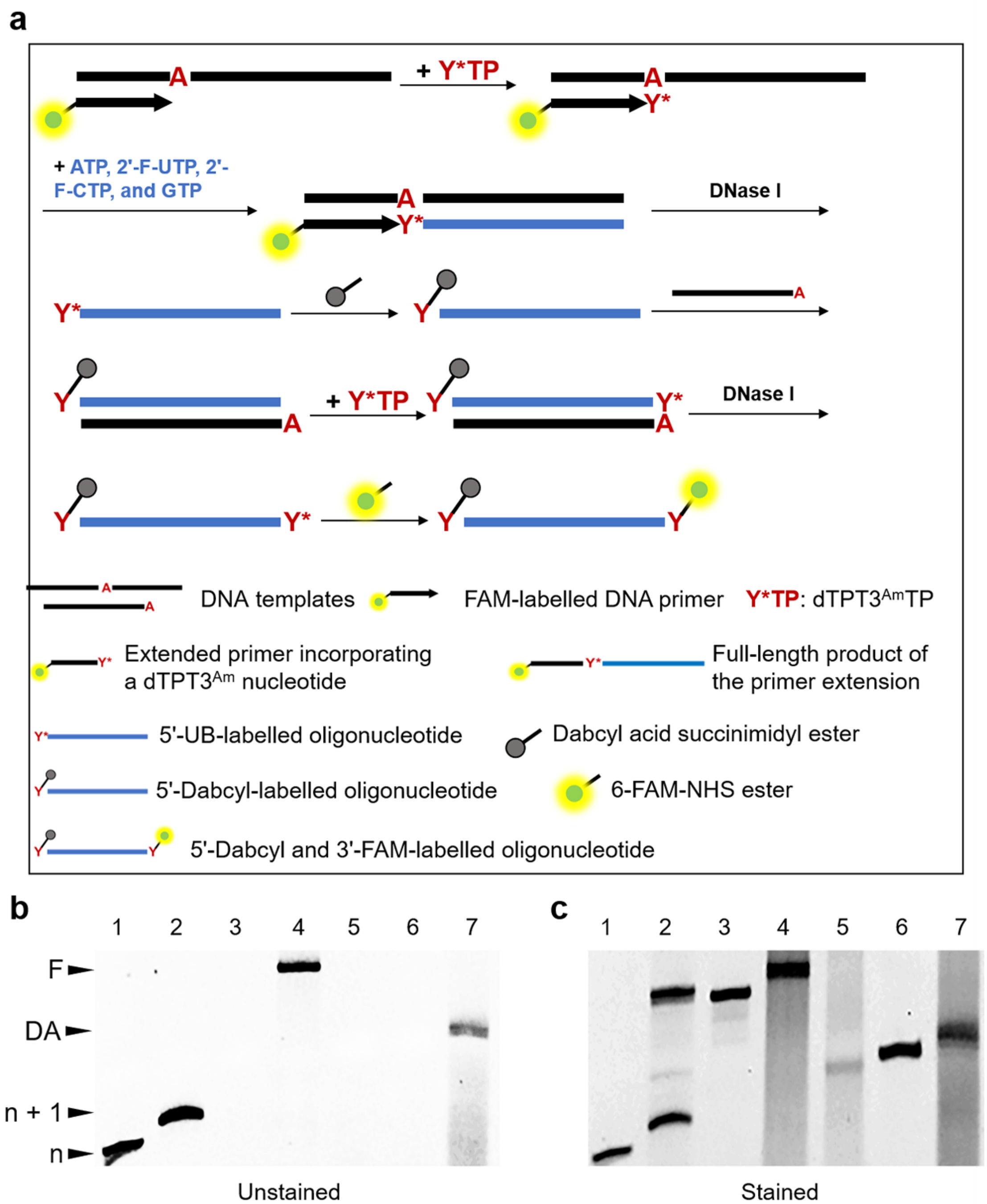

Supplementary Figure 16. Preparation of the 5'-end and 3'-end-dual-labelled 2'-F-CU-RNA aptasensor. (**a**) Schematic diagram for the preparation of the 5'-end and 3'-end-dual-labelled 2'-F-CU-RNA aptasensor. **(b-c**) Gel analysis of the intermediate and final products for the preparation of the aptasensor. The products were analyzed with 20% denaturing PAGE gel containing 8 M urea, and the gel was visualized before (**b**) and after (**c**) staining with Cyber Gold. Lane 1: FAM-labelled DNA primer FAM-P-20; lane 2: FAM-P-20 extended by the incorporation of a dTPT3^{Am} nucleotide opposite a dA

nucleotide in the DNA template, T46-thr; lane 3: DNA template T46-thr; lane 4: primer extension with ATP, 2'-F-UTP, 2'-F-CTP, and GTP after the incorporation of a $dTPT3^{Am}$ nucleotide opposite a dA nucleotide in the DNA template, T46-thr; lane 5: 5'-end-Dabcyl-labelled aptamer Dabcyl-Y-toggle-25t produced by DNase I degradation of the primer extension product and coupling the DNase I degradation product with Dabcyl acid succinimidyl ester; lane 6: DNA template T26-A; lane 7: 5'-end-Dabcyl and 3'-end-FAM-dual-labelled 2'-F-CU-RNA aptamer (aptasensor). n: position for primer FAM-P-20 (20 nt); n + 1: position for primer FAM-P-20 incorporating a $dTPT3^{Am}$ nucleotide (21 nt); DA: 5'-end and 3'-end-dual-labelled 2'-F-CU-RNA aptamer; F: full-length product of the primer extension. Source data are provided in the Source Data file.

a mGmAmAfUmGfCfCfCfUfCfUmAmGmGfCmAmAfCmAmAfUfUfUfCmAfC mAfCmAmGmGmAmAmAfCmAmG

Expected Mass: 12600.7; Observed Mass: :12598.6

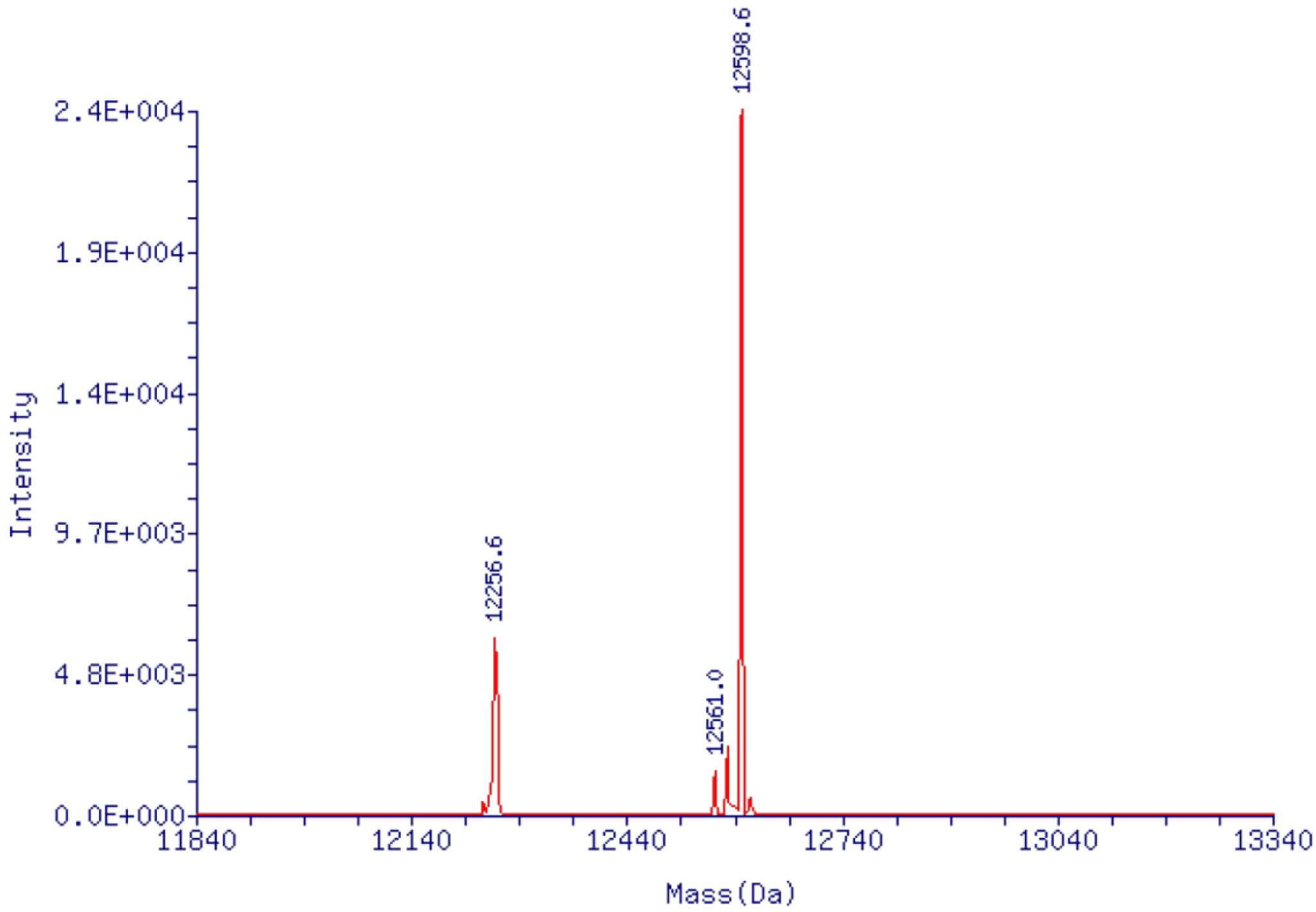

b mGmAmAfUmGfCfCfCfUfCrTPT3^{Al}mAmGmGfCmAmAfCmAmAfUfUfUfCmAfC mAfCmAmGmGmAmAmAfCmAmG

Expected Mass: 12678.7; Observed Mass: 12678.0

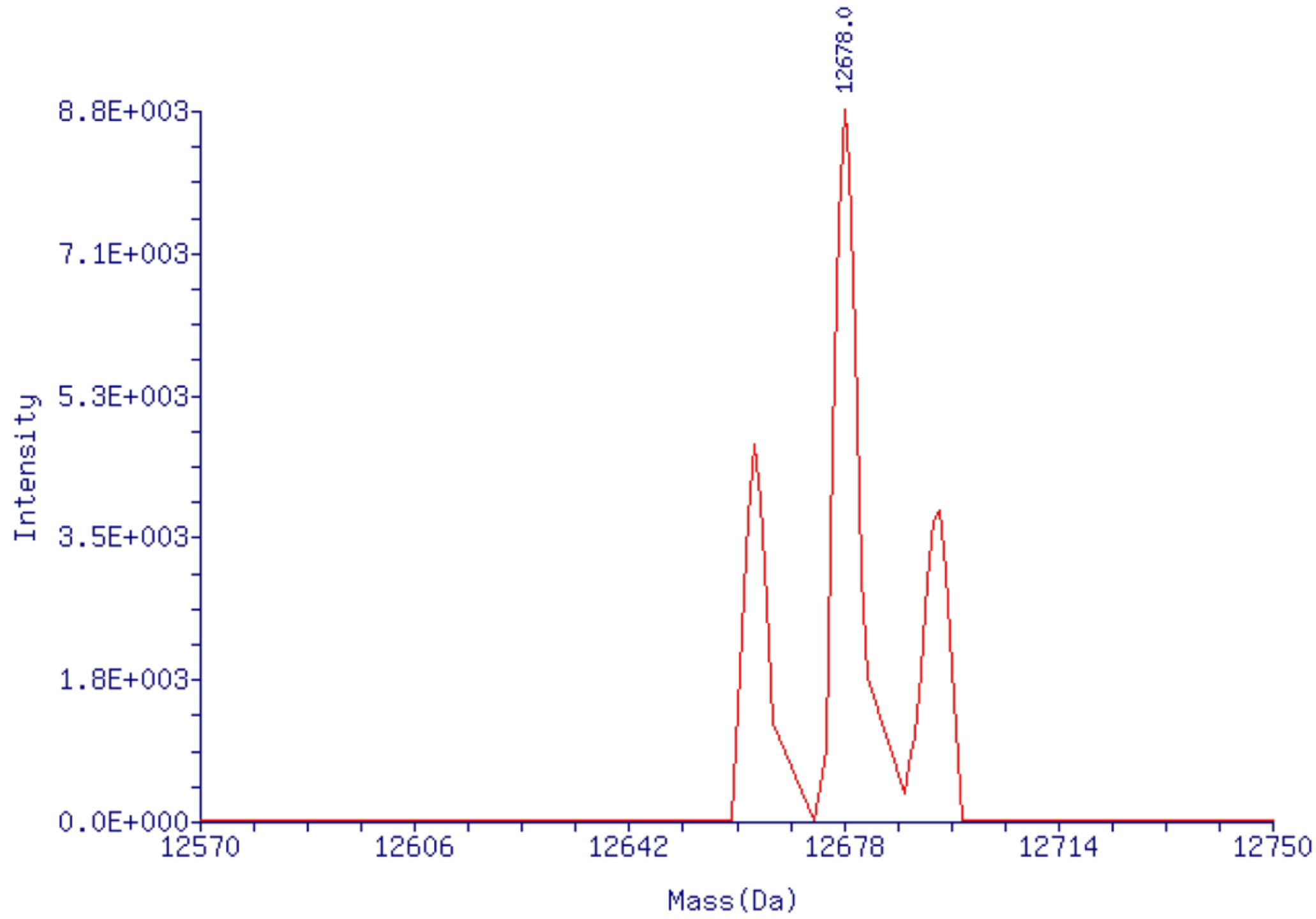

Supplementary Figure 17. Mass spectrometry analysis of the 2'-F/OMe-RNA

oligonucleotide products produced with dNaM-containing DNA template T55-X in the absence (**a**) and presence (**b**) of rTPT3^{Al}TP. In the absence of rTPT3^{Al}TP, a 2'-F-U nucleotide was incorporated opposite the dNaM in the template during the synthesis of the oligonucleotide, yielding a product with an expected mass of 12600.7 and an observed mass of 12598.6 (**a**). In the presence of rTPT3^{Al}TP, an rTPT3^{Al} nucleotide was correctly incorporated opposite the dNaM in the template during the synthesis of the oligonucleotide, yielding a product with an expected mass of 12678.7 and an observed mass of 12678.0 (**b**). The analyzed oligonucleotides in (**a**) and (**b**) correspond to the full-length primer extension products (F) in lanes 6 and 7 of Supplementary Figure 4a, respectively. The DNA primer in each product was removed by DNase I treatment before the mass spectrometry analysis. The source data are provided in the Source Data file.

a

mGmAmAmUmGmCmCmCmUmCmAmAmGmGmCmAmAmCmAmAmUmUmUmC
mAmCmAmCmAmGmGmAmAmAmCmAmG

Expected Mass: 12860.7; Observed Mass: 12859.6.

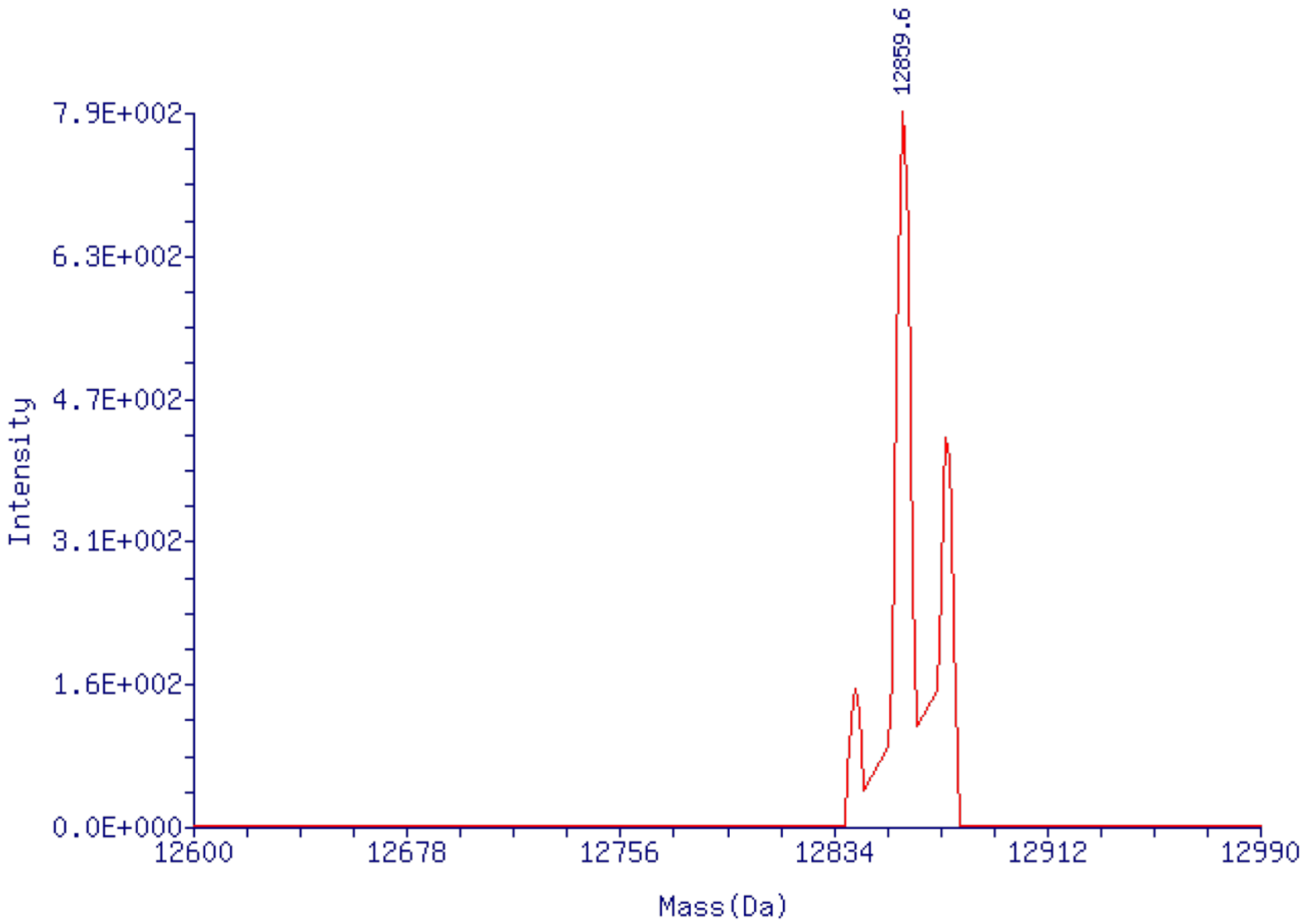

b

mGmAmAmUmGmCmCmCmUmCrTPT3^{Al}mAmGmGmCmAmAmCmAmAmUmUmUmC
mAmCmAmCmAmGmGmAmAmAmCmAmG

Expected Mass: 12901.7; Observed Mass: 12901.9

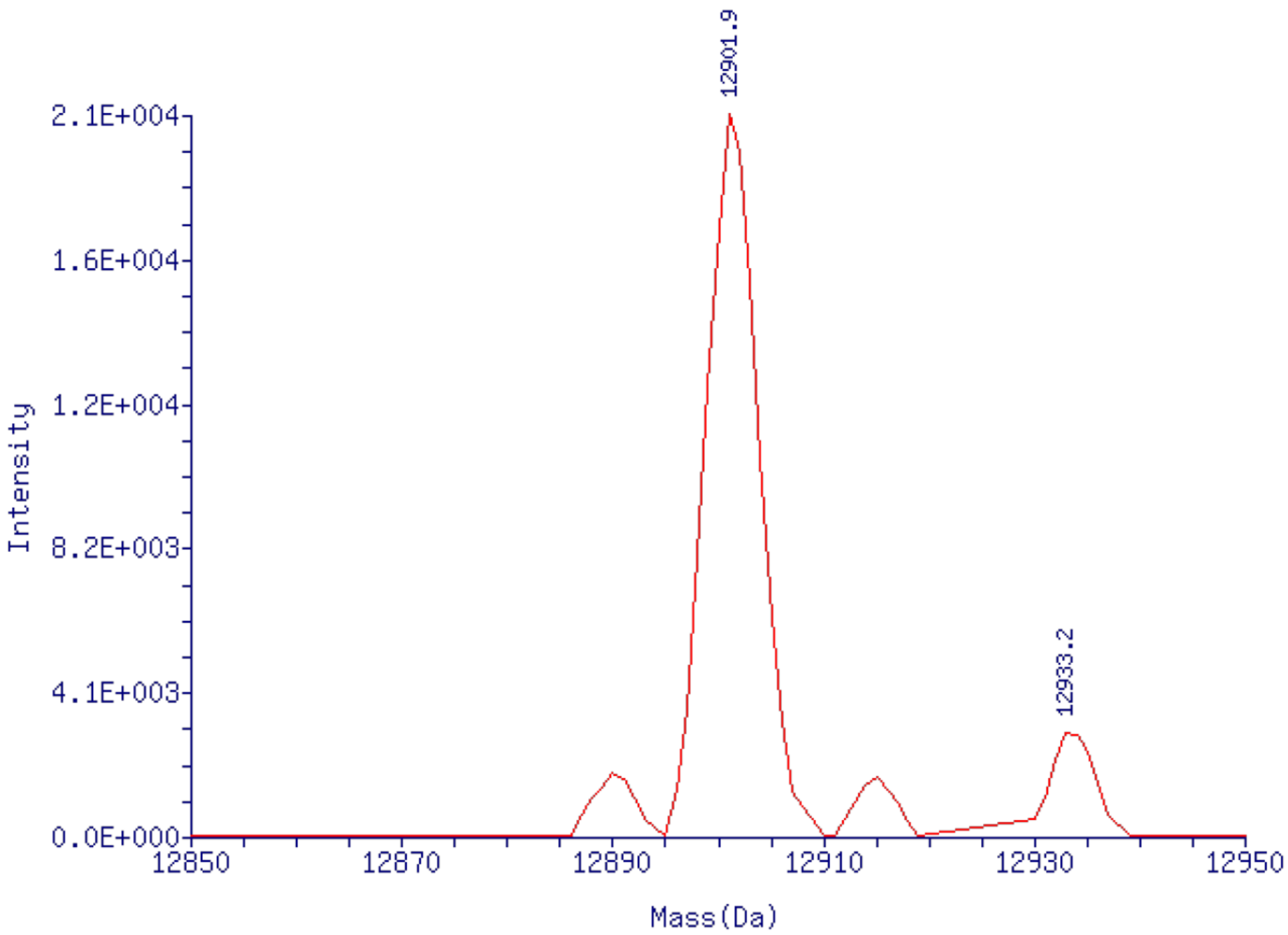

Supplementary Figure 18. Mass spectrometry analysis of the 2'-OMe-RNA oligonucleotide products produced with dNaM-containing DNA template T55-X in the absence (**a**) and presence (**b**) of rTPT3^{Al}TP. In the absence of rTPT3^{Al}TP, a 2'-OMe-A nucleotide was incorporated opposite the dNaM in the template, yielding a product with an expected mass of 12860.7 and an observed mass of 12859.6 (**a**). In the presence of rTPT3^{Al}TP, an rTPT3^{Al} nucleotide was correctly incorporated opposite the dNaM in the template during the synthesis of the oligonucleotide, yielding a product with an expected mass of 12901.7 and an observed mass of 12901.9 (**b**). The analyzed oligonucleotides in (**a**) and (**b**) correspond to the full-length primer extension products (F) in lanes 6 and 7 of Supplementary Figure 4b, respectively. The DNA primer in each product was removed by DNase I treatment before the mass spectrometry analysis. The source data are provided in the Source Data file.

5'-End-dTPT3^{Am}-labelled RNA

dTPT3^{Am} AGGCAACAAUUUCACACAGGAAACAG

Expected Mass: 9127.6; Observed Mass: 9127.2

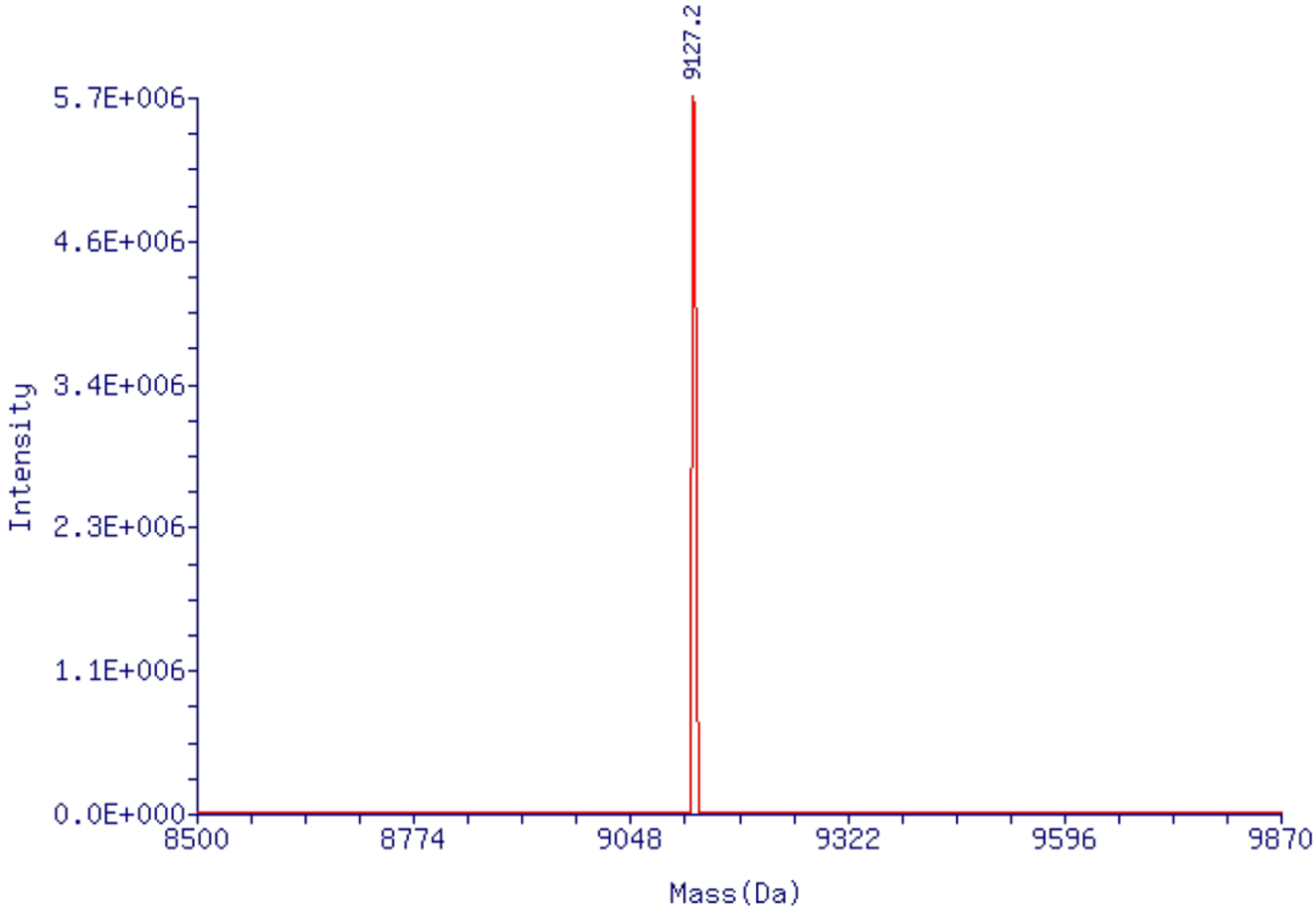

Supplementary Figure 19. Mass spectrometry analysis of the 5'-end-dTPT3^{Am}-labelled RNA oligonucleotide product. Expected mass: 9127.6; observed mass: 9127.2. The analyzed oligonucleotide corresponds to the DNase I-treated primer extension product (DP) in lane 2 of Supplementary Figure 12c. The source data is provided in the Source Data file.

5'-End-dTPT3^{AI}-labelled RNA

dTPT3^{AI} AGGCAACAAUUUCACACAGGAAACAG

Expected Mass: 9098.6; Observed Mass: 9098.2

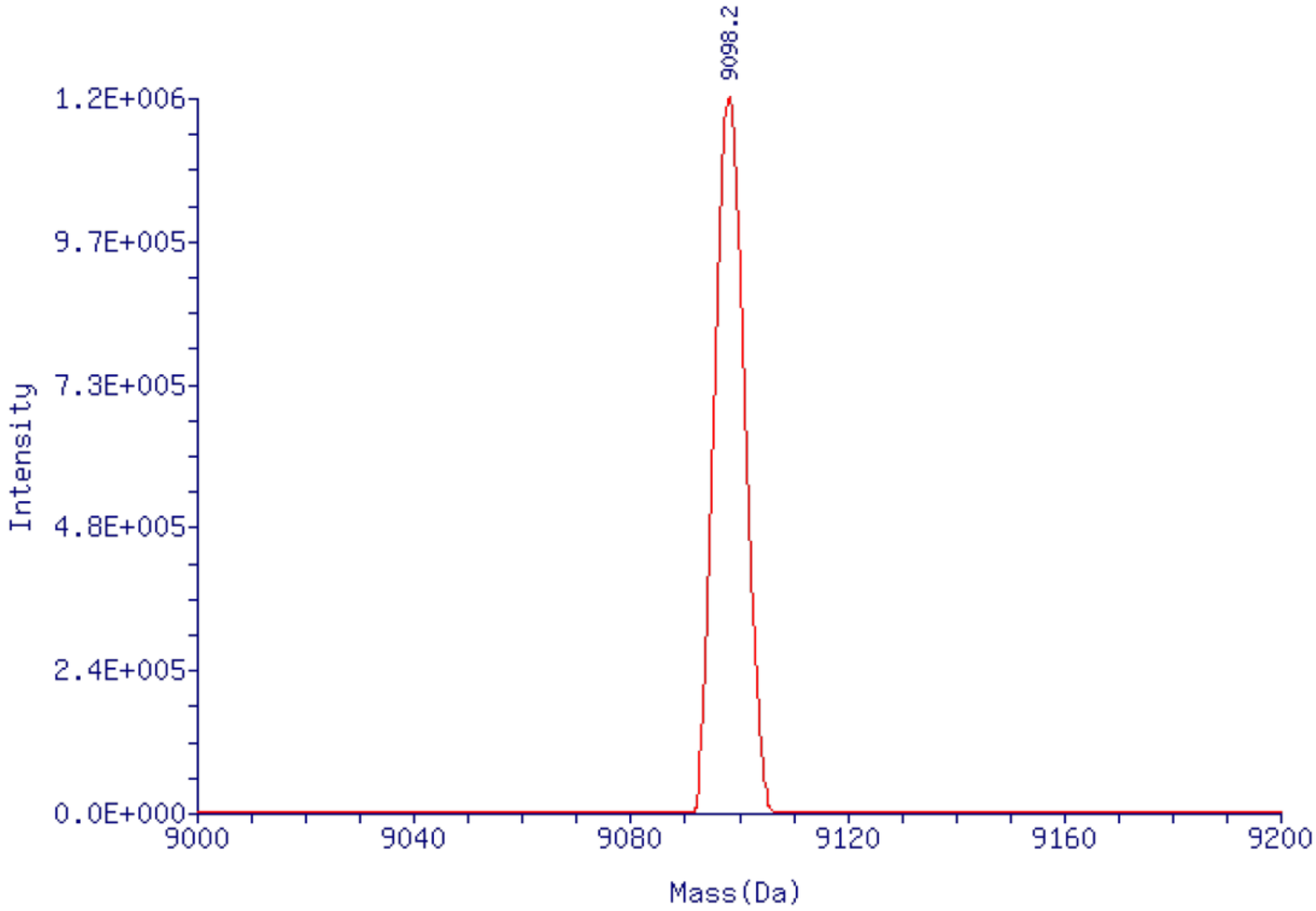

Supplementary Figure 20. Mass spectrometry analysis of the 5'-end-dTPT3^{AI}-labelled RNA oligonucleotide product. Expected mass: 9098.6; observed mass: 9098.2. The analyzed oligonucleotide corresponds to the DNase I-treated primer extension product (DP) in lane 2 of Supplementary Figure 12e. The source data is provided in the Source Data file.

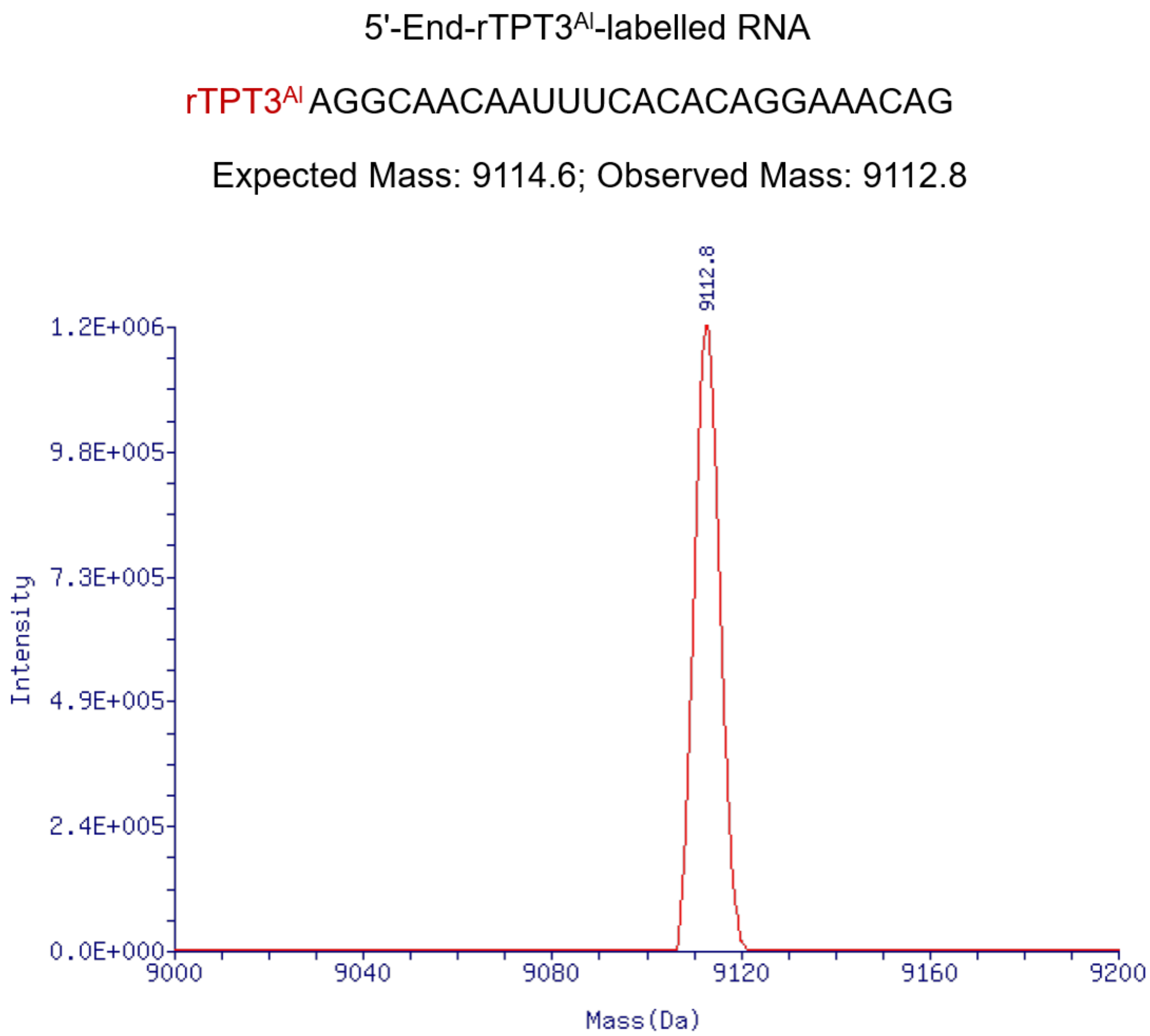

Supplementary Figure 21. Mass spectrometry analysis of the 5'-end-rTPT3^{AI}-labelled RNA oligonucleotide product. Expected mass: 9114.6; observed mass: 9112.8. The analyzed oligonucleotide corresponds to the DNase I-treated primer extension product (DP) in lane 2 of Supplementary Figure 12g. The source data is provided in the Source Data file.

5'-End-dTPT3^{Am}-labelled 2'-F-CU-RNA

dTPT3^{Am}AGGfCAAfCAAfUfUfUfCAfCAfCAGGAAAfCAG

Expected Mass: 9147.7; Observed Mass: 9147.3

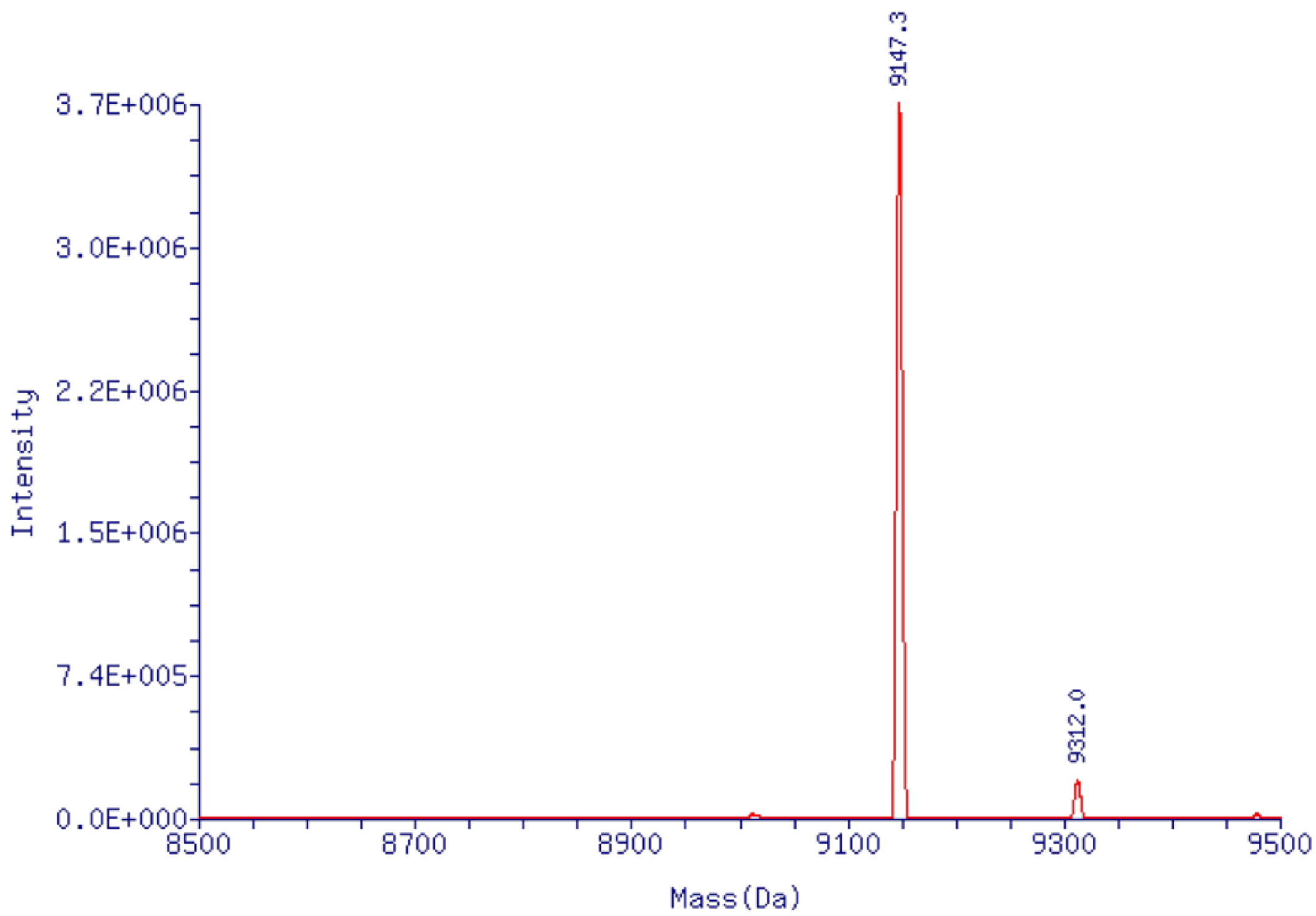

Supplementary Figure 22. Mass spectrometry analysis of the 5'-end-dTPT3^{Am}-labelled 2'-F-CU-RNA oligonucleotide product. Expected mass: 9147.7; observed mass: 9147.3. The analyzed oligonucleotide corresponds to the DNase I-treated primer extension product (DP) in lane 5 of Supplementary Figure 12c. The source data is provided in the Source Data file.

5'-End-dTPT3^{Al}-labelled 2'-F-CU-RNA

dTPT3^{Al}AGGfCAAfCAAfUfUfUfCAfCAfCAGGAAAfCAG

Expected Mass: 9117.7; Observed Mass: 9117.1

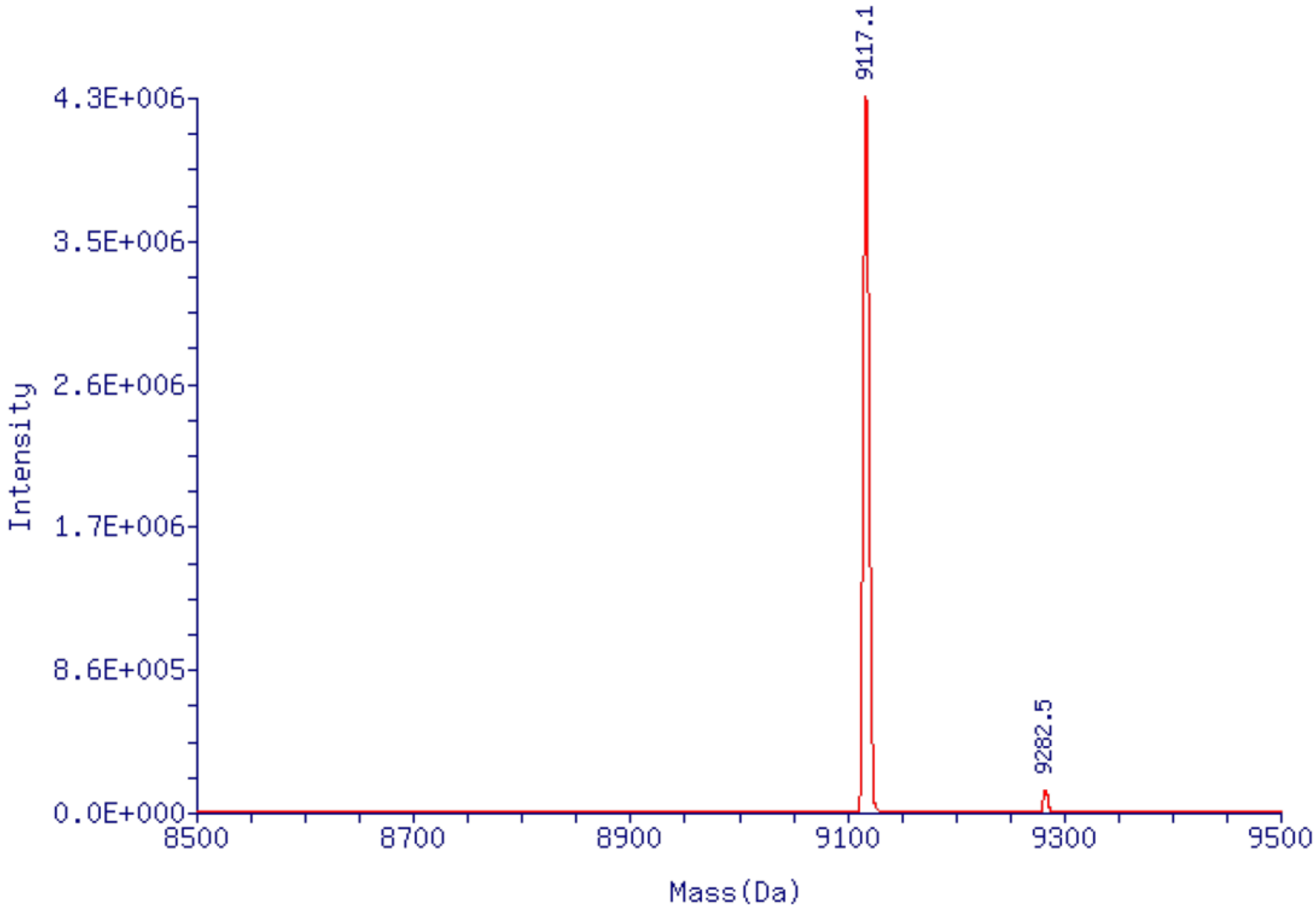

Supplementary Figure 23. Mass spectrometry analysis of the 5'-end-dTPT3^{Al}-labelled 2'-F-CU-RNA oligonucleotide product. Expected mass: 9117.7; observed mass: 9117.1. The analyzed oligonucleotide corresponds to the DNase I-treated primer extension product (DP) in lane 5 of Supplementary Figure 12e. The source data is provided in the Source Data file.

5'-End-rTPT3^{Al}-labelled 2'-F-CU-RNA

rTPT3^{Al}AGGfCAAfCAAfUfUfUfCAfCAfCAGGAAAfCAG

Expected Mass: 9133.7; Observed Mass: 9133.0

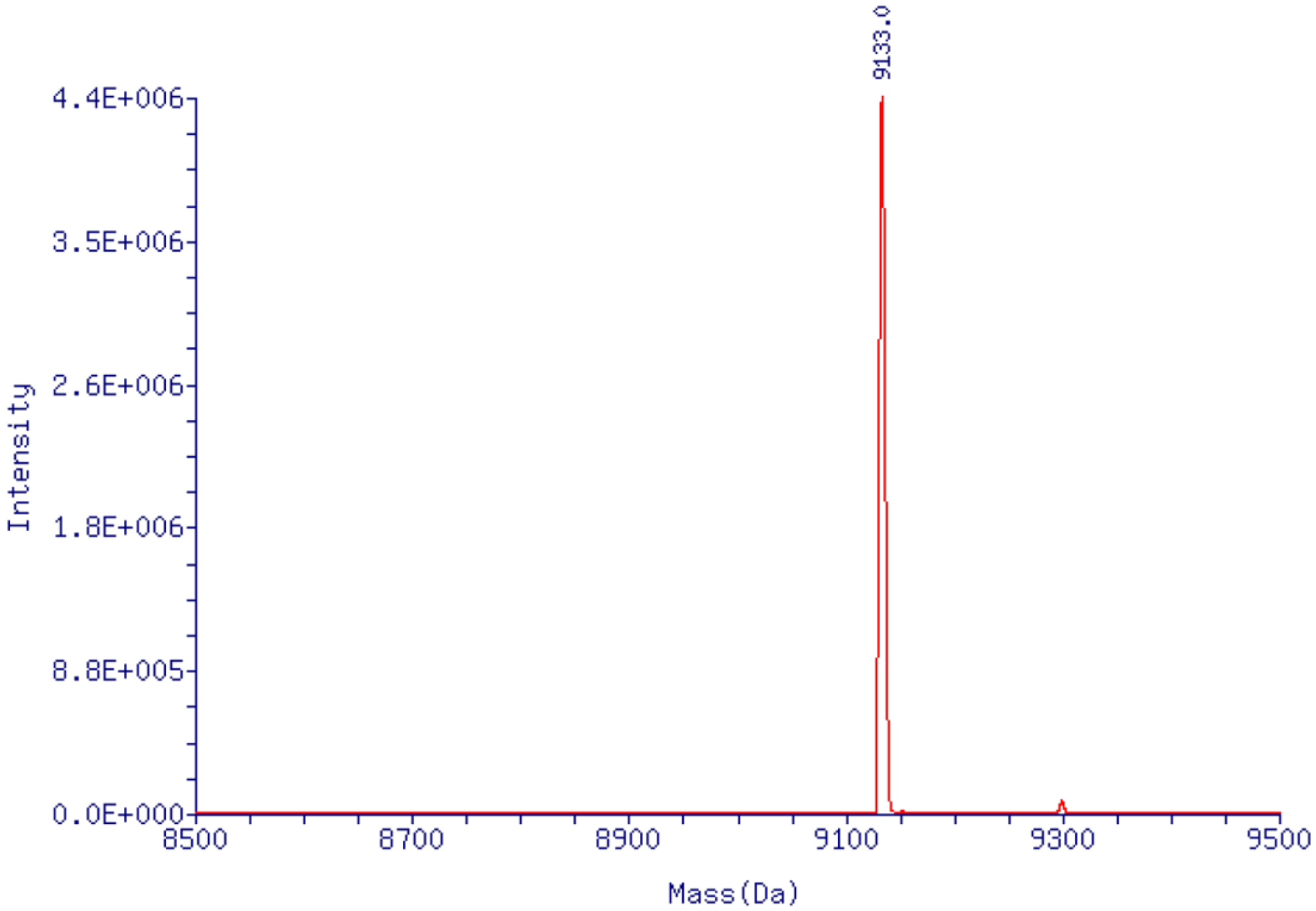

Supplementary Figure 24. Mass spectrometry analysis of the 5'-end-rTPT3^{Al}-labelled 2'-F-CU-RNA oligonucleotide product. Expected mass: 9133.6; observed mass: 9133.0. The analyzed oligonucleotide corresponds to the DNase I-treated primer extension product (DP) in lane 5 of Supplementary Figure 12g. The source data is provided in the Source Data file.

5'-End-dTPT3^{Am}-labelled 2'-F/OMe-RNA

dTPT3^{Am}mAmGmGfCmAmAfCmAmAfUfUfUfCmAfCmAfCmAmGmGmAmAmAfCmAmG

Expected Mass: 9386.6; Observed Mass: 9386.5

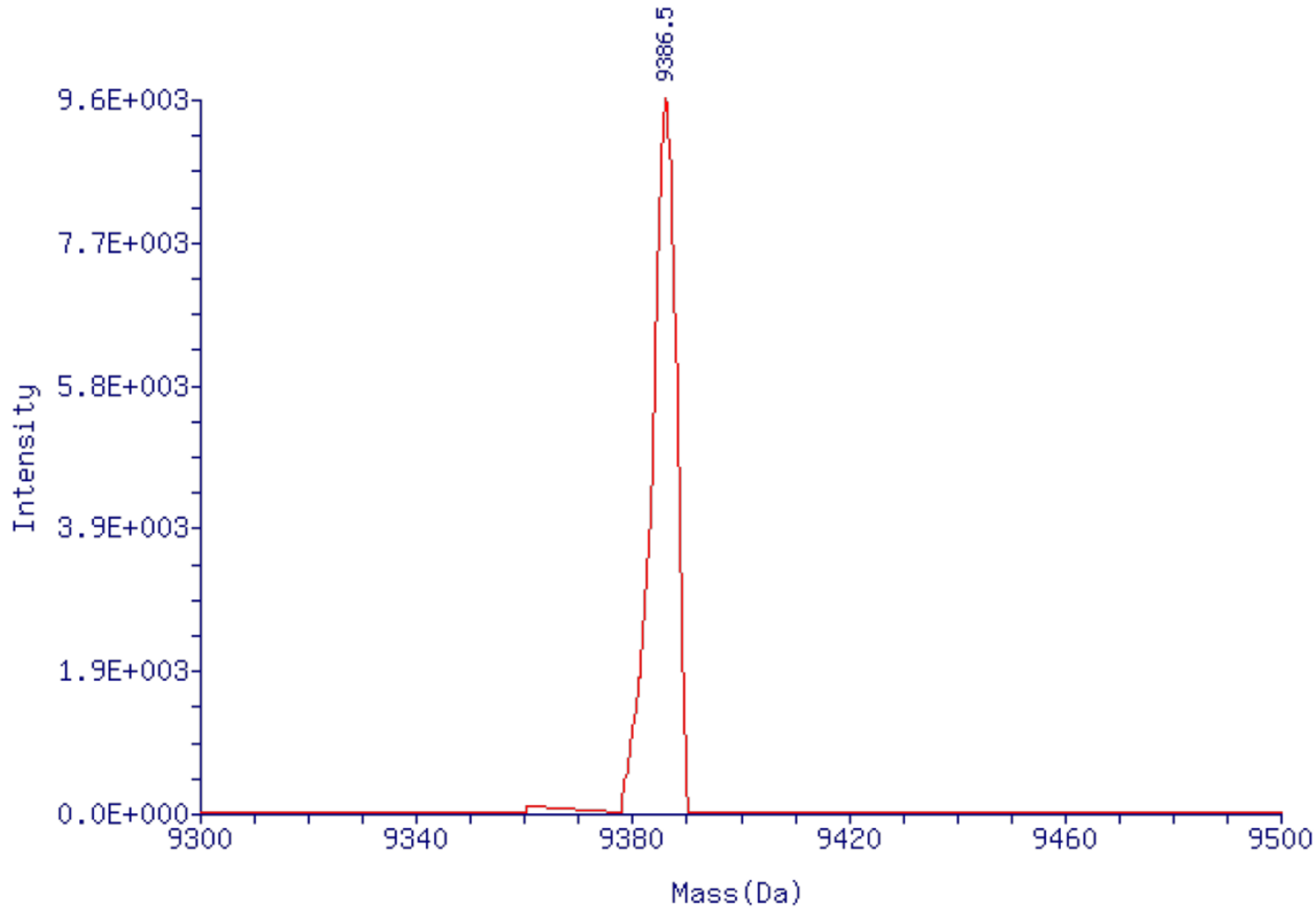

Supplementary Figure 25. Mass spectrometry analysis of the 5'-end-dTPT3^{Am}-labelled 2'-F/OMe-RNA oligonucleotide product. Expected mass: 9386.6; observed mass: 9386.5. The analyzed oligonucleotide corresponds to the DNase I-treated primer extension product (DP) in lane 8 of Supplementary Figure 12c. The source data is provided in the Source Data file.

5'-End-dTPT3^{AI}-labelled 2'-F/OMe-RNA

dTPT3^{AI}mAmGmGfCmAmAfCmAmAfUfUfUfCmAfCmAfCmAmGmGmAmAmAfCmAmG

Expected Mass: 9358.6; Observed Mass: 9359.6

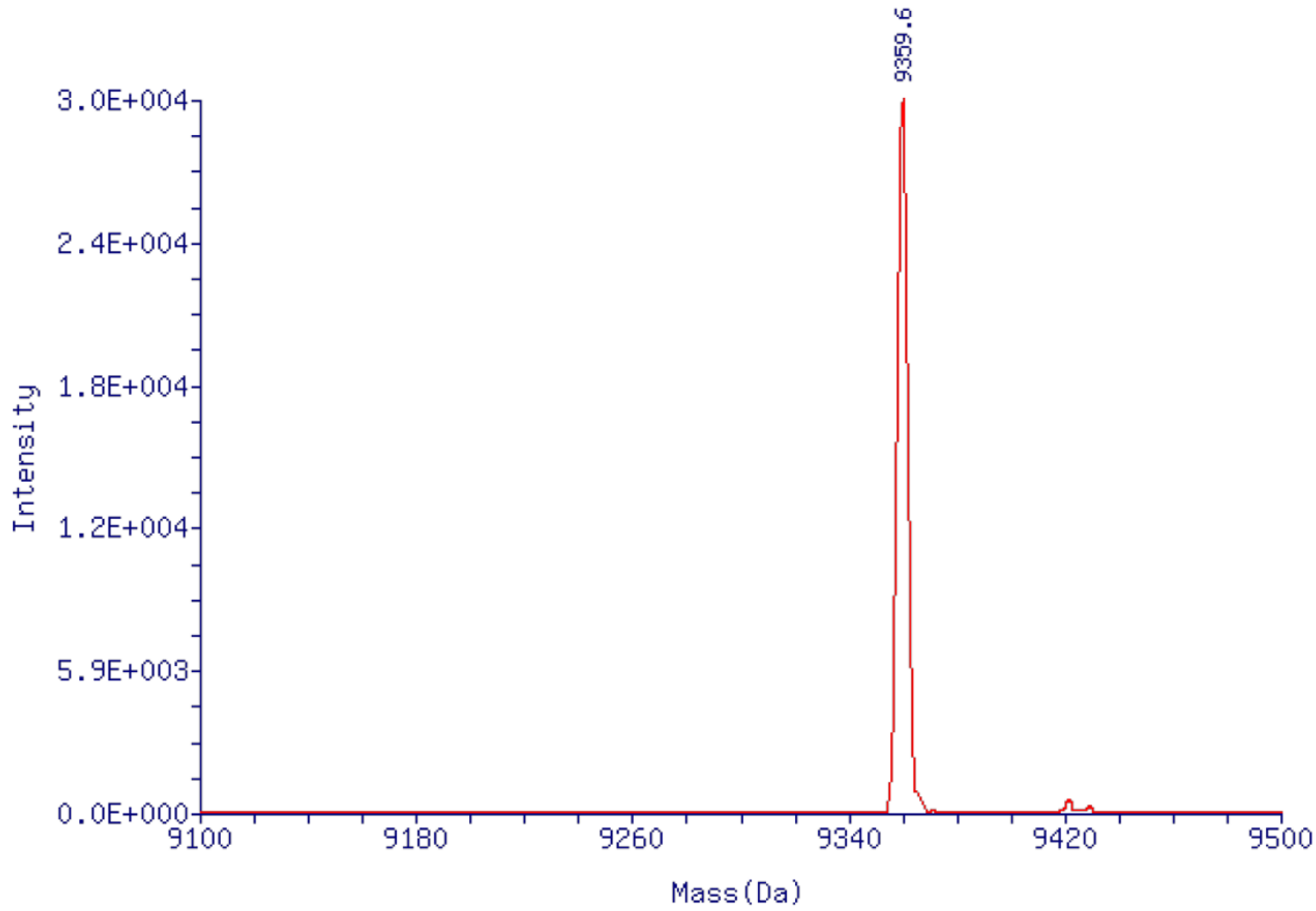

Supplementary Figure 26. Mass spectrometry analysis of the 5'-end-dTPT3^{AI}-labelled 2'-F/OMe-RNA oligonucleotide product. Expected mass: 9358.6; observed mass: 9359.6. The analyzed oligonucleotide corresponds to the DNase I-treated primer extension product (DP) in lane 8 of Supplementary Figure 12e. The source data is provided in the Source Data file.

5'-End-rTPT3^{Al}-labelled 2'-F/OMe-RNA

rTPT3^{Al}mAmGmGfCmAmAfCmAmAfUfUfUfCmAfCmAfCmAmGmGmAmAmAfCmAmG

Expected Mass: 9373.6; Observed Mass: 9373.7

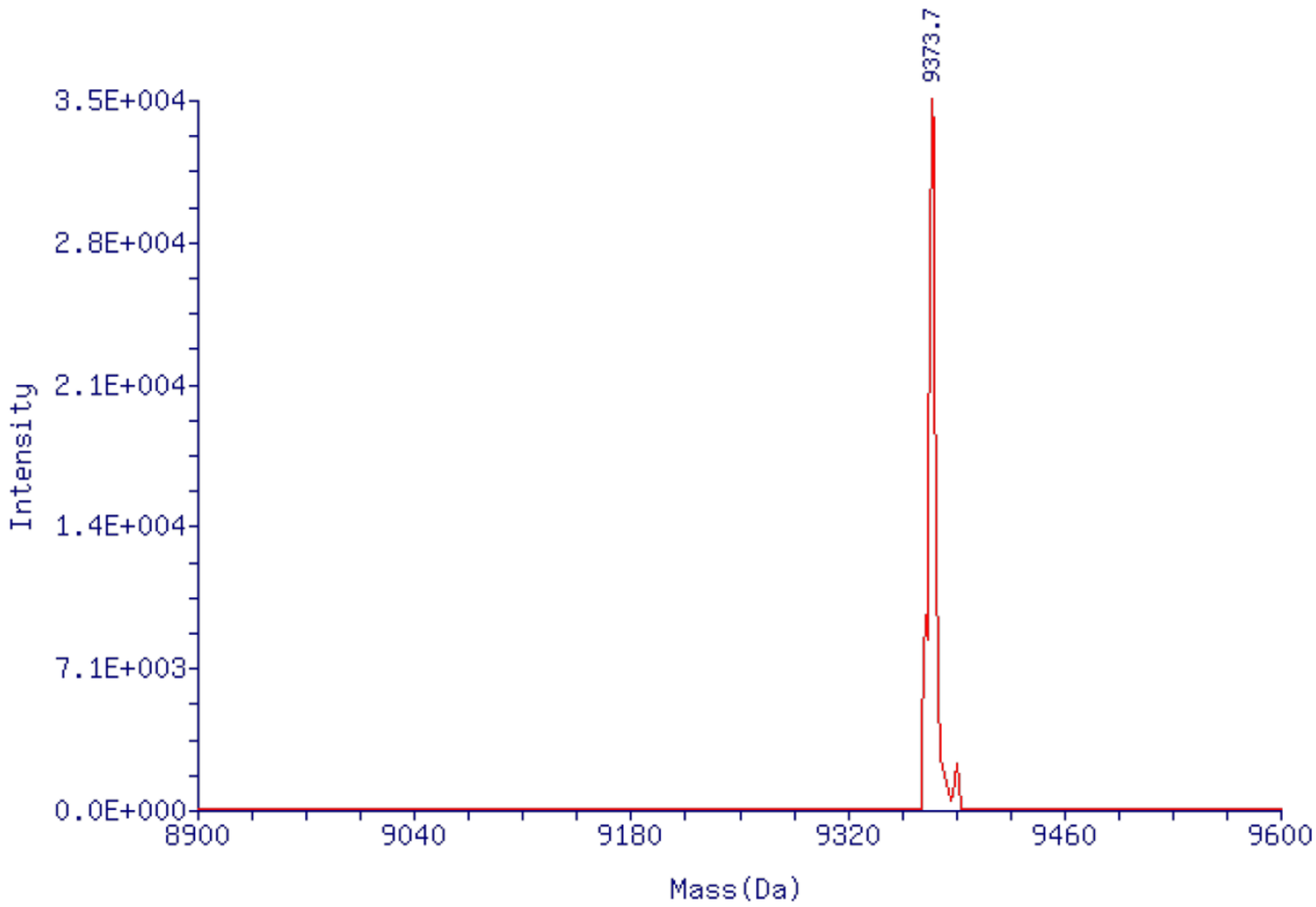

Supplementary Figure 27. Mass spectrometry analysis of the 5'-end-rTPT3^{Al}-labelled 2'-F/OMe-RNA oligonucleotide product. Expected mass: 9373.6; observed mass: 9373.7. The analyzed oligonucleotide corresponds to the DNase I-treated primer extension product (DP) in lane 8 of Supplementary Figure 12g. The source data is provided in the Source Data file.

5'-End-dTPT3^{Am}-labelled 2'-OMe-RNA

dTPT3^{Am}mAmGmGmCmAmAmCmAmAmUmUmUmCmAmCmAmCmAmGmGmAmAmAmCmAmG

Expected Mass: 9497.3; Observed Mass: 9498.6

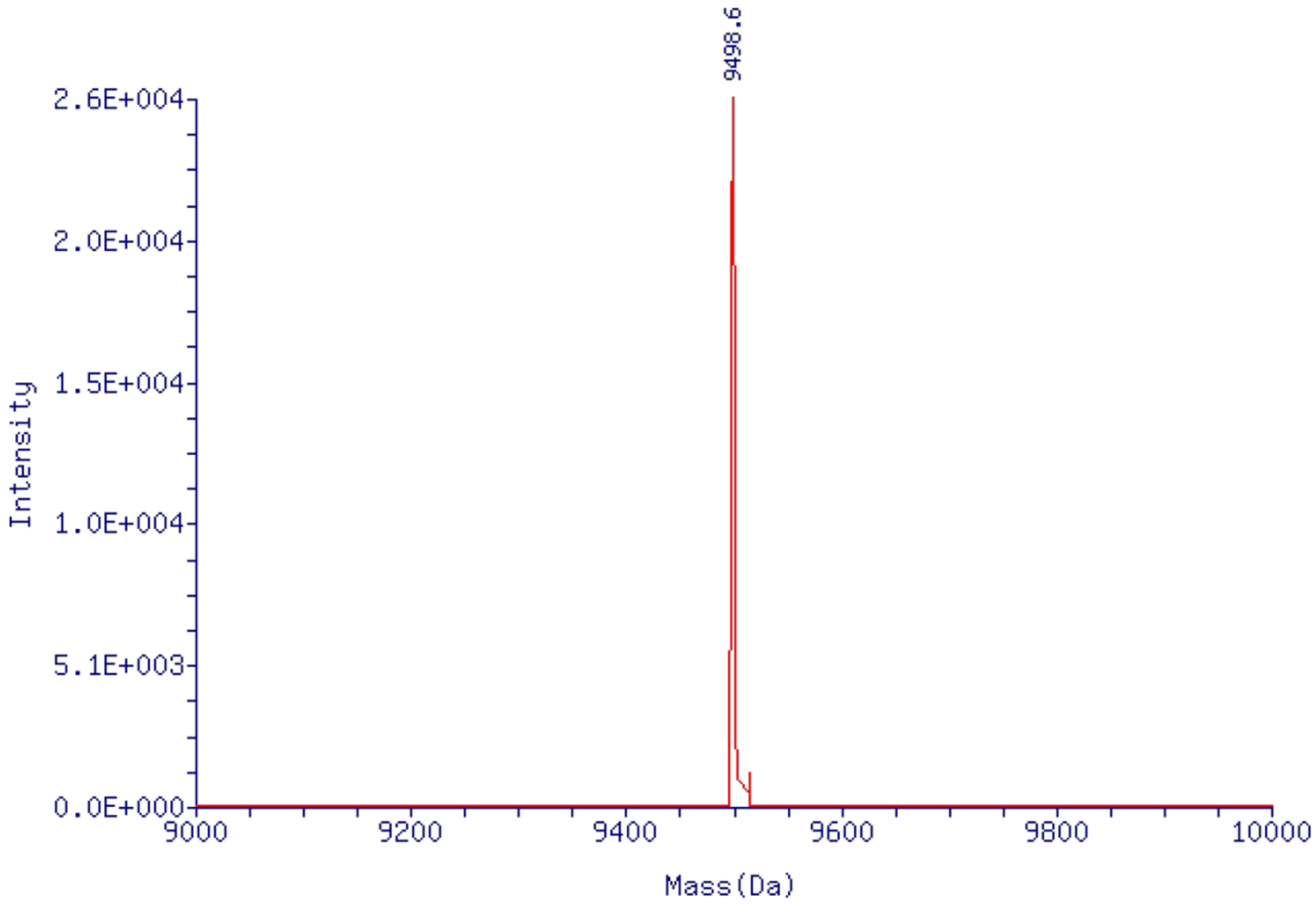

Supplementary Figure 28. Mass spectrometry analysis of the 5'-end-dTPT3^{Am}-labelled 2'-OMe-RNA oligonucleotide product. Expected mass: 9497.3. Observed mass: 9498.6. The analyzed oligonucleotide corresponds to the DNase I-treated primer extension product (DP) in lane 11 of Supplementary Figure 12c. The source data is provided in the Source Data file.

5'-End-dTPT3^{Al}-labelled 2'-OMe-RNA

dTPT3^{Al}mAmGmGmCmAmAmCmAmAmUmUmUmCmAmCmAmCmAmGmGmAmAmAmCmAmG

Expected Mass: 9467.3; Observed Mass: 9466.2

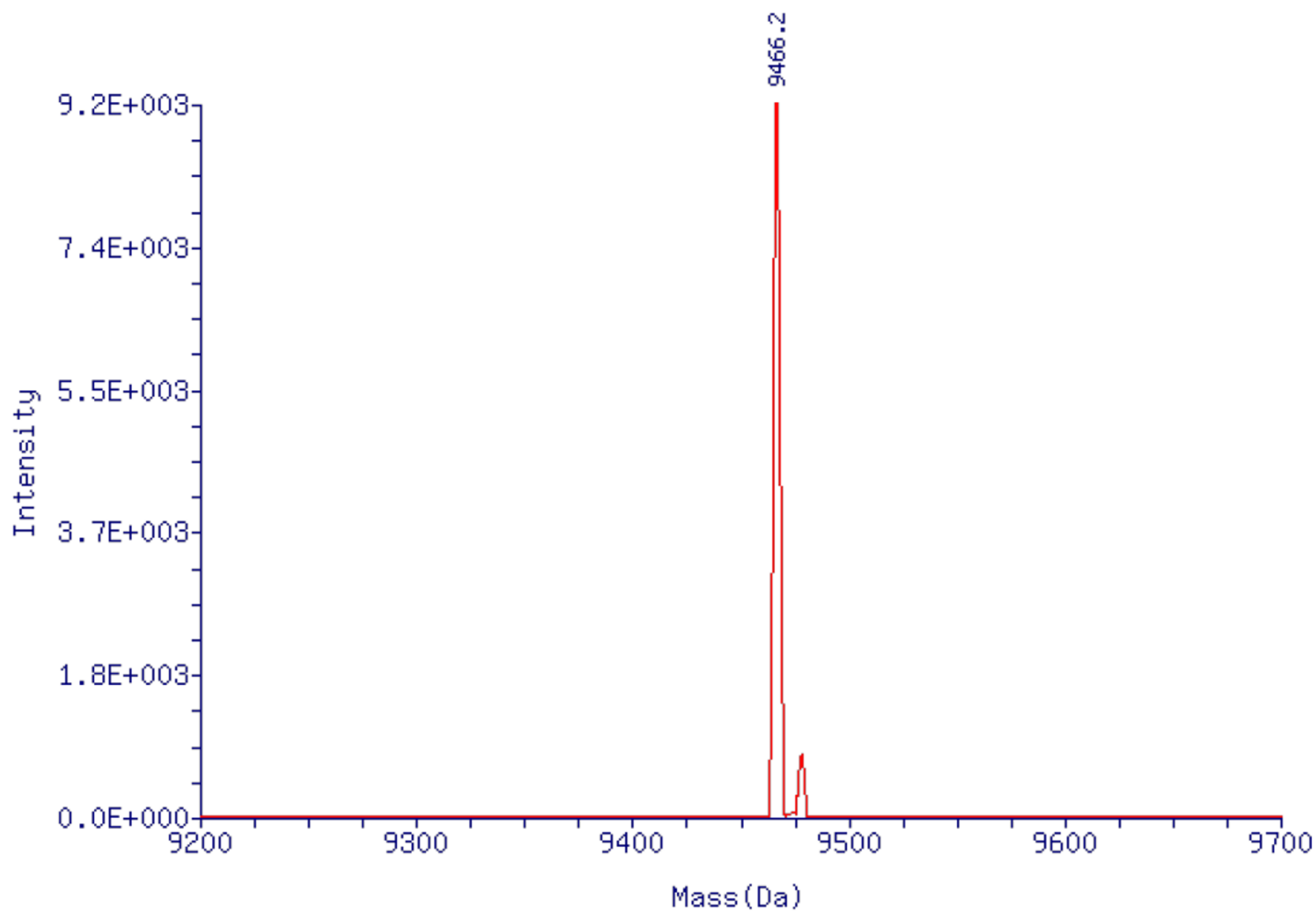

Supplementary Figure 29. Mass spectrometry analysis of the 5'-end-dTPT3^{Al}-labelled 2'-OMe-RNA oligonucleotide product. Expected mass: 9467.3; observed mass: 9466.2. The analyzed oligonucleotide corresponds to the DNase I-treated primer extension product (DP) in lane 11 of Supplementary Figure 12e. The source data is provided in the Source Data file.

5'-End-rTPT3^{Al}-labelled 2'-OMe-RNA

rTPT3^{Al}mAmGmGmCmAmAmCmAmAmUmUmUmCmAmCmAmCmAmGmGmAmAmAmCmAmG

Expected Mass: 9485.3; Observed Mass: 9486.2

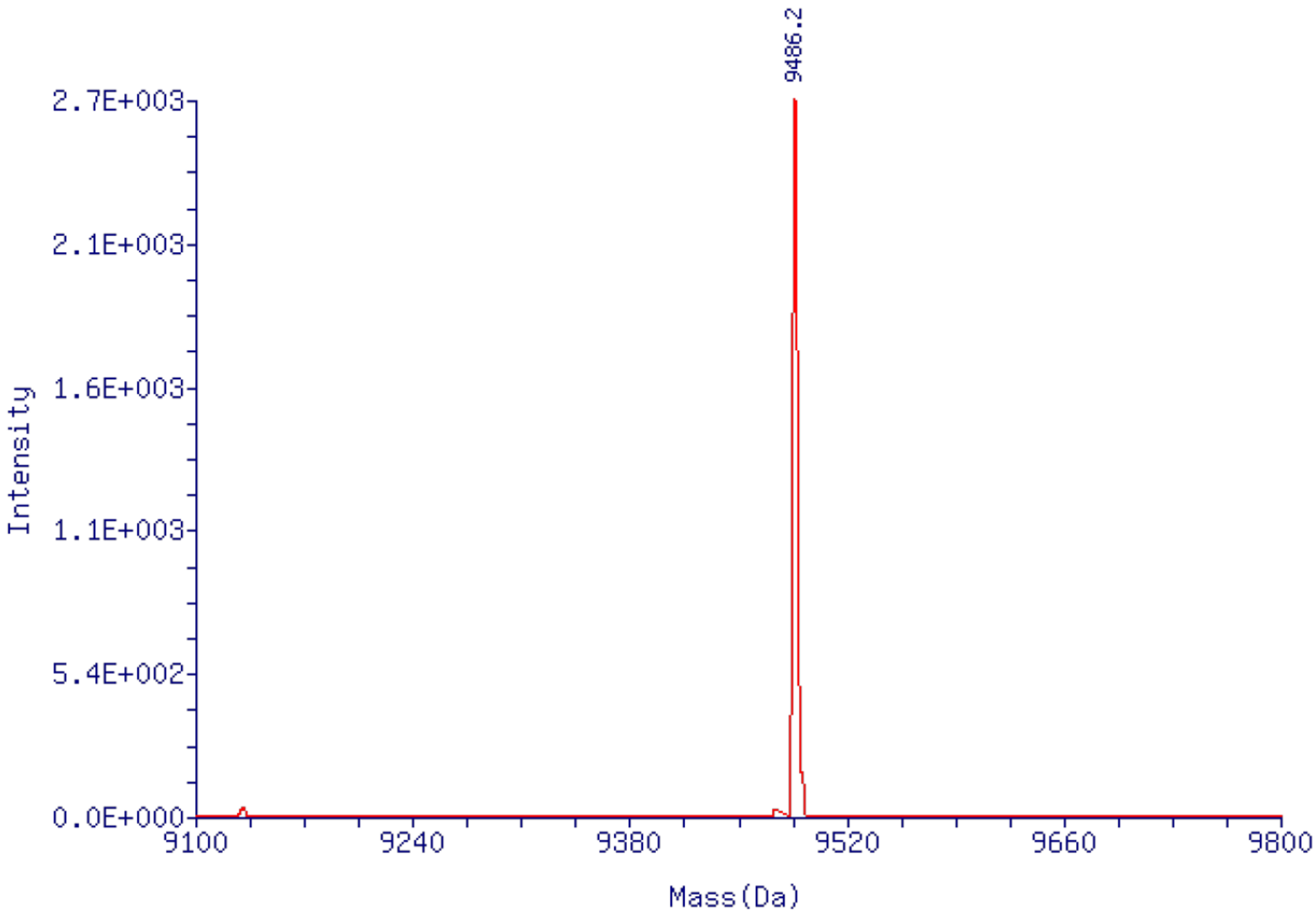

Supplementary Figure 30. Mass spectrometry analysis of the 5'-end-rTPT3^{Al}-labelled 2'-OMe-RNA oligonucleotide product. Expected mass: 9485.3; observed mass: 9486.2. The analyzed oligonucleotide corresponds to the DNase I-treated primer extension product (DP) in lane 11 of Supplementary Figure 12g. The source data is provided in the Source Data file.

Supplementary Tables

Supplementary Table 1. Oligonucleotides used in this work.

Name	Sequence (5' - 3')
T55-X	CTGTTTCCTGTGTGAAATTGTTGCCTXGAGGGCATTCCACACAACA TACGAAGCC
FAM-P-20	FAM-ATGTTGTGTGGAATGCCCTC
FAM-P-18	FAM-GGCTTCGTATGTTGTGTG
T55-A	CTGTTTCCTGTGTGAAATTGTTGCCATGAGGGCATTCCACACAACA TACGAAGCC
T55-T	CTGTTTCCTGTGTGAAATTGTTGCCTTGAGGGCATTCCACACAACA TACGAAGCC
T55-C	CTGTTTCCTGTGTGAAATTGTTGCCCTGAGGGCATTCCACACAACA TACGAAGCC
T55-G	CTGTTTCCTGTGTGAAATTGTTGCCGTGAGGGCATTCCACACAACA TACGAAGCC
T46-thr	GGGTAAGTACTTCAGCTTTGTTCCCAGAGGGCATTCCACACAACAT
T42-sense	ATAGACGTTGTGGCTGTTGTATGAGGGCATTCCACACAACAT
T42-anti	TACAACAGCCACAACGTCTATGGAGGGCATTCCACACAACAT
T90-1	ACTGGGACTGTGTGAAACCGATGGGGGTAATGATACCGATGAAAC GAGAGCGCATTGATAGCATAGAGATCCGTCATAAAGTGTAAAGCC
T90-2	ACTGGGACTGTGTGAAACCGTCCGATTGGATAAACGAGTCCATGTA GCCACGCATTGATAGCATAGAGATCCGTCATAAAGTGTAAAGCC
T90-3	ACTGGGACTGTGTGAAACCGCTCGGAAACATTGGTACGATTTTTAG CCGCGCATTGATAGCATAGAGATCCGTCATAAAGTGTAAAGCC
FAM-P-19	FAM-GGCTTTACACTTTATGACG
T26-A	AGGGTAAGTACTTCAGCTTTGTTCCC
T26-T	TGGGTAAGTACTTCAGCTTTGTTCCC
T26-C	CGGGTAAGTACTTCAGCTTTGTTCCC
T26-G	GGGGTAAGTACTTCAGCTTTGTTCCC

FAM-R-25	FAM-rGrGrGrArArCrArArArGrCrUrGrArArGrUrArCrUrUrArCrCrC
FAM-f/m-25	FAM-mGmGmGmAmAfCmAmAmAmGfCffUmGmAmAmGfUmAfCfUfU mAfCfCfC
TAMRA-R-21	TAMRA-rUrArCrArArCrArGrCrCrArCrArArCrGrUrCrUrArU

*X: dNaM; r: ribonucleotide; f: 2'-F-modified nucleotide; m: 2'-OMe-modified nucleotide; FAM: FAM-labelled.

Supplementary Table 2. Steady-state kinetic constants for SFM4-6-mediated incorporation of different unnatural and natural nucleoside triphosphates opposite a natural nucleotide (N or M) in the DNA template

3'-CCGAAGCATACAACACACCTTACGGGAGNTCCGTTGTTAAAGTGTGTCCTTTGTC-5'
5'-ATGTTGTGTGGAATGCCCTC

N	Nucleoside triphosphate	K_m (μM)	k_{cat} (min^{-1})	k_{cat}/K_m (mM^{-1} min^{-1})
A	dTPT3TP	1.76 ± 0.065	3.37 ± 0.11	1.91×10^3
T	dTPT3TP	1.62 ± 0.12	2.88 ± 0.12	1.78×10^3
C	dTPT3TP	3.41 ± 0.16	2.22 ± 0.055	0.65×10^3
G	dTPT3TP	3.19 ± 0.13	2.23 ± 0.052	0.70×10^3
A	rTPT3TP	2.11 ± 0.35	1.13 ± 0.071	0.54×10^3
T	rTPT3TP	2.23 ± 0.063	1.13 ± 0.10	0.51×10^3
C	rTPT3TP	3.60 ± 1.78	0.97 ± 0.14	0.27×10^3
G	rTPT3TP	2.40 ± 1.08	1.07 ± 0.10	0.45×10^3

3'-CCGAAGCATACAACACACCTTACGGGAGAMCCGTTGTTAAAGTGTGTCCTTTGTC-5'
5'-ATGTTGTGTGGAATGCCCTCY

M	Nucleoside triphosphate	K_m (μM)	k_{cat} (min-1)	k_{cat}/K_m (mM^{-1} min^{-1})
T	ATP	0.47 ± 0.11	19.69 ± 1.74	4.19×10^4
	2'-F-ATP	0.45 ± 0.15	26.44 ± 2.53	5.88×10^4
	2'-OMe-ATP	1.02 ± 0.28	5.22 ± 0.094	5.12×10^3
	dTPT3TP	3.36 ± 0.46	0.75 ± 0.10	0.22×10^3

*Y = TPT3

Supplementary Note

Supplementary Note 1. Protein sequence of polymerase SFM4-6 (the sequence of the his tag for protein purification is underlined).

MAQPASPKALEEAPWPPPEGAFVGFVLSRKEPMWADLLALAAARGGRVHRAPEPYKALR
DLKEARGLLAKDLSVLALREGLGLPPGDDPMLLAYLLDPSNTTPEGVARRYGGEWTEEA
GERAALSERLFANLWGRLEGEERLLWLYREVERPLSAVLAHMEATGVRLDVAYLRALSLE
VAEEIARLEAEVFRLAGHPFNLNSRDQLERVLFDELGLPAIGKTEKTGKRSTSAAVLEALR
EAHPIVEKILQYRELTKLKSTYIDPLPDLIHPRTGRLHTRFNQTATATGRLSSSDPNLQNIPV
RTPLGQRIRRAFIAEEGWLLVALDYSQEGLRVLAHLSGDENLIRVFQEGRDIHTETASWMF
GVPREAVNPMMRRAAKTINFGVLYGMSAHRLSQKLAIPYEEAQAFIERYFQSFPKVRAWI
EKTLEEGRRRGYVETLFGRRRYVPDLEARVKSVRNAAERRAFNMPVQGTAADLMKLAM
VKLFPRLEEMGARMLLQVHDELVLEAPKERAEAVARLAKEVMEGVYPLAVPLEVEVGIG
EDWLSAKEAAHHHHHH*
