# Supplementary material for: Engineered polymerase-mediated efficient synthesis of site-specifically functionalized RNA and 2’-modified RNA oligonucleotides via genetic alphabet expansion
Source: Nat Commun. 2026 Aug 13;17:9705. doi: 10.1038/s41467-026-76347-0 (PMC13562680; doi:10.1038/s41467-026-76347-0)
Supplement: Supplementary file 3 [file 41467_2026_76347_MOESM3_ESM.pdf]

nature portfolio

Peer Review File

Engineered polymerase-mediated efficient synthesis of site-specifically functionalized RNA and 2'-modified RNA oligonucleotides via genetic alphabet expansion

Corresponding Author: Professor Tingjian Chen

This file contains all reviewer reports in order by version, followed by all author rebuttals in order by version.

Version 0:

Reviewer comments:

Reviewer #1

(Remarks to the Author)
The manuscript by Wu et al describes the use of an expanded genetic code to access structurally complex oligonucleotides containing 2'-modifications and non-canonical nucleobases. The incorporation of dTPT3/dNaM nucleotides containing reactive handles enabled downstream bioconjugation reactions and access to fluorescent labelled aptamers, aptasensors and siRNAs. The use of UBPs to expand the structural complexity of oligonucleotides is an interesting approach. The authors have previously shown that a biotinylated dTPT3 analogue can be incorporated into oligonucleotide sequences, and other have shown the incorporation of dTPT3 into modified TNA sequences. This manuscript builds on these studies to generate 2'-modified oligonucleotides functionalized with reactive handles to enable covalent attachment to biophysical/fluorescent labels which have important therapeutic and diagnostic applications.

One limitation of the approach is the requirement for stoichiometric primer and DNA template, which must be produced (and purified/isolated) using either chemical or enzymatic methods. This raises the question 'Why not directly synthesize the target oligonucleotide?'. This should be explained in the introduction. Another limitation is that the polymerase efficiency in extension reactions of sequences containing dTPT3 nucleotides containing propargylamino or ethynyl modifications is severely compromised (Fig S3). Although the enzyme can extend dTPTAm and dTPTAl using rNTPs, the conversion to fully extended product using 2'-fluoro and/or methoxy is very low. This should also be discussed in the manuscript. Do the authors have an explanation for this and could they improve the efficiency with further engineering?

For the measurement of the kinetic constants, the authors measure the initial rate using a single time point. This is not standard in the field. How do the authors ensure that they are in the initial/linear phase?

Minor points
- The introduction should be expanded to include a couple of applications of labelled oligonucleotides. For example, fluorescent labelled oligonucleotides are important for diagnostics and as therapeutic tools (i.e. imaging).

-The use of the term 5'- and 3'- labelling is misleading. It is not the ribose 5' and/or 3' that is being labelled but the nucleobase at the first and/or last position of the sequence. This distinction is important. For example, therapeutic siRNAs are commonly labelled at the 3'-end and not on the nucleobase.

- Some sections need to be more concise. For example, the description of the steady-state kinetics in the 'Recognition of UBP dNaM-dTPT3 and its analogues by SFM4-6' section. Here a table stating the kinetic constants would aid understanding and reduce the length of the text.

- Why was thermocycling used in primer extension assays using equimolar primer/template (100 nM) and an excess of enzyme (200 nM)? For example see 'Primer extension assay for SFM4-6-mediated synthesis of RNA and 2'-modified RNA oligonucleotides incorporating a dTPT3, rTPT3, dTPT3Am, dTPT3Al or rTPT3Al nucleotide at a specific internal position using a dNaM-containing DNA template'.

- Figure 1B unclear – what are the nucleotide (monophopshates?) in the circles depicting?

Reviewer #2

(Remarks to the Author)
Wu et al. explored the ability to incorporate varies noncanonical nucleotides as the first nucleotide added to a primer as well as the ability to then add natural nucleotides downstream of the primer during primer extension. The authors specifically explore an engineered polymerase called SFM4-6 to successfully carry out the primer extension step. Robust chemical techniques to incorporate useful fluorescent dyes or other nonnucleotide moieties such as amine or biotin groups at the 5' and 3' end have proven practical for many oligonucleotide studies. Here, the key differences are multi-fold involving the ability to incorporate noncanonical nucleotide within a DNA or RNA sequence using engineered polymerases as a simpler synthesis pathway than solid state chemical approaches. These possibilities are exciting to finding simpler methods to expand the four nucleotide alphabet of oligonucleotides. Overall, the authors present an organized and systematic studies with several helpful schematics to accompany each Figure and detailed subsection titles to guide the reader through the nuances of multiple sets of experiments involving primer extension steps. Given the variety of chemical species and variation in primer extension conditions, this reviewer appreciated the ability to match Figure Captions to a similarly (though not always identically) worded subsection in the Methods section in order to follow particular experimental details in the Results and Discussion.

Overall the manuscript read well and demonstrates the overall goal, but there were a few primer extension studies that lacked clarity in either the experimental description or the results. The aptamer experiments involved either unpublished aptamer sequences by the authors or aptamer sequences, possibly structure-switching sequences for thrombin, lacking references in the main manuscript. Given the lack of both references and more detailed studies (e.g., testing conditions in which aptamer binding is not anticipated such as using non-aptamer sequences incubated with a given target) this reviewer suggests saving these initial aptamer experiments for more expanded studies in a separate future publication. To address the remainder of the manuscript in which either experimental methods or results lacked clarity for this reviewer, please address the following points or questions:

Minor, but important details that are either missing or not clear.
1. I could not find information on the source of the engineered SFM4-6 polymerase in the Materials section. Was this polymerase purchased or synthesized by the authors?
2. Given the focus on primer extension as a key technique for this study, the authors should provide important missing details such as the purity (template and primers PAGE purified? HPLC purified?), concentration, volume, etc. of each reagent in the Methods section.
3. I found it helpful to read the Methods with a subsection title (bolded text) that exactly matched the initial bolded text in a Figure Caption in order to understand the experimental nuances for a particular Figure. Thus, I recommend consistently using the exact wording in a Figure Caption to match the Methods subsection title where relevant.
4. The Figure on the page prior to Figure 1 is presumed to serve as a TOC graphic, but I could not find a description for this figure.
5. According the Methods section description, it appears that every PAGE experiment in a given Figure was a denaturing gel. The Figure caption should state if a gel was a denaturing or native gel and specifically clarify if a gel in a particular figure involving fluorescent species is imaged prior to or after SYBR gold stain (spelled as “Cyber" staining in the Methods section).
6. It is also assumed that any digestion assays with DNAse I were selectively used to test nuclease resistance in separate studies primarily presented in the SI. The aptamer studies in Figures 5 and 6 are an exception to this assumption based on the description provided in the Figure Captions. Thus, it seems that any residual or initially duplex-associated DNA template for a one-cycle primer extension step would have a visible band in denaturing PAGE unless its nucleotide length matched that of a fully extended, identical nucleotide-length partner strand. Given the apparent sensitivity to one nucleotide extension accomplished in multiple experiments (e.g., Figure 1 and Figure 3), it seems that even a slightly longer template would have a distinct band from shorter, yet fully extended partner strand. However, in Figure 4, only the fully extended partner strand band is labeled while the longer template sequence does not appear as a separate band. Can the authors explain this discrepancy?
7. Related to point #6 above (and assuming Sybr Gold staining has been performed), is the band labeled as “F” in Figure 2 just the fully extended partner strand as labeled in the Figure 2 Caption or is DNA template also present in this same band? It would have been helpful to show a separate “control” lane for the DNA template alone for this PAGE gel. For consistency, the authors need to re-label “T” as “ST” to match the “stalled primer" extension partner strand description provided in the Caption. It is also suggested to use different punctuation as a substitute for the comma in “2'-F-C,U-RNA” if both the C & U nucleotides are modified in the same RNA sequence (e.g., 2'-F-C+U-RNA).”
8. It is helpful to specifically label bands of the original primer sequence with “n” nucleotides and the “n+1” primer sequence in Figure 1. If this nomenclature is also relevant to the two sets of bands Figure 3, it would be helpful to add these same labels “n” and “n+1” bands.
9. For most of these oligonucleotides, if primer extension is the key goal requiring only one PCR cycle, why were multiple cycles of PCR used as described in lines 599-600 and lines 695-697?

Suggestions for improving the impact of the current study with select additional experiments for in vitro studies.
10. In contrast to this reviewer's suggestion to remove the aptamer studies from this manuscript, the transfection experiments in Figure 5 provide a brief, but classic demonstration of oligonucleotide functionality in which the 5' end (rather than a central nucleotide within the sequence) was fluorescently labeled via SFM4-6. Micrographs in Figure 5 are helpful to visually demonstrate transfection in a snapshot of multiple cells. However, given the use of GFP as their “biomarker”, quantitative

flow cytometry of cell populations prior to and post siRNA incubation would be a more quantitative method for analyzing transfection effects on thousands of cells. To test the sensitivity of dye activity to chemical conjugation via their engineered polymerase, it would be worthwhile to also compare fluorescence activity of their SFM4-6 modified RNA to commercial analogues using fluorescence spectroscopy. For this reviewer, this additional experiment would be a more informative example of expanding the study beyond its primary focus of noncanonical nucleotide incorporation to specifically demonstrate its additional utility and functional/property implications on incorporating chemical species such as a fluorescent dye at one end.

Other minor suggestions
11. I suggest removing the word “semi-synthetic” from the abstract (line 18).
12. The authors should define all noncanonical reagent abbreviations, especially if used in the Introduction (e.g., dNaM-dTPT3). A separate list of abbreviations would be helpful.
13. Line 73, change “Base” to “Based”
14. Line 88, change "characterize" to “characterized”
15. Line 178, change “unspecific” to “nonspecific”

Reviewer: Valeria Tohver Milam

Reviewer #3

(Remarks to the Author)
The paper by Wu et al. provides a very systematic exploration of the utility of the engineered polymerase SFM4-6 for the preparation of a number of modified RNA constructs. These constructs are typically obtained via chemical synthesis; however, their method is highly useful for preparing longer sequences, where synthetic efficiency typically drops. The paper therefore represents an important advance and should be of interest to a broad readership.
Comments:
Main Concern:
My main concern is that the performance of the method is verified using only a single technique—gel mobility shift analysis. While this is suitable for detecting changes in molecular weight, however it cannot resolve whether the polymerase incorporates incorrect or unintended bases (see also comment below). Since much of the work relies on demonstrating the successful synthesis of different oligonucleotides using SFM4-6, at a minimum, a selected subset of oligos should be verified using a complementary method—e.g., mass spectrometry or sequencing.
Other Issues:
Figure 1 could be improved:
• In Figure 1B, the bases that "work" could be shown on the left side of the long vertical line, with SFM4-6 in the middle, and those that do not work on the right. Given the number of bases tested, it's difficult to determine which is which using only the legend.
• In the bottom left of Figure 1B, the schematic seems to show the end product (n+1). It would make more sense to connect the added base (e.g., rTPT3) to the primer directly, as there is currently no covalent link shown. A similar issue appears in Figure 3A (right panel).
Non-specific incorporation of bases:
• Line 188 / Figure SI2B: What exactly is shown in lane 1? Is this template degraded by DNase I? Why does it appear to have a higher molecular weight than lanes 2–9? Aren’t these all single-stranded DNAs of similar molecular weight? Importantly, there should be a control in the gel showing results without DNase I treatment.
• Line 200: "with varied efficiency and specificity" — The reader would benefit from a brief comment here. For example: "Efficiency follows the trend X > Y > Z", as Figure S3 shows considerable differences.
• Line 158, Figure SI3, and Figure 2: What explains the faint bands in lanes 2, 3, and 6 (SI3B), and lane 6 (SI3C)? This suggests some non-specific incorporation of alternative bases. Similar observations hold for results in Figures 2Ba and 2Bb. Which base is being incorporated non-specifically? Could this be tested via mass spectrometry?
• Line 218: "with excellent efficiency" — This mainly applies to RNA synthesis. The synthesis of other variants is not as efficient, as noted in line 200.
Optimization results:
• Line 242: "under optimized conditions" — A brief comment on the outcome of the optimization should be included in the main text or figure legend. Which parameter(s) were adjusted compared to the unoptimized (i.e., standard) conditions?
Figure 9D – Thrombin aptamer sensor:
• Line 921: The text states that a solution with "unfolded" aptamer was prepared. How was this done? Which experiments verify that the aptamer was indeed unfolded?
• Regarding performance testing: At a minimum, a control is needed. For example, a sample with an unrelated protein added to the sensor, or a condition where thrombin is added to DNA lacking FRET probes, should be included.
Availability of SFM4-6:
• Since SFM4-6 has been previously characterized (ref 12), it would be helpful to include the protein sequence in the Supplementary Information, along with a note indicating whether the sequence includes any tags, fusion domains, or other modifications.

Minor Points:
• There are several typographical errors:
o Line 73: "base" → "based"
o Line 73 again: Unclear use of "Based" (please clarify)

o Line 334: "described" is misspelled
o Line 401 and elsewhere: various typos throughout; the manuscript would benefit from careful proofreading.
• Define the abbreviation dNaM-dTPT3 at its first mention (line 68).
• Line 170 and following: There is no mention of Figure 2Bb. Does the description in lines 180 refer to results shown in Figure 2Bb? This should be clarified.

Version 1:

Reviewer comments:

Reviewer #1

(Remarks to the Author)
The revised manuscript by Chen and co-workers is significantly improved and adequately addresses my comments. It is my opinion that controlled labelling of RNA products, including modified RNAs, will have wide ranging applications and will be of interest to the broad readership of Nature Communications. I am supportive of publication.

Reviewer #2

(Remarks to the Author)
This author appreciates the detailed and thoughtful response provided by the authors for each suggestion and for undertaking additional experiments requested by reviewers in the revised manuscript.

Reviewer #3

(Remarks to the Author)
Wu et al. presented their revised paper, "Engineered Polymerase-Mediated Efficient Synthesis of Site-Specifically Functionalized RNA and 2'-Modified RNA Oligonucleotides via Genetic Alphabet Expansion", in which they have adequately addressed the comments from the first revision. I believe the paper has improved.
There are a few smaller issues related to the inclusion of new data that should be corrected:
Line 216:
“and the order for the synthesis efficiencies of different unnatural base-containing oligonucleotides is as follows: …”
→ Please refer to the data where this is shown.
Mass spectrometry:
Methods / Mass spectrometry:
“…oligonucleotides were prepared as described above…”
→ Please clarify which reaction conditions this refers to: the standard 4 h conditions (S3) or the “optimized” 17 h conditions (S4)?
Mass spectra in Figures S17–S30:
• Please indicate in the caption or header which reaction/gel position in the manuscript each spectrum corresponds to. It is currently difficult to navigate due to the complex names of different oligonucleotides. For example: “Fig. S30 (refers to extension products shown in Fig. X, lane X).”
• It is helpful that “Target vs Observed mass” is included in the header of some figures → please also include this in Figures S17 and S18 so that all mass spectrometry data are presented uniformly.
• In most spectra, the mass axis (x-axis) spans ~1000 Da. Why do some spectra (S20, S21, S25) appear zoomed in (x-axis only ±200 Da)?
• For rTPT3Al RNA (Fig. S21), the observed mass is lower than expected (by >100 Da). Please explain why.
All other comments have been appropriately addressed.

Open Access This Peer Review File is licensed under a Creative Commons Attribution 4.0 International License, which permits use, sharing, adaptation, distribution and reproduction in any medium or format, as long as you give appropriate credit to the original author(s) and the source, provide a link to the Creative Commons license, and indicate if changes were made.
In cases where reviewers are anonymous, credit should be given to 'Anonymous Referee' and the source.
The images or other third party material in this Peer Review File are included in the article's Creative Commons license, unless indicated otherwise in a credit line to the material. If material is not included in the article's Creative Commons license and your intended use is not permitted by statutory regulation or exceeds the permitted use, you will need to obtain permission directly from the copyright holder.
To view a copy of this license, visit https://creativecommons.org/licenses/by/4.0/

Professor Tingjian Chen
School of Biology and Biological Engineering
South China University and Technology
Guangzhou 510006, China

+86-15096647258
chentj@scut.edu.cn

Referee 1:

Thank you very much for the careful review of our manuscript, and all the comments and suggestions are very helpful for improving the manuscript. All the comments and suggestions are addressed point by point as following, and for your convenience, all the revisions are highlighted in the marked version of the revised manuscript, which is submitted as supporting information for review.

(1) One limitation of the approach is the requirement for stoichiometric primer and DNA template, which must be produced (and purified/isolated) using either chemical or enzymatic methods. This raises the question 'Why not directly synthesize the target oligonucleotide?'. This should be explained in the introduction.

> The suggestion is well taken. Oligonucleotides, including those with modifications, are traditionally produced via solid-phase chemical synthesis. However, solid-phase chemical synthesis of RNA and modified RNA oligonucleotides has many limitations (Zhang, B., et al., *Nucleic Acids Res*., 2025). For example, the length of an RNA or modified RNA oligonucleotide that can be efficiently produced by solid-phase chemical synthesis is quite limited (typically ≤ 40 nt)(Ryczek, M. et al., *Appl. Sci.*, 2022), while the DNA template used for the enzymatic synthesis of an RNA or modified RNA oligonucleotide can be much longer and produced by PCR assembly of short DNAs if necessary. Besides, some of the modified phosphoramidites are very expensive or even not commercially available (for example, all phosphoramidites of dTPT3 and its derivatives are not commercially available). Moreover, solid-phase chemical synthesis does not synthesize an oligonucleotide by transcription from a template and thus cannot be used for the evolution of functional oligonucleotides via iterative rounds of selection (Liu, Z. et al., *Chem. Sci*., 2017 and Wang, B. et al., *Angew Chem. Int. Ed. Engl.*, 2024). Enzymatic synthesis of RNA

Professor Tingjian Chen
School of Biology and Biological Engineering
South China University and Technology
Guangzhou 510006, China

+86-15096647258
chentj@scut.edu.cn

and modified RNA oligonucleotide from a DNA template is actually what we did in this work. Although enzymatic synthesis of oligonucleotides can also be achieved via *de novo* synthesis with terminal deoxynucleotidyl transferases (TdTs) and similar enzymes, the synthesis of modified RNA oligonucleotides needs 3'-protected modified nucleoside triphosphates, some of which again are not commercially available. We have included an explanation on this in the introduction of the revised manuscript (page 3, lines 43-57).

(2) Another limitation is that the polymerase efficiency in extension reactions of sequences containing dTPT3 nucleotides containing propargylamino or ethynyl modifications is severely compromised (Fig S3). Although the enzyme can extend $dTPT^{Am}$ and $dTPT^{Al}$ using rNTPs, the conversion to fully extended product using 2'-fluoro and/or methoxy is very low. This should also be discussed in the manuscript. Do the authors have an explanation for this and could they improve the efficiency with further engineering?

> Thank you for the comment and suggestion. In Figure S3 of the original manuscript, in order to show the difference in the synthesis efficiencies of $dTPT3^{Am}$, $dTPT3^{Al}$, or $rTPT3^{Al}$-containing RNA, 2'-F-CU-RNA, 2'-F/OMe-RNA, and 2'-OMe-RNA oligonucleotides and the difference in the incorporation specificities of $dTPT3^{Am}TP$, $dTPT3^{Al}TP$, and $rTPT3^{Al}TP$ into these oligonucleotides, the primer extension reactions were carried out under conditions that are not optimal for producing the maximum amount of the full-length product for each of the oligonucleotide. Therefore, the amounts of the full-length products for $dTPT3^{Am}$, $dTPT3^{Al}$, or $rTPT3^{Al}$-containing 2'-F-CU-RNA, 2'-F/OMe-RNA, and 2'-OMe-RNA oligonucleotides, especially 2'-F/OMe-RNA and 2'-OMe-RNA oligonucleotides, were limited. The yields of the full-length products for these oligonucleotides can be significantly improved by using optimized reaction conditions,

Professor Tingjian Chen
School of Biology and Biological Engineering
South China University and Technology
Guangzhou 510006, China

+86-15096647258
chentj@scut.edu.cn

such as the reaction time was increased from 50 °C for 4 h (Fig. S3) to thermocycling program: 6 cycles of (50 °C, 2 h; 70 °C, 30 min); 50 °C, 2 h (Fig. S4). To demonstrate this, we carried out primer extension reactions for the synthesis of dTPT3^{Am}, dTPT3^{Al}, or rTPT3^{Al}-containing 2'-F/OMe-RNA and 2'-OMe-RNA oligonucleotides under optimized reaction conditions, in which the reaction time was increased from4 h (Fig. S3) to 17 h (Fig. S4), and analyzed the products by 20% denaturing PAGE gels containing 8 M urea. As shown in Supplementary Figure S4 of the revised manuscript, for each of the oligonucleotides, in the presence of dTPT3^{Am}TP, dTPT3^{Al}TP, or rTPT3^{Al}TP, the yield of the full-length product was significantly increased, and only a little or almost no byproduct was observed under these conditions We have included the experimental methods, results and related discussion in the revised manuscript (page 9, lines 219-226; page 25-26, lines 719-723, in Supplementary Information page S8-S9)

(3) For the measurement of the kinetic constants, the authors measure the initial rate using a single time point. This is not standard in the field. How do the authors ensure that they are in the initial/linear phase?

> Thank you for the comment and question. In the measurement of the kinetic constants, to ensure that each of the initial rates was measured within the linear phase of the reaction, and the extension percentage of the primer at a single time point could be used for the calculation of the initial rate, the extension percentage of the primer was controlled to be approximately 20% by quenching the reaction at a proper time for each reaction. This method was commonly used for the measurement of the steady-state kinetic constants of different polymerases (Randall, S. K. et al., *J. Biol. Chem.*, 1987; Dieckman, L. M. et al., *Biochemistry* 2010; Gahlon, H. L. et al., *Methods Mol. Biol.*, 2019). To rigorously validate

Professor Tingjian Chen
School of Biology and Biological Engineering
South China University and Technology
Guangzhou 510006, China

+86-15096647258
chentj@scut.edu.cn

that the time points that we selected to measure the initial rates (when the extension percentages of the primer were approximately 20% were indeed within the linear phase of the reaction, we carried out an experiment to measure the time course of the primer extension under identical reaction conditions for the steady-state kinetic experiments. The result clearly suggests that all the selected time points were within the linear phase of the reaction, and each of the initial rates could be calculated by the extension percentage of the primer at a single time point. We have included the experimental method, result, and related discussion in the revised manuscript (page 12-13, lines 321-336; page 28-29, lines 809-823; Supplementary Figure S10).

Minor comments:

1) The introduction should be expanded to include a couple of applications of labelled oligonucleotides. For example, fluorescent labelled oligonucleotides are important for diagnostics and as therapeutic tools (i.e. imaging).
> This suggestion is well taken. We have included a discussion on the applications of the labelled oligonucleotides in the revised manuscript (page 4, lines 69-72).

2) The use of the term 5'- and 3'- labelling is misleading. It is not the ribose 5' and/or 3' that is being labelled but the nucleobase at the first and/or last position of the sequence. This distinction is important. For example, therapeutic siRNAs are commonly labelled at the 3'-end and not on the nucleobase.
> This suggestion is well taken. To avoid any misunderstanding, we have changed the terms "5'-labelling" and "3'-labelling" to "5'-end-labelling" and "3'-end-labelling" when

Professor Tingjian Chen
School of Biology and Biological Engineering
South China University and Technology
Guangzhou 510006, China

+86-15096647258
chentj@scut.edu.cn

they are used to describe the labelling at the 5' end or 3' end of the oligonucleotides in the revised manuscript.

3) Some sections need to be more concise. For example, the description of the steady-state kinetics in the ‘Recognition of UBP dNaM-dTPT3 and its analogues by SFM4-6’ section. Here a table stating the kinetic constants would aid understanding and reduce the length of the text.
> This suggestion is well taken. We have included tables stating the kinetic constants and streamlined the description of the steady-state kinetics in the revised manuscript (Supplementary Table S2, S3, and; page 7, lines 160-166).

4) Why was thermocycling used in primer extension assays using equimolar primer/template (100 nM) and an excess of enzyme (200 nM)? For example see ‘Primer extension assay for SFM4-6-mediated synthesis of RNA and 2'-modified RNA oligonucleotides incorporating a dTPT3, rTPT3, dTPT3Am, dTPT3Al or rTPT3Al nucleotide at a specific internal position using a dNaM-containing DNA template’.
> Thank you for this question. In our previous work, we found that the use of thermocycling cycles could effectively increase the yield of the full-length product for the synthesis of 2'-OMe/F-RNA and fully modified 2'-OMe-RNA, which might be attributed to an increase in the primer extension efficiency due to the disruption of potential secondary structures in the template and extension product formed during the reaction by thermocycling (Liu, Z. et al., *Chem. Sci.*, 2017 and Qin, Y. et al., *ACS Synth. Biol.*, 2023). Therefore, a program with thermocycling cycles was applied for the synthesis of 2'-OMe/F-RNA and fully modified 2'-OMe-RNA to increase the yield of the full-length product in this work. Excess amount of the enzyme was used to avoid the decrease of the synthesis efficiency due to a

Professor Tingjian Chen
School of Biology and Biological Engineering
South China University and Technology
Guangzhou 510006, China

+86-15096647258
chentj@scut.edu.cn

decrease in the enzyme activity during the thermocycling (Qin, Y. et al., *ACS Synth. Biol.*, 2023). We have cited the original reference where the synthesis method involving the use of thermocycling cycles and excess amount of the enzyme was established in the revised manuscript (page 24-25, line 719-723; Ref. 13, 15)

5) Figure 1B unclear – what are the nucleotide (monophopshates?) in the circles depicting?
> Thank you for the comment and question. The nucleotides in the circles in Figure 1B are different natural and unnatural monophosphates that are incorporated into the 3' end of the primer by SFM4-6. We have revised Figure 1B to improve its clarity and accuracy in the revised manuscript.

Referee 2:
Thank you very much for the careful review of our manuscript, and all the comments and suggestions are very helpful for improving the manuscript. All the comments and suggestions are addressed point by point as following, and for your convenience, all the revisions are highlighted in the marked version of the revised manuscript, which is submitted as supporting information for review.

1) Overall the manuscript read well and demonstrates the overall goal, but there were a few primer extension studies that lacked clarity in either the experimental description or the results.
> Thank you for the comment. We apologize for the lacking of clarity in the experimental description or results for a few primer extension studies. We have thoroughly checked the manuscript, revised the unclear statements in the experimental description and results of the primer extension studies, and supplemented missing information in these contents in

Professor Tingjian Chen
School of Biology and Biological Engineering
South China University and Technology
Guangzhou 510006, China

+86-15096647258
chentj@scut.edu.cn

the revised manuscript (page 24, lines 666, 683; page 25, lines 695, 713; page 26, lines 733; page 27, lines 763, 776; page 28, lines 792; page 29, lines 830; page 31, lines 874; page 32, lines 911, 917-919; page 34, lines 989; page 35, lines 1020, 1026-1027; page 37, lines 1054; page 38, lines 1097; Figure 1C; Figure 2B; Figure 3B; Figure 4B; Figure 5B; Supplementary Figure S2B-E, S3B-E, S4A-B, S5B-D, S6B, S10B, S11B, S12B-D, S13B-E, S16B-C).

2) The aptamer experiments involved either unpublished aptamer sequences by the authors or aptamer sequences, possibly structure-switching sequences for thrombin, lacking references in the main manuscript. Given the lack of both references and more detailed studies (e.g., testing conditions in which aptamer binding is not anticipated such as using non-aptamer sequences incubated with a given target) this reviewer suggests saving these initial aptamer experiments for more expanded studies in a separate future publication.

> Thank you for the comment and suggestion. We have recently published the work on the *B. cereus* 5/B/6 MBL aptamers (Wu, J. et al., *ACS Synth. Biol.*, 2025), in which these aptamers have been thoroughly characterized, so we feel it should be appropriate to include the contents involving these aptamers in this manuscript now. We have cited the reference in the revised manuscript (page 3, line 55; page 16, line 451; Ref. 15). Detailed information about the thrombin aptamer can be found in a few references (White, R. et al., *Mol. Ther.*, 2001, Martha, L. et al., *FEBS Lett.*, 2004). We have also cited these references in the revised manuscript (page 55, line 427; Ref. 53 and 54). For more detailed studies on the thrombin aptasensor, we have first carried out a control experiment with a non-target protein (BSA), and the result clearly suggests the good target specificity of the aptasensor. Moreover, a group of control experiments have also been carried out with an aptamer labelled with only a 5'-end FAM under identical conditions as those for the experiments

Professor Tingjian Chen
School of Biology and Biological Engineering
South China University and Technology
Guangzhou 510006, China

+86-15096647258
chentj@scut.edu.cn

with the aptasensor. The results show that both the fluorophore (FAM) and the quencher (Dabcyl) are necessary for the function of the aptasensor, supporting that the increase in the fluorescence upon the incubation of the aptasensor with the target was likely due a conformational change of the aptasensor that increased the distance between the FAM and Dabcyl groups. The experimental methods, results, and related discussion have been included in the revised manuscript (page 20, lines 553-564; page 40, lines 1139-1142; Figure 9E).

Minor comments:

1) I could not find information on the source of the engineered SFM4-6 polymerase in the Materials section. Was this polymerase purchased or synthesized by the authors?

> Thank you for the comment and question. Polymerase SFM4-6 was expressed and purified in our lab. We have included the method for its expression and purification, as well as its protein sequence in the revised manuscript (page 23, lines 637-658; Supplementary Note S1).

2) Given the focus on primer extension as a key technique for this study, the authors should provide important missing details such as the purity (template and primers PAGE purified? HPLC purified?), concentration, volume, etc. of each reagent in the Methods section.

> This suggestion is well taken. We have included the missing details of the reagents, including the purification methods and the purities of the primers and templates, as well as their final concentrations in different reaction solutions, in the revised manuscript (page 23, lines 631-634).

Professor Tingjian Chen
School of Biology and Biological Engineering
South China University and Technology
Guangzhou 510006, China

+86-15096647258
chentj@scut.edu.cn

3) I found it helpful to read the Methods with a subsection title (bolded text) that exactly matched the initial bolded text in a Figure Caption in order to understand the experimental nuances for a particular Figure. Thus, I recommend consistently using the exact wording in a Figure Caption to match the Methods subsection title where relevant.
> This suggestion is well taken. We have revised the subsection titles in the Methods to match the wording to that used in corresponding figure captions, as well as other parts of the manuscript, where applicable.

4) The Figure on the page prior to Figure 1 is presumed to serve as a TOC graphic, but I could not find a description for this figure.
> Thank you for the comment. This figure is indeed the TOC graphic. We have included a description of it in the revised manuscript (page 48, line 1331).

5) According the Methods section description, it appears that every PAGE experiment in a given Figure was a denaturing gel. The Figure caption should state if a gel was a denaturing or native gel and specifically clarify if a gel in a particular figure involving fluorescent species is imaged prior to or after SYBR gold stain (spelled as “Cyber" staining in the Methods section).
> This suggestion is well taken. We have included information about the types of the PAGE gels, as well as if the gels were stained with Cyber Gold (an analog of SYBR Gold, as described in the Materials section) or not before imaging for gels involving fluorescent species, in the Figure captions of the revised manuscript (page 9, line 240-241, page 22, line 616).

Professor Tingjian Chen
School of Biology and Biological Engineering
South China University and Technology
Guangzhou 510006, China

+86-15096647258
chentj@scut.edu.cn

6). It is also assumed that any digestion assays with DNase I were selectively used to test nuclease resistance in separate studies primarily presented in the SI. The aptamer studies in Figures 5 and 6 are an exception to this assumption based on the description provided in the Figure Captions. Thus, it seems that any residual or initially duplex-associated DNA template for a one-cycle primer extension step would have a visible band in denaturing PAGE unless its nucleotide length matched that of a fully extended, identical nucleotide-length partner strand. Given the apparent sensitivity to one nucleotide extension accomplished in multiple experiments (e.g., Figure 1 and Figure 3), it seems that even a slightly longer template would have a distinct band from shorter, yet fully extended partner strand. However, in Figure 4, only the fully extended partner strand band is labeled while the longer template sequence does not appear as a separate band. Can the authors explain this discrepancy?

> Thank you for the question. In Figure 4, the primer used for the primer extension reactions was FAM-labelled, and thus the extended partner strand was also FAM-labelled. Since the gels were directly imaged without staining with Cyber Gold (an alternative to SYBR Gold) or any other nucleic acid dye, only the FAM-labelled species (i.e. the primer and the extended partner strand) can be seen on the gel, and the DNA template, which was not labelled with FAM, is invisible. To clarify this, we have included a statement that the gels were directly imaged without staining in the caption of Figure 4, as well as in those of other similar figures, in the revised manuscript (page 48, line 1341-1343; page 49, line 1362-1364; page 50, line 1392-1394).

7). Related to point #6 above (and assuming SYBR Gold staining has been performed), is the band labeled as “F” in Figure 2 just the fully extended partner strand as labeled in the Figure 2 Caption or is DNA template also present in this same band? It would have been

Professor Tingjian Chen
School of Biology and Biological Engineering
South China University and Technology
Guangzhou 510006, China

+86-15096647258
chentj@scut.edu.cn

helpful to show a separate "control" lane for the DNA template alone for this PAGE gel. For consistency, the authors need to re-label "T" as "ST" to match the "stalled primer" extension partner strand description provided in the Caption. It is also suggested to use different punctuation as a substitute for the comma in "2'-F-C,U-RNA" if both the C & U nucleotides are modified in the same RNA sequence (e.g., 2'-F-C+U-RNA)."

> Thank you for the questions and suggestions. The point-to-point responses are as following.

(1) Yes, the band labelled as "F" in Figure 2 is just the fully extended partner strand. Although the DNA template is of the same length as this fully extended partner strand and should be at a position close to that of this fully extended partner strand, it is invisible on the gel. This is due to the fact that the gels in Figure 2 were imaged without staining with Cyber Gold or any other nucleic acid dye, so only the FAM-labelled species, which are the primer and the extended partner strand, could be seen on the gel.

(2) As suggested, we have relabelled the "T" as "ST" to match the stalled primer extension product described in the caption.

(3) As suggested, to clearly show that both the C and U nucleotides are modified in the same RNA oligonucleotide, we have changed all of the "2'-F-C,U-RNA" to "2'-F-CU-RNA" in the revised manuscript.

8). It is helpful to specifically label bands of the original primer sequence with "n" nucleotides and the "n+1" primer sequence in Figure 1. If this nomenclature is also relevant to the two sets of bands Figure 3, it would be helpful to add these same labels "n" and "n+1" bands.

> This suggestion is well taken. We have added labels for the "n" and "n+1" bands in Figure 3B of the revised manuscript.

Professor Tingjian Chen
School of Biology and Biological Engineering
South China University and Technology
Guangzhou 510006, China

+86-15096647258
chentj@scut.edu.cn

9). For most of these oligonucleotides, if primer extension is the key goal requiring only one PCR cycle, why were multiple cycles of PCR used as described in lines 599-600 and lines 695-697?

> Thank you for the question In our previous work, we found that the use of thermocycling cycles could effectively increase the yield of the full-length product for the synthesis of 2'-OMe/F-RNA and fully modified 2'-OMe-RNA, which might be attributed to an increase in the primer extension efficiency due to the disruption of potential secondary structures in the template and extension product formed during the reaction by thermocycling (Liu, Z. et al., *Chem. Sci.*, 2017 and Qin, Y. et al., *ACS Synth. Biol.*, 2023). Therefore, a program with thermocycling cycles was applied for the synthesis of 2'-OMe/F-RNA and fully modified 2'-OMe-RNA to increase the yield of the full-length product in this work. We have cited the original reference where the synthesis method involving the use of thermocycling cycles was established in the revised manuscript (page 25-26, line 719-723; Ref. 13, Ref. 15).

10). In contrast to this reviewer's suggestion to remove the aptamer studies from this manuscript, the transfection experiments in Figure 5 provide a brief, but classic demonstration of oligonucleotide functionality in which the 5'-end (rather than a central nucleotide within the sequence) was fluorescently labeled via SFM4-6. Micrographs in Figure 5 are helpful to visually demonstrate transfection in a snapshot of multiple cells. However, given the use of GFP as their "biomarker", quantitative flow cytometry of cell populations prior to and post siRNA incubation would be a more quantitative method for analyzing transfection effects on thousands of cells. To test the sensitivity of dye activity to chemical conjugation via their engineered polymerase, it would be worthwhile to also

Professor Tingjian Chen
School of Biology and Biological Engineering
South China University and Technology
Guangzhou 510006, China

+86-15096647258
chentj@scut.edu.cn

compare fluorescence activity of their SFM4-6 modified RNA to commercial analogues using fluorescence spectroscopy. For this reviewer, this additional experiment would be a more informative example of expanding the study beyond its primary focus of noncanonical nucleotide incorporation to specifically demonstrate its additional utility and functional/property implications on incorporating chemical species such as a fluorescent dye at one end.

> These suggestions were well taken. The responses are as following.

(1) We have carried out quantitative flow cytometry experiments to analyze the cell populations before and after interference with the 5'-end-TAMRA-labelled siRNA prepared with $dTPT3^{Am}TP$ and SFM4-6. The results clearly suggest the good efficiencies of the transfection and interference with the 5'-end-TAMRA-labelled siRNA. We have included the experimental method, results, and related discussion in the revised manuscript (page 15, lines 413-418; page 34, lines 962-982; Supplementary Figure S15).

(2) We have carried out flow spectroscopy analysis of the 5'-end-TAMRA-labelled siRNA prepared with $dTPT3^{Am}TP$ and SFM4-6 and a commercial 5'-end-TAMRA-labelled RNA oligonucleotide produced by chemical synthesis. The fluorescence spectra of these two molecules were found to be nearly identical, suggesting that the activity of TAMRA was not affected by the conjugation of TAMRA-NHS with the 5'-end-$dTPT3^{Am}$-labelled RNA oligonucleotide prepared with SFM4-6. We have included the experimental method, results, and related discussion in the revised manuscript (page 15, lines 399-405; page 33, lines 934-939; Supplementary Figure S14).

11). I suggest removing the word “semi-synthetic” from the abstract (line 18).

Professor Tingjian Chen
School of Biology and Biological Engineering
South China University and Technology
Guangzhou 510006, China

+86-15096647258
chentj@scut.edu.cn

> Thank you for the suggestion. The phrase “semi-synthetic organisms” refers to the organisms that contain chemically synthesized genetic materials, specifically unnatural base pairs, which has been defined by Romesberg group (Zhang Y. et al., *Nature*, 2017) and broadly used by researchers in the field nowadays (Vivian T. D. et al., *J. Am. Chem. Soc.*, 2018, Aaron W. F. et al., *J. Am. Chem. Soc.*, 2019 and Jerod L. P. et al., *Nature Communi.*, 2021). Removal of “semi-synthetic” will totally change the meaning of the sentence, so we think it is necessary to retain it.

12). The authors should define all noncanonical reagent abbreviations, especially if used in the Introduction (e.g., dNaM-dTPT3). A separate list of abbreviations would be helpful.
> This suggestion is well taken. We have defined all of the noncanonical reagent abbreviations, including dNaM and dTPT3, at their first use in the revised manuscript (page 2, lines 21-23)

13). Line 73, change “Base” to “Based”
> Thank you for pointing out this error. We have corrected this error by changing the “Base” to “Based” in the revised manuscript (page 4, line 89)

14). Line 88, change "characterize” to “characterized”
> Thank you for pointing out this error. We have corrected this error by changing the “characterize” to “characterized” in the revised manuscript (page 5, line 105).

15). Line 178, change “unspecific” to “nonspecific”
> Thank you for pointing out this error. We have changed the “unspecific” to “nonspecific” in the revised manuscript (page 8, line 192).

Professor Tingjian Chen
School of Biology and Biological Engineering
South China University and Technology
Guangzhou 510006, China

+86-15096647258
chentj@scut.edu.cn

Referee: 3

Thank you very much for the careful review of our manuscript, and all the comments and suggestions are very helpful for improving the manuscript. All the comments and suggestions are addressed point by point as following, and for your convenience, all the revisions are highlighted in the marked version of the revised manuscript, which is submitted as supporting information for review.

Main Concern:

My main concern is that the performance of the method is verified using only a single technique—gel mobility shift analysis. While this is suitable for detecting changes in molecular weight, however it cannot resolve whether the polymerase incorporates incorrect or unintended bases (see also comment below). Since much of the work relies on demonstrating the successful synthesis of different oligonucleotides using SFM4-6, at a minimum, a selected subset of oligos should be verified using a complementary method—e.g., mass spectrometry or sequencing.

> The suggestion is well taken. We have carried out mass spectrometry analysis for the oligonucleotides synthesized by SFM4-6, and the results clearly demonstrated the target products, including site-specifically labelled 2'-F/OMe-RNA and 2'-OMe-RNA oligonucleotides incorporating a nucleotide of rTPT3^{Al}, 5'-end-dTPT3^{Am}-labelled RNA, 5'-end-dTPT3^{Al}-labelled RNA, 5'-end-rTPT3^{Al}-labelled RNA, 5'-end-dTPT3^{Am}-labelled 2'-F-CU-RNA, 5'-end-dTPT3^{Al}-labelled 2'-F-CU-RNA, 5'-end-rTPT3^{Al}-labelled 2'-F-CU-RNA, 5'-end-dTPT3^{Am}-labelled 2'-F/OMe-RNA, 5'-end-dTPT3^{Al}-labelled 2'-F/OMe-

Professor Tingjian Chen
School of Biology and Biological Engineering
South China University and Technology
Guangzhou 510006, China

+86-15096647258
chentj@scut.edu.cn

RNA, 5'-end-rTPT3^{Al}-labelled 2'-F/OMe-RNA, 5'-end-dTPT3^{Am}-labelled 2'-OMe-RNA, 5'-end-dTPT3^{Al}-labelled 2'-OMe-RNA, 5'-end-rTPT3^{Al}-labelled 2'-OMe-RNA oligonucleotides have been efficiently synthesized by SFM4-6. The experimental method and results have been included in the revised manuscript (page 9, lines 224-226; page 13, lines 348-351; Supplementary Figure S17-S29).

Other Issues:

1) Figure 1 could be improved: In Figure 1B, the bases that "work" could be shown on the left side of the long vertical line, with SFM4-6 in the middle, and those that do not work on the right. Given the number of bases tested, it’s difficult to determine which is which using only the legend.

> This suggestion is well taken. We have revised Figure 1B by placing the nucleoside triphosphates that can be efficiently incorporated opposite the dNaM nucleotide by SFM4-6 on the right side of the long vertical line, those that can not on the left side of the long vertical line, and SFM4-6 in the middle in the revised manuscript.

• In the bottom left of Figure 1B, the schematic seems to show the end product (n+1). It would make more sense to connect the added base (e.g., rTPT3) to the primer directly, as there is currently no covalent link shown. A similar issue appears in Figure 3A (right panel).

> Thank you for the suggestion. In the original Figure 1B and Figure 3A, the nucleotides shown in the circles and not connected to the 3' end of the primer represent alternative nucleotides to the one connected to the 3' end of the primer, one of which is incorporated to the 3' end of the primer in each case. We apologize for this confusing representation,

Professor Tingjian Chen
School of Biology and Biological Engineering
South China University and Technology
Guangzhou 510006, China

+86-15096647258
chentj@scut.edu.cn

and have revised Figure 1B and Figure 3A to make them more clear in the revised manuscript.

2) Non-specific incorporation of bases:

• Line 188 / Figure SI2B: What exactly is shown in lane 1? Is this template degraded by DNase I? Why does it appear to have a higher molecular weight than lanes 2–9? Aren't these all single-stranded DNAs of similar molecular weight? Importantly, there should be a control in the gel showing results without DNase I treatment.

> Thank you for the questions and suggestion. In the original Figure S2B, lane 1 is the DNA template used for primer extension for the synthesis of the RNA and 2'-modified RNA oligonucleotides, and lanes 2-9 are the RNA and 2'-modified RNA oligonucleotides produced by DNase I degradation of the primer extension products, via which the DNA primer and DNA template were removed. Since the DNA primer region (18 nt) in the primer extension products (55 nt) was degraded by DNase I treatment, so the length of the produced RNA and 2'-modified RNA oligonucleotides (37 nt) is shorter than the DNA template used for primer extension (55 nt). To more clearly compare the lengths of the primer extension products and the DNase I degradation products, we have rerun the gels to include more controls (the primer and the primer extension products without DNase I treatment) as suggested, and included the gel pictures in the revised manuscript (Figure S2B-E).

• Line 200: "with varied efficiency and specificity" — The reader would benefit from a brief comment here. For example: "Efficiency follows the trend X > Y > Z", as Figure S3 shows considerable differences.

Professor Tingjian Chen
School of Biology and Biological Engineering
South China University and Technology
Guangzhou 510006, China

+86-15096647258
chentj@scut.edu.cn

> This suggestion is well taken. Based on the gel pictures in Figure S3, the order for the synthesis efficiencies of different unnatural base-containing oligonucleotides is as following: RNA > 2'-F-CU-RNA > 2'-F/OMe-RNA > 2'-OMe-RNA. We have included discussion on this in the revised manuscript (page 9, lines 216-217).

• Line 158, Figure SI3, and Figure 2: What explains the faint bands in lanes 2, 3, and 6 (SI3B), and lane 6 (SI3C)? This suggests some non-specific incorporation of alternative bases. Similar observations hold for results in Figures 2Ba and 2Bb. Which base is being incorporated non-specifically? Could this be tested via mass spectrometry?

> Thank you for the comment and questions. The mentioned lanes are primer extension products by the synthesis of RNA, 2'-F-CU-RNA, 2'-F/OMe-RNA, or 2'-OMe-RNA with a dNaM-containing DNA template in the absence of d/rTPT3TP and their derivatives. For each of these lanes, although the extension of most of the primer was stalled at the position opposite the dNaM nucleotide in the template, a faint band was observed at the position for the full-length primer extension product, indicating that a small fraction of the primer was extended to the full length by non-specific incorporation of a natural base-containing nucleotide opposite the dNaM nucleotide in the template. However, for primer extension in the presence of dTPT3TP, rTPT3TP, or one of their derivatives, the incorporation of dTPT3TP, rTPT3TP, or one of their derivatives opposite the dNaM nucleotide is much faster than the incorporation of the natural base-containing NTPs, 2'-F-NTPs, and 2'-OMe-NTPs, the non-specific incorporation of a natural base-containing nucleotide opposite the dNaM should be significantly decreased in the primer extension product. To determine whether the prepared 2'-F/OMe-RNA and 2'-OMe-RNA oligonucleotides containing $rTPT3^{Al}$, and to identify the types of nucleotides nonspecifically inserted in the absence of $rTPT3^{Al}$, we performed mass spectrometry analysis on these synthesized oligonucleotides.

Professor Tingjian Chen
School of Biology and Biological Engineering
South China University and Technology
Guangzhou 510006, China

+86-15096647258
chentj@scut.edu.cn

The results showed that when the unnatural base rTPT3^{Al} was added, the major product of the synthesized oligonucleotides contained rTPT3^{Al}. In the absence of rTPT3^{Al}, nonspecific nucleotide insertions were observed in both the 2'-F/OMe-RNA and 2'-OMe-RNA oligonucleotides. Specifically, the 2'-F/OMe-RNA products contained nonspecifically inserted 2'-F-U nucleotides, while the 2'-OMe-RNA products contained nonspecifically inserted 2'-OMe-A nucleotides. The experimental methods, results, and related discussions have been included in the revised manuscript (page 9, lines 224-226; Supplementary mass spectrum data Fig. S17-S18).

• Line 218: "with excellent efficiency" — This mainly applies to RNA synthesis. The synthesis of other variants is not as efficient, as noted in line 200.

> Thank you for the comment. he "with excellent efficiency" in line 218 of the original manuscript was actually used to describe the site-specific labelling or functionalization of the dTPT3^{Am}, dTPT3^{Al}, or rTPT3^{Al}-containing oligonucleotides (i.e. coupling of the dTPT3^{Am}, dTPT3^{Al}, or rTPT3^{Al} with NHS or azide-conjugated molecules), which was indeed very efficient, so this description should be appropriate. As for the synthesis efficiencies of the oligonucleotides other than RNA, in Figure S3 of the original manuscript, in order to show the difference in the synthesis efficiencies of dTPT3^{Am}, dTPT3^{Al}, or rTPT3^{Al}-containing RNA, 2'-F-CU-RNA, 2'-F/OMe-RNA, and 2'-OMe-RNA oligonucleotides and the difference in the incorporation specificities of dTPT3^{Am}TP, dTPT3^{Al}TP, and rTPT3^{Al}TP into these oligonucleotides, the primer extension reactions were carried out under conditions that are not optimal for producing the maximum amount of the full-length product for each of the oligonucleotide. Therefore, the amounts of the full-length products for dTPT3^{Am}, dTPT3^{Al}, or rTPT3^{Al}-containing 2'-F-CU-RNA, 2'-F/OMe-RNA, and 2'-OMe-RNA oligonucleotides, especially 2'-F/OMe-RNA and 2'-OMe-

Professor Tingjian Chen
School of Biology and Biological Engineering
South China University and Technology
Guangzhou 510006, China

+86-15096647258
chentj@scut.edu.cn

RNA oligonucleotides, were limited. The yields of the full-length products for these oligonucleotides can be significantly improved by using optimized reaction conditions, such as the reaction time was increased from 4 h (Fig. S3) to 17 h (Fig. S4). To demonstrate this, we carried out primer extension reactions for the synthesis of dTPT3^{Am}, dTPT3^{Al}, or rTPT3^{Al}-containing 2'-F/OMe-RNA and 2'-OMe-RNA oligonucleotides under optimized reaction conditions, in which the reaction time was increased from 50 °C for 4 h (Fig. S3) to thermocycling program: 6 cycles of (50 °C, 2 h; 70 °C, 30 min); 50 °C, 2 h (Fig. S4)., and analyzed the products by 20% denaturing PAGE gels containing 8 M urea. As shown in Supplementary Figure S4 of the revised manuscript, for each of the oligonucleotides, in the presence of dTPT3^{Am}TP, dTPT3^{Al}TP, or rTPT3^{Al}TP, the yield of the full-length product was significantly increased, and only a little or almost no byproduct was observed under these conditions. We have included the experimental methods, results and related discussion in the revised manuscript (page 9, lines 219-220; page 25-26, lines 719-723)

3) Optimization results:

• Line 242: "under optimized conditions"—A brief comment on the outcome of the optimization should be included in the main text or figure legend. Which parameter(s) were adjusted compared to the unoptimized (i.e., standard) conditions

> Thank you for the comment. We realized that the use of the phrase "under optimized reaction conditions" in the original manuscript was not correct. In fact, Figure 3B itself demonstrates the result of the optimization experiments, where we systematically tested different natural DNA templates and concentrations of the unnatural nucleoside triphosphates for the efficient incorporation of a single unnatural nucleotide into the 3' end of the primer. We apologize for this incorrect statement, and have removed “under optimized reaction conditions” in the revised manuscript (page 10-11, lines 270-276)

Professor Tingjian Chen
School of Biology and Biological Engineering
South China University and Technology
Guangzhou 510006, China

+86-15096647258
chentj@scut.edu.cn

4) Figure 9D – Thrombin aptamer sensor:

• Line 921: The text states that a solution with "unfolded" aptamer was prepared. How was this done? Which experiments verify that the aptamer was indeed unfolded?

> Thank you for the questions. In the original manuscript, the word "unfolded" was used to describe the aptamer that was not through the folding process, in which it was incubated at 95°C for 10 minutes and slowly cooled down to room temperature. We apologize for this misleading expression. To avoid any misunderstanding, we have changed "unfolded" to "without folding" in the revised manuscript (page 20, lines 545; page 20, lines 559; page 39, lines 1137; page 55, lines 1554; Figure 9E). As shown in Fig. 9E, the fluorescence intensity for the aptasensor without folding is significantly higher than that for the folded aptasensor. This suggests that the aptasensor without folding was not correctly folded to bring the 3' FAM and 5' Dabcyl close to efficiently quench the fluorescence of FAM.

• Regarding performance testing: At a minimum, a control is needed. For example, a sample with an unrelated protein added to the sensor, or a condition where thrombin is added to DNA lacking FRET probes, should be included.

> This suggestion is well taken. We have added two groups of control experiments to the experiments: (1) a control experiment with BSA replacing the target protein (human α-thrombin); (2) control experiments with the 5'-end-FAM-labelled aptamer for human α-thrombin (FAM-Y-toggle-25t) replacing the aptasensor For the control experiment with BSA, the result showed that incubation of the folded Dabcyl-Y-toggle-25t-Y-FAM with BSA did not lead to an obvious change in the fluorescence, suggesting the good target specificity of the aptasensor. For the control experiments with FAM-Y-toggle-25t, no obvious difference in the fluorescence was observed among FAM-Y-toggle-25t without

Professor Tingjian Chen
School of Biology and Biological Engineering
South China University and Technology
Guangzhou 510006, China

+86-15096647258
chentj@scut.edu.cn

folding, folded FAM-Y-toggle-25t, folded FAM-Y-toggle-25t incubated with human α-thrombin, and folded FAM-Y-toggle-25t incubated with BSA, suggesting that both the fluorophore (FAM) and the quencher (Dabcyl) are necessary for the function of the aptasensor This supports that the increase in the fluorescence upon the incubation of the aptasensor with the target was likely due a conformational change of the aptasensor that increased the distance between the FAM and Dabcyl groups. We have included the experimental method, results, and related discussion in the revised manuscript (page 20, lines 553-564; page 40, line 1139-1142; Figure 9E).

5) Availability of SFM4-6:

•Since SFM4-6 has been previously characterized (ref 12), it would be helpful to include the protein sequence in the Supplementary Information, along with a note indicating whether the sequence includes any tags, fusion domains, or other modifications.

> This suggestion is well taken. We have included the protein sequence of SFM4-6 in the Supplementary Information (Supplementary Note S1), as well as the expression and purification method for SFM4-6 in the method section of the revised manuscript (page 23, lines 637-658).

Minor Points:

1) Line 73: "base" → "based"

> Thank you for pointing out this error. We have corrected this error by changing the “Base” to “Based” in the revised manuscript (page 4, line 89).

2) Line 73 again: Unclear use of "Based" (please clarify)

Professor Tingjian Chen
School of Biology and Biological Engineering
South China University and Technology
Guangzhou 510006, China

+86-15096647258
chentj@scut.edu.cn

> Thank you for the comment. We have revised this sentence to make it more clear in the revised manuscript (page 5, lines 105; page 20, lines 571).

3) Line 334: "described" is misspelled

> Thank you for pointing out this error. We have corrected this error in the revised manuscript (page 13, line 342).

4) Line 401 and elsewhere: various typos throughout; the manuscript would benefit from careful proofreading.

> Thank you for pointing out this and other typos in the manuscript. We have correct this typo (page 17, lines 471-475), carefully checked the whole manuscript, and corrected all other typos in the revised manuscript ((page 4, line 89; page 13, line 342)

5) Define the abbreviation dNaM-dTPT3 at its first mention (line 68).

> This suggestion is well taken. We have added the abbreviation of dNaM and dTPT3 at their first mention in the revised manuscript (page 2, line 21-23).

6) Line 170 and following: There is no mention of Figure 2Bb. Does the description in lines 180 refer to results shown in Figure 2Bb? This should be clarified.

> The suggestion is well taken. We have mentioned Figure 2Bb in the revised manuscript (page 8, line 188).

Thank you very much for your consideration.

Sincerely yours,

Professor Tingjian Chen
School of Biology and Biological Engineering
South China University and Technology
Guangzhou 510006, China

+86-15096647258
chentj@scut.edu.cn

Tingjian Chen

Professor Tingjian Chen
School of Biology and Biological Engineering,
South China University of Technology

Professor Tingjian Chen
School of Biology and Biological Engineering
South China University and Technology
Guangzhou 510006, China

+86-15096647258
chentj@scut.edu.cn

Referee 3:

Thank you very much for the careful review of our manuscript, and all the comments and suggestions are very helpful for improving the manuscript. All the comments and suggestions are addressed point by point as following, and for your convenience, all the revisions are highlighted in the marked version of the revised manuscript, which is submitted as supporting information for review.

1) Line 216: "and the order for the synthesis efficiencies of different unnatural base-containing oligonucleotides is as follows: …" Please refer to the data where this is shown.
> Thank you for the comment. We have added the reference to the corresponding data at the end of this sentence in the revised manuscript (line 214, Supplementary Fig. 3.)

Mass spectrometry:
2) Methods / Mass spectrometry: "…oligonucleotides were prepared as described above…" Please clarify which reaction conditions this refers to: the standard 4 h conditions (S3) or the "optimized" 17 h conditions (S4)?
> Thank you for the comment. The samples for mass spectrometry analysis were prepared under the optimized reaction conditions for producing products shown in Supplementary Fig. 4 (the total reaction time was 17 h). We have added clear statement in the Methods section of the revised manuscript (lines 910-912).

Mass spectra in Figures S17–S30:
3) Please indicate in the caption or header which reaction/gel position in the manuscript each spectrum corresponds to. It is currently difficult to navigate due to the complex names

Professor Tingjian Chen
School of Biology and Biological Engineering
South China University and Technology
Guangzhou 510006, China

+86-15096647258
chentj@scut.edu.cn

of different oligonucleotides. For example: "Fig. S30 (refers to extension products shown in Fig. X, lane X)."

> Thank you for the suggestion. We have added the statement on the corresponding reaction and gel band position each mass spectrum corresponds to in the figure caption of each mass spectrum in the revised manuscript (Supplementary Fig. 17-30).

4) It is helpful that "Target vs Observed mass" is included in the header of some figures please also include this in Figures S17 and S18 so that all mass spectrometry data are presented uniformly.

> Thank you for the suggestion. We have added the target mass and the observed mass of the analyzed oligonucleotide in the header of Supplementary Fig. 17 and 18 of the revised manuscript.

5) In most spectra, the mass axis (x-axis) spans ~1000 Da. Why do some spectra (S20, S21, S25) appear zoomed in (x-axis only ±200 Da)?

> Thank you for the question. The difference in the x-axis spans across the spectra is due to the fact that the amounts of the oligonucleotide samples were not fixed, and the mass spectrometry software automatically adjusted the x-axis span based on the absolute intensity of the detected signals.

6) For rTPT3^{Al} RNA (Fig. S21), the observed mass is lower than expected (by >100 Da). Please explain why.

> Thank you for pointing out this. The observed mass of the oligonucleotide in Supplementary Fig. 21 is actually 9112.8, as labeled above the peak in the spectrum by the mass spectrometry analysis software. We mistakenly wrote 9012.8 in the header of this

Professor Tingjian Chen
School of Biology and Biological Engineering
South China University and Technology
Guangzhou 510006, China

+86-15096647258
chentj@scut.edu.cn

figure in the original manuscript. We apologize for this error and have corrected it in the revised manuscript (Supplementary Fig. 21).

Thank you very much for your consideration.

Sincerely yours,

Professor Tingjian Chen
School of Biology and Biological Engineering,
South China University of Technology
